# TBACN-Promoted Regioselective Cyanofunctionalization and Benzannulation: Enabling Access to Cyanoindolizine Scaffolds via Alkyne Cyclization

**DOI:** 10.1021/acsomega.5c00775

**Published:** 2025-05-05

**Authors:** Sergen Gul, Karina S. I. Amudi, Burak Kuzu, Nurettin Menges

**Affiliations:** ‡ Science and Technology Research and Application Center (BITAM), Necmettin Erbakan University, 42100 Konya, Türkiye; § Pharmaceutical Chemistry Section, Van Yüzüncü Yil University, 65080 Van, Türkiye; ∥ Faculty of Engineering, Division of Biomedical Engineering, Necmettin Erbakan University, 42100 Konya, Türkiye

## Abstract

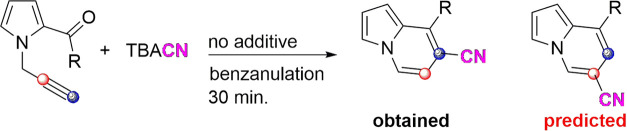

A novel and regioselective cyanofunctionalization–benzannulation cascade reaction has been developed, utilizing tetrabutylammonium cyanide (TBACN) as a practical and efficient cyanide source. This transformation provides streamlined access to a structurally diverse array of cyano-substituted indolizine scaffolds, which are valuable intermediates in the synthesis of nitrogen-containing heterocycles with potential pharmaceutical applications. The methodology employs readily available *N*-propargyl pyrrole derivatives as starting materials and proceeds under relatively mild reaction conditions, enabling the synthesis of 20 structurally distinct cyanoindolizine derivatives. The reaction exhibits remarkable regioselectivity in the installation of the cyano group, a feature that was not initially anticipated. This unexpected regioselective outcome was elucidated through a combination of control experiments, by-product analysis, and intermediate isolation, shedding light on the underlying mechanistic pathway. Furthermore, the reaction displays a broad substrate scope, demonstrating high functional group tolerance with respect to both electronic and steric variations on the pyrrole ring and the propargyl substituents. The versatility of the methodology is further highlighted by the potential for downstream transformations of the cyano group into other functional groups, such as amide moieties, which expand the synthetic utility of the obtained scaffolds. Importantly, this work represents the first reported example of a TBACN-mediated benzannulation of propargyl units, marking a significant advancement in the field of heterocyclic chemistry. The strategy not only provides a novel route to access complex indolizine frameworks but also offers valuable mechanistic insights and synthetic opportunities for the design and development of biologically relevant heterocycles.

## Introduction

**Figure 1:**
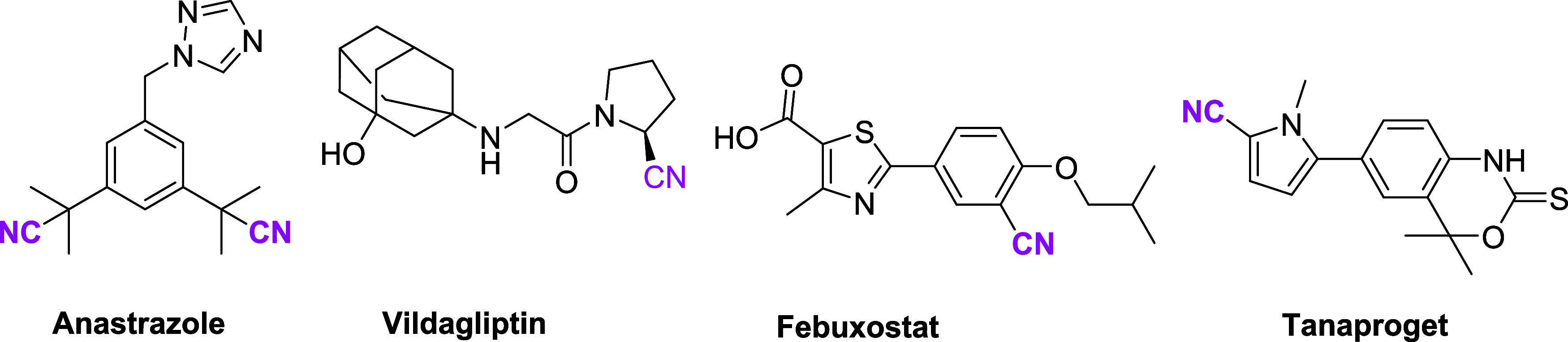
Some clinically used medicines bear the CN functionality.

Alkyne cyclization has been widely employed in various chemical reactions, often involving intriguing rearrangements facilitated by transition metals.^1a−1f^ Cyanofunctionalization of alkynes, in particular, has garnered significant attention from many research groups^2a−2d^ due to the prevalence of nitrile functionality in several clinically used drugs (Figure 1). Its versatility lies in its ability to be readily transformed into nitrogen-containing heterocycles. One notable advantage of the nitrile group is its role as a precursor to a variety of functional groups, including amides, amines, esters, carboxylic acids, ketones, aldehydes, and alcohols, which are fundamental building blocks in numerous pharmaceuticals and bioactive compounds. This versatility makes it invaluable for the synthesis of pharmaceuticals, pesticides, and organic materials.^3a−3c^

While recognized techniques like Sandmeyer and Rosenmund-von Braun can produce nitrile derivatives, cyanide sources like 2,3-dichloro-5,6-dicyano-1,4-benzoquinone (DDQ),^4a^ benzyl cyanide,^4b^ trimethylsilyl cyanide (TMSCN),^4c^ acetonitrile,^4d^ dimethylformamide (DMF),^4e^ and *N-*cyano-*N*-phenyl-*p*-toluenesulfonamide (NCTS)^4f^ can also be employed for the same purpose.

**Scheme 1:**
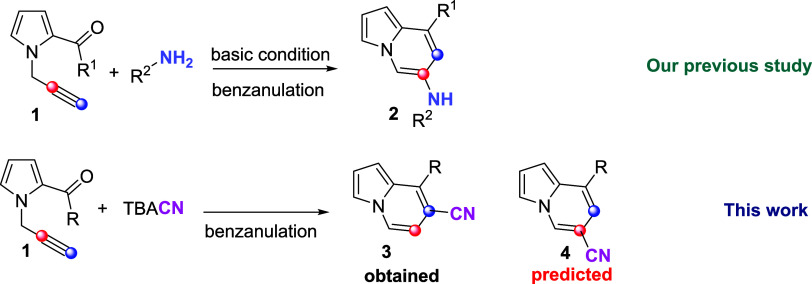
Our Previous Study and Synthetic Strategy of This Work

Our research group has previously explored the cyclization reactivity of alkyne-functionalized derivatives through a series of transformations.^5a−5d^ In one of our studies, we reported the cyclization of *N*-propargyl-pyrrole derivatives with adamantylamine and *tert*-butylamine, leading to the synthesis of an indolizine core bearing a secondary amine functionality (see molecule 2, Scheme 1). This transformation represents a valuable strategy for the construction of nitrogen-rich heterocyclic scaffolds, highlighting the synthetic versatility of *N*-propargyl-pyrrole frameworks in accessing structurally diverse and functionally relevant heterocycles.^6,7b^

The broad scope of cyclization reactions involving *N*-propargyl pyrrole derivatives and amines prompted us to investigate novel benzannulation strategies employing diverse nucleophiles. Within this framework, we aimed to utilize cyanide anions as nucleophilic partners to construct a library of cyano-substituted indolizine scaffolds, a class of compounds that remain relatively underexplored in the literature. A structurally related compound was previously reported by Zhang et al., who employed pyrrole-2-carbaldehydes and 4-bromobut-2-enenitrile in the presence of K_2_CO_3_ and dry DMF.^11a^ Additionally, Chandrashekharappa et al. described the synthesis of 7-cyanoindolizines via the reaction of pyridinium bromide with ethyl pentynoate under K_2_CO_3_-mediated conditions.^11b^ Despite these notable precedents, the design and development of a benzannulation reaction utilizing a propargyl unit in combination with TBACN as a cyanide source have not yet been reported. In this study, we present a novel cyanofunctionalization-benzannulation protocol, explore its substrate scope, and provide mechanistic insights through control experiments and by-product analysis. Importantly, this work constitutes the first example of 7-cyanoindolizine derivatives synthesized via a TBACN-mediated transformation of propargylated substrates, offering a valuable contribution to the synthetic toolbox for nitrogen-containing heterocycles.

## Experimental Section

### General Information

C-2-substituted pyrrole derivatives were obtained from SAFF Chemical Reagent (www.saffchemical.com) and used as received without further purification. *N*-Propargylated pyrrole derivatives were synthesized according to our previously reported method. ^1^H- and ^13^C NMR spectra were recorded at 400 and 100 MHz, respectively, using a Varian-Agilent 400 MHz spectrometer. High-resolution mass spectra (HRMS) were obtained on a Thermo Scientific Q Exactive MS/MS system equipped with an electrospray ionization (ESI) source. Chemical shift multiplicities are reported as follows: s = singlet, d = doublet, t = triplet, dd = doublet of doublets, m = multiplet.

### General Procedure for the Synthesis of Compounds **1a–t**

To a solution of substituted pyrrole compounds (1.1 mmol) in DMF (5 mL), sodium hydride (NaH, 60% dispersion in mineral oil, 1.8 mmol) was added portion-wise at 0 °C over the course of 1 h. The reaction mixture was then stirred at 0 °C for an additional 30 min. A solution of propargyl bromide (1.4 mmol) in DMF (1 mL) was subsequently added dropwise over 30 min. The mixture was stirred at room temperature for 16 h. Upon completion, the reaction was quenched by the addition of water (50 mL) and extracted with ethyl acetate (EtOAc, 4 × 25 mL). The combined organic layers were washed with brine (6 × 15 mL), dried over anhydrous magnesium sulfate (MgSO_4_), and concentrated under reduced pressure. The crude product was purified by silica gel column chromatography, eluting with a mixture of hexane/EtOAc (5:1).

### General Procedure for the Synthesis of Compounds **3a–u**

A solution of the pyrrole derivative (**1a–u**) (1 mmol) in anhydrous 1,4-dioxane (4 mL) was prepared. To this solution, anhydrous magnesium sulfate (200 mg) and TBACN (1 mmol) were added. The reaction tube was then sealed, and the reaction mixture was heated to 120 °C in an oil bath. Reaction progress was monitored by TLC, and completion was typically observed after approximately 30 min. Upon completion, the mixture was allowed to cool to room temperature and was subsequently extracted with ethyl acetate (3 × 10 mL) and water (30 mL). The combined organic layers were dried over anhydrous MgSO_4_, filtered, and concentrated under reduced pressure. The crude product was purified by column chromatography on silica gel, eluting with *n*-hexane:ethyl acetate (20:1).

## Results and Discussion

Preliminary crude NMR analysis from our study indicated the successful formation of an indolizine ring, and subsequent purification and detailed NMR studies were conducted to fully characterize the product. Interestingly, NMR results revealed that the nitrile functional group was not located in the predicted position (Scheme 1, compound **4**). The product’s structure was confirmed based on the coupling constant (7.1 Hz) observed for two neighboring hydrogen atoms on the pyridine ring, indicative of ortho coupling. This finding established that the CN group was attached to the terminal carbon of the alkyne group as a result of the reaction between *N*-propargyl pyrrole and TBACN (Scheme 1, compound **3**). Further optimization experiments were performed by varying CN sources, solvents, and reaction conditions, with the results summarized in Table 1.

**Table 1:**
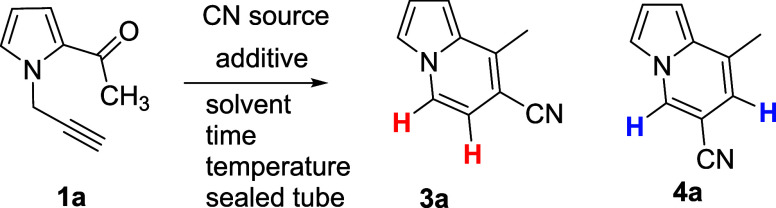
Attempts to Determine Optimized Reaction Conditions

entry	solvent	temperature^b^	^–^CN/additive	time	product
1	EtOH	120	TBACN	3 h	**1a** 5%; **3a** 15%; **5a** 80%; **7a** 5%
2	CHCl_3_	120	TBACN	1 h	**1a** 90%; **3a** 10%
3	MeCN	120	TBACN	3 h	**1a** %50; **3a** 50%
4	THF	120	TBACN	6 h	**1a** 30%; **3a** %20; **5a** 50%
5	1,4-dioxane	120	TBACN	1 h	**3a** 50%; **5a** 40%; **6a** 10%,
**6**^c^	**1,4-dioxane**	**120**	**TBACN MgSO**_**4**_	**30 min**	**3a 75%; 5a 20%; 8a 5%**
7	1,4-dioxane	120	MgSO_4_	30 min	-a
8	1,4-dioxane	120	TBACN MgSO_4_	2 h	**3a** 75%; **5a** 15%; **8a** 7%
9	1,4-dioxane	120	NaCN	3 h	-a
10	ethylene glycol	reflux	KCN	3 h	-a
11	1,4-dioxane	reflux	CuCN	3 h	-a
12	ethylene glycol	reflux	KCN, TBAI	24 h	**3a** 55%, **7a** 25%
13	ethylene glycol	reflux	KCN, AcOH	24 h	**5a** 8%, **7a** 14%
14	1,4-dioxane	120	TMSCN MgSO_4_	1 h	**-**a
15	1,4-dioxane	120	DDQ MgSO_4_	1 h	**-**a
16	1,4-dioxane	120	benzoyl cyanide MgSO_4_	1 h	**-**a

a Starting material was recovered.

b Temperature represents the temperature of the oil bath. NR: no reaction.

c 1 mmol pyrrole derivative, 1 mmol TBACN, and 200 mg MgSO_4_ were used.

### Effect of Solvent

The solvent played a crucial role in maximizing the yield of **3a**. The highest yield (75%) was observed in 1,4-dioxane with TBACN and MgSO4 (entry 6), while ethanol (entry 1) and acetonitrile (entry 2) also showed moderate yields. In contrast, ethylene glycol, chloroform, and tetrahydrofuran either produced lower yields or led to incomplete reactions.

### Effect of Cyanide Source

Tetrabutylammonium cyanide (TBACN) was the most effective cyanide source, yielding the highest amounts of **3a**. NaCN (entry 9) and CuCN (entry 11), TMSCN (entry 14), DDQ (entry 15), and benzoyl cyanide (entry 16) did not yield any significant products, indicating their inefficiency under the tested conditions. The combination of KCN with TBAI in ethylene glycol (entry 12) led to a 55% yield of **3a**. These findings suggest that the supply of CN is critical and that the tetrabutylamine group may play a critical role in the cyclization reaction.

### Effect of Additive

The addition of MgSO_4_ in 1,4-dioxane significantly improved the selectivity for **3a**, increasing its yield to 75%. This suggests that the drying effect or ion interaction of MgSO_4_ might play a role in improving the reaction efficiency. However, when MgSO_4_ was used alone, no reaction was observed.

### Effect of Temperature and Reaction Time

Higher temperatures (120 °C) were generally necessary for product formation. Lower temperatures or shorter reaction times resulted in incomplete reactions or low selectivity. Notably, shorter reaction times with TBACN and MgSO_4_ still yielded high amounts of **3a**, which made these conditions preferable.

During the optimized reaction, *N*H-pyrrole **5a** (Table S1 in the Supporting Information),^7a^ allene derivative **6a**(7b) (Table S1 in the Supporting Information), and dimerization product **7a**(7c) (Table S1 in the Supporting Information) were also formed under different reaction conditions.

**Scheme 2:**
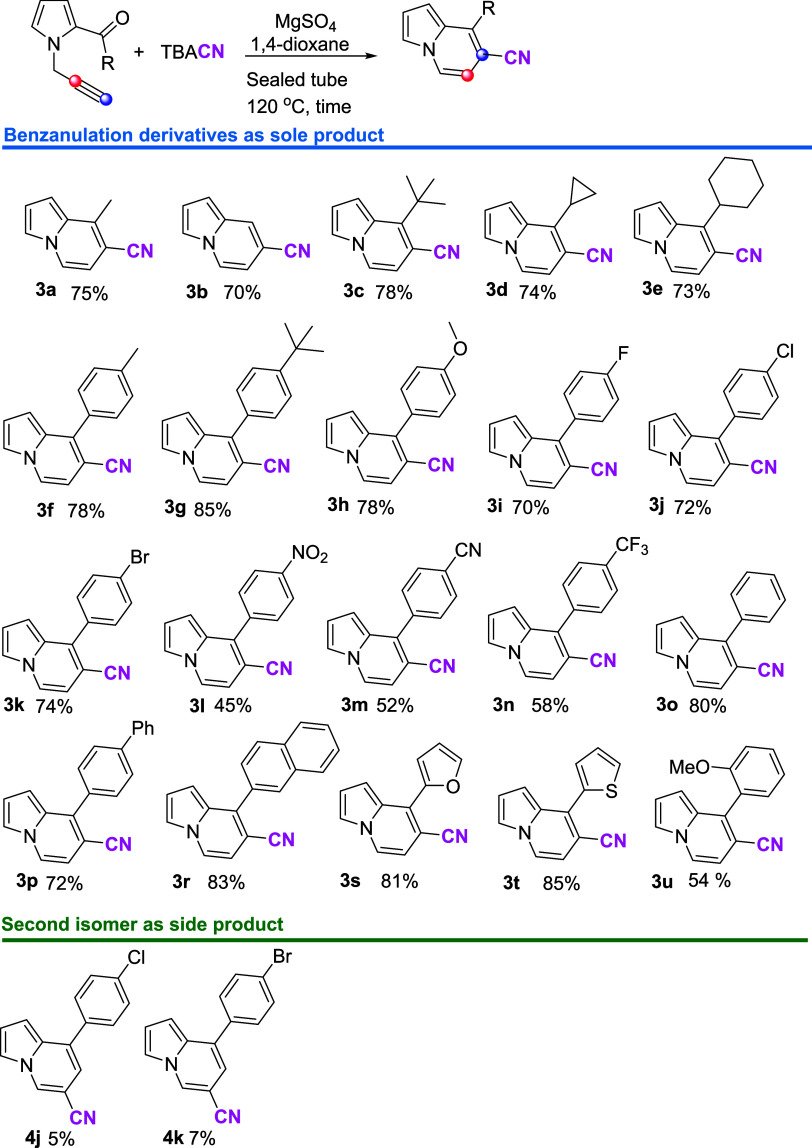
Derivatization of the Revealed Benzanulation Reaction Using 1 mmol Pyrrole Derivative, 1 mmol TBACN, and 200 mg MgSO_4_

The range of the cyclization process was examined by utilizing optimum reaction conditions. Then, to explore the behavior of the succeeding reaction conditions against electronic and steric effects, many alternative substituents in the carbonyl group positioned at the C-2 position were used. Benzanulation reactions of derivatives containing alkyl group, cyclopropyl, cyclohexyl, *tert*-butyl, and benzene ring with electron-withdrawing and electron-donating groups and different heterocyclic rings were investigated. Eventually, 20 new cyanofunctionalized indolizine compounds were synthesized with high yields (Scheme 2). Nevertheless, as a result of the cyclization processes of molecules **1j**–**k**, another isomer of cyanoindolizine (**4j** and **4k**, Scheme 2) was found. This observation verifies the reaction mechanism’s predicted route b (Scheme 4).

The derivatization reaction revealed that the electronic effect and steric hindrance had some influence on the yield of benzanulation products. A benzanulation reaction was seen with bicyclic units, naphthalene, heterocyclic rings, furan, and thiophene. While electron-donating and electron-withdrawing substituents were permitted, the yields of those reactions were reliant on electron-withdrawing groups on the benzene ring. The lowest yield, for example, was found when the nitro group was on the benzene ring, yielding 45% (**3l**).

**Scheme 3:**
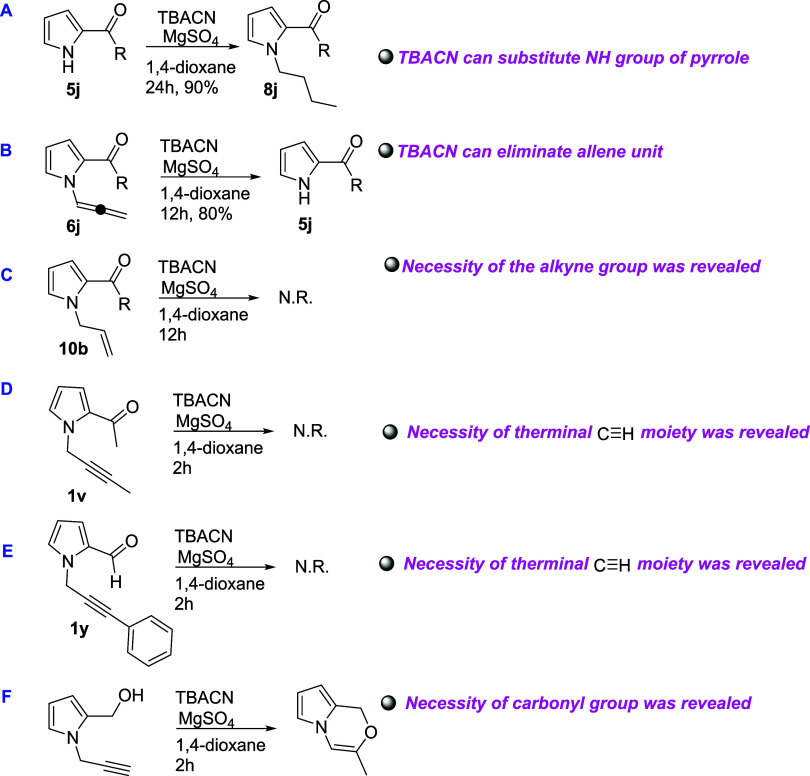
(A) Reaction of Pyrrole-*N*H with TBACN; (B) Reaction of Pyrrole-*N*-allene with TBACN. R: *p-*Cl-Ph; (C) Testing of Allyl Unit with TBACN; (D,E) Testing of Terminal Substitution with Me and Ph Groups; (F) Reaction of Reduced Pyrrole with TBACN

To further investigate the reaction mechanism, a series of control experiments were conducted using structurally modified substrates (Scheme 3 A–E). In experiment A, it was observed that TBACN could successfully substitute the NH group of the pyrrole ring, yielding the corresponding butylated product (**8j)** in a high yield (90%). Experiment B demonstrated that TBACN can also promote the elimination of the allene unit from compound **6j**, providing the simplified product **5j** in 80% yield. Notably, experiment C, in which the alkyne group was absent, resulted in no reaction (N.R.), indicating the critical role of the alkyne functionality in the transformation. Further supporting this observation, substrates bearing terminal alkynes substituted with methyl (**1v**) or phenyl (**1y**) groups (experiments D and E, respectively) also showed no conversion, with starting materials being recovered unchanged. The reaction between TBACN and reduced carbonyl derivative of the pyrrole gave pyrrolooxazine derivative^7b^ (Scheme 3F) instead of expected cyclization. These findings collectively suggest that the presence of a free terminal alkyne group is essential for the reaction to proceed, and any substitution at this position inhibits the transformation. The ability of TBACN to selectively engage with specific functional groups in the substrate also provides mechanistic clues about the reaction pathway. In addition, thanks to the by-products (**4j** and **4k**) and the independent experiments (Scheme 3A–F), we are able to suggest a suitable reaction mechanism.

**Scheme 4:**
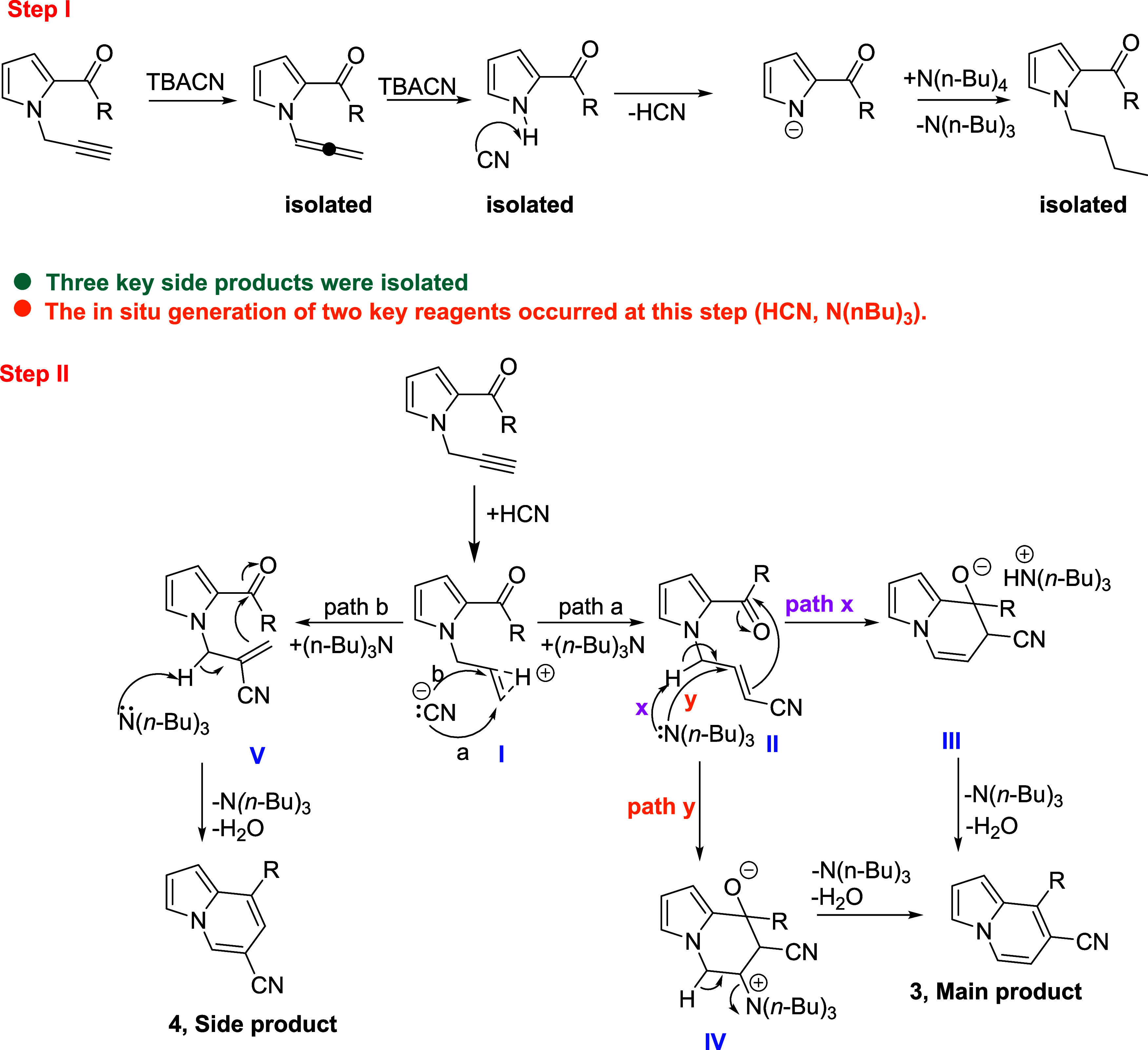
Proposed Reaction Mechanism for the Benzanulation Reaction

The reaction mechanism drawn below is suggested based on the information obtained during the control experiments and optimization studies (Scheme 4). The proposed reaction mechanism consists of two major stages (Steps I and II), each playing a crucial role in the formation of the main and side products. Extensive mechanistic studies, including the isolation of key intermediates, have been carried out to elucidate the transformation pathway and understand the selectivity observed in the final product distribution.

In the first step, it is proposed that the propargyl group is transformed into an allene derivative through the action of TBACN. Subsequently, the allene moiety is cleaved from the molecule, again facilitated by TBACN. The presence of the allene intermediate has been confirmed through a detailed investigation of the reaction medium (Table S1), and this species was successfully isolated. Moreover, the hypothesis that the allene unit dissociates from the pyrrole ring has been validated by independent experimental studies (see Scheme 3B). The NH proton of pyrrole-C-2-carbonyl compound is abstracted by the cyanide ion,^8^ leading to the formation of HCN. The anionic pyrrole species generated in situ undergoes nucleophilic substitution with the tetrabutylammonium (TBA) cation, resulting in the formation of an *N*-butylated pyrrole-C-2-carbonyl derivative, which was isolated directly from the reaction medium. The occurrence of *N*-alkylation via TBACN was independently corroborated through a control experiment, as depicted in Scheme 3A.

In the second step, the reaction may have continued with the activation of the alkyne group in the acidic environment^9^ and the attack of the CN ion on the reaction intermediate^10a−10c^ with two different possibilities (stage I). Even if the cyanide ion might attack two different carbon atoms, it is estimated that a regioselective reaction takes place with the possibility of choosing the path **a** as a sole product when path **b** gives the side product (stage V), which was also isolated for **4j** and **4k**. We think that pyrrole reacts with the tetrabutylamine unit and *N*-butyl-pyrrole and tributylamine form. The isolation of the *N*-butyl pyrrole (**7**) has been vital evidence to support this step. Two different possibilities of tributylamine attack are estimated. First, the pathway might be the abstraction of acidic hydrogen of the acrylonitrile moiety, followed by attack of the double bond to the carbonyl group (pathway **x**). This mechanism was also proposed by Wang and his co-workers.^11^ Second, the reaction pathway might be an example of the Morita–Baylis–Hillman reaction,^12a−12c^ in which benzanulation occurs with the attack of tributhylamine to the β-position of the α,β-unsaturated system (stage II, pathway **y**). The last two steps of paths a and b are the elimination of tributhylamine and H_2_O (stage III), which gives CN-functionalized indolizine derivatives, **3** and **4**.

**Scheme 5:**
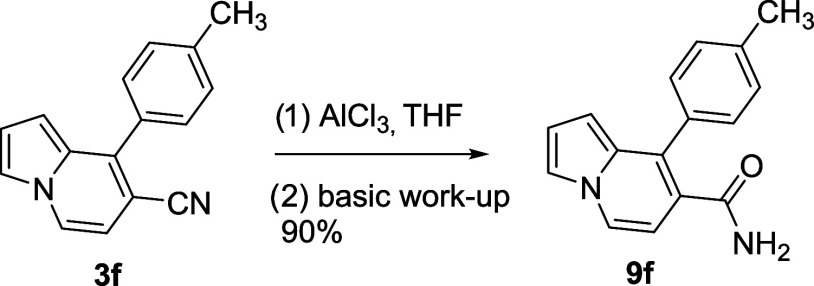
Conversion of the CN Group to Amide Functionality

The acidic hydrolysis reaction was carried out for further reactions of the CN functional group on the indolizine. As a result of this reaction, the conversion of the CN group to the amide functionality was revealed (Scheme 5).

## Conclusions

Unlike the cyclization in the literature, a new approach for the benzanulation reaction of the *N*-propargyl pyrrole molecule was reported in this study. Contrary to the results we have seen in our previous studies, the benzanulation reaction, resulting in the attack of the nucleophile group (CN group) on the terminal atom of the alkyne moiety, represents the first example in the literature. In this way, 20 different indolizine derivatives with the CN group were synthesized. Thanks to the by-products isolated during optimization experiments and two different control experiments, an assumed mechanism has been proposed. In the cyclic products obtained, the conversion of the CN functionality to a different group was tested, and the conversion to the amide group was completed with high efficiency. We continue our research on a more detailed mechanistic study of the obtained reaction and make it a more general method by applying it to different starting compounds.

## Supporting Information Available

Experimental methods; spectra of **1a–u**, **3a–u**, **4j**, **4k**, **3a–u**; ^1^H NMR and ^3^C NMR data of all synthesized compounds (PDF)

## Dedication

This article is dedicated to Emeritus Prof. Dr. Metin BALCI on his 77th birthday, in appreciation of his lifelong contributions to organic chemistry and scientific mentorship.

## References

[ref1] aGodoi B.; Schumacher R. F.; Zeni G. Synthesis of Heterocycles via Electrophilic Cyclization of Alkynes Containing Heteroatom. Chem. Rev. 2011, 111, 2937–2980. 10.1021/cr100214d. 21425870

[ref2] aSakata N.; Sasakura K.; Matsushita G.; Okamoto K.; Ohe K. Copper-Catalyzed Regio- and Stereoselective Iodocyanation and Dicyanation of Alkynes with Cyanogen Iodide. Org. Lett. 2017, 19, 3422–3425. 10.1021/acs.orglett.7b01378. 28657324

[ref3] aSamsonowicz-Górski J.; Kowalczyk P.; Koszelewski D.; Brodzka A.; Szymczak M.; Kramkowski K.; Ostaszewski R. The Synthesis and Evaluation of Amidoximes as Cytotoxic Agents on Model Bacterial *E. coli* Strains. Materials 2021, 14, 7577 10.3390/ma14247577. 34947169 PMC8708467

[ref4] aCheng J.; Chen F.; Zhang G.; Chen S.; Fei H. Copper-Catalyzed Cyanation of Arylboronic Acids Using DDQ as Cyanide Source. Synlett 2012, 23, 2247–2250. 10.1055/s-0031-1290451.

[ref5] aGüçlü D.; Kuzu B.; Tozlu I.; Taspınar F.; Canpınar H.; Taşpınar M.; Menges N. Synthesis of novel imidazopyridines and their biological evaluation as potent anticancer agents: A promising candidate for glioblastoma. Bioorg. Med. Chem. Lett. 2018, 28, 2647–2651. 10.1016/j.bmcl.2018.06.033. 30042044

[ref6] Sari O.; Seybek A. F.; Kaya S.; Menges N.; Erdem S. S.; Balci M. Mechanistic Insights into the Reaction of N-Propargylated Pyrrole- and Indole-Carbaldehyde with Ammonia, Alkyl Amines, and Branched Amines: A Synthetic and Theoretical Investigation. Eur. J. Org. Chem. 2019, 2019, 5261–5274. 10.1002/ejoc.201900084.

[ref7] aKuzu B.; Tan M.; Gülçin İ.; Menges N. A novel class for carbonic anhydrases inhibitors and evaluation of their non-zinc binding. Arch. Pharm. 2021, 354, 2100188 10.1002/ardp.202100188. 34096646

[ref8] Kuzu B.; Ekmekci Z.; Tan M.; Menges N. Excited State Intramolecular Proton Transfer (ESIPT)-Based Sensor for Ion Detection. J. Fluoresc. 2021, 31, 861–872. 10.1007/s10895-021-02716-1. 33772405

[ref9] Yamamoto Y.; Gridnev I. D.; Patil N. T.; Jin T. Alkyneactivation with Brønsted acids, iodine, or gold complexes, and its fate leading to synthetic application. Chem. Commun. 2009, 34, 5075–5087. 10.1039/b909978f. 20448954

[ref10] aDorel R.; Echavarren A. M. Gold(I)-Catalyzed Activation of Alkynes for the Construction of Molecular Complexity. Chem. Rev. 2015, 115, 9028–9072. 10.1021/cr500691k. 25844920 PMC4580024

[ref11] aZhang X.-S.; Wang B.; Jia J.; Ge Y.-Q.; Wang J. Synthesis of 7-cyanoindolizine derivatives via a tandem reaction, Heterocycl. Commun. Heterocycl. Commun. 2017, 23, 71–74. 10.1515/hc-2016-0223.

[ref12] aBaylis A. B.; Hillman M. E. D. German Patent DE2155113.1972.

