# Supplementary material for: TBACN-Promoted Regioselective Cyanofunctionalization and Benzannulation: Enabling Access to Cyanoindolizine Scaffolds via Alkyne Cyclization
Source: ACS Omega. 2025 May 5;10(18):18881–8. doi: 10.1021/acsomega.5c00775 (PMC12079246; doi:10.1021/acsomega.5c00775)
Supplement: Supplementary file 1 — ao5c00775_si_001.pdf [file ao5c00775_si_001.pdf]

SUPPORTING INFORMATION

TBACN-Promoted Regioselective Cyanofunctionalization and Benzannulation: Enabling Access to Cyanoindolizine Scaffolds via Alkyne Cyclization ‡

Sergen Gul,[1] Karina S.I. Amudi,[1] Burak Kuzu,[2] Nurettin Menges[3,*]

[1] Science and Technology Research and Application Center (BITAM), Necmettin Erbakan University, 42100, Konya, Türkiye

[2]Pharmaceutical Chemistry Section, Van Yüzüncü Yil University, 65080, Van, Türkiye

[3] Faculty of Engineering, Division of Biomedical Engineering, Necmettin Erbakan University, 42100, Konya, Türkiye

*Corresponding author: nurettin.menges@erbakan.edu.tr

Experimental Section **S2**

Table S1 **S4**

Spectral data for the synthesized compounds **S7**

Copies ^{1}H NMR and ^{13}C NMR spectra **S19**

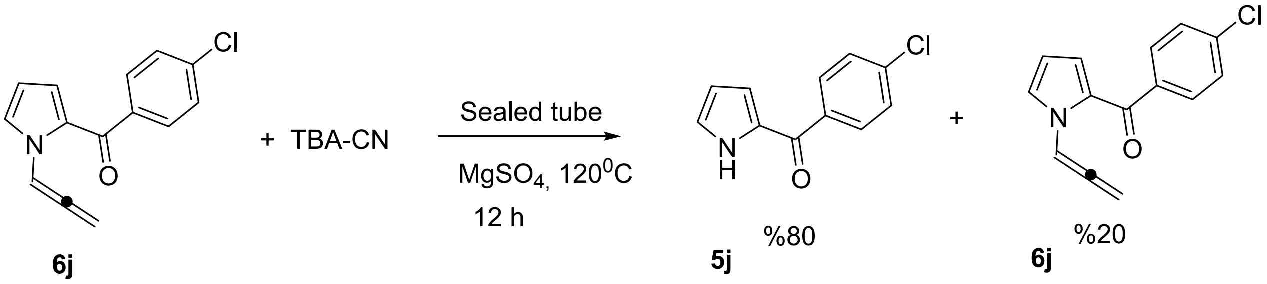

Scheme S1. Reaction of allene derivative with TBACN

A solution of the allene derivative 6j (1 mmol) in anhydrous 1,4-dioxane (4 mL) was prepared. Anhydrous magnesium sulfate (50 mg) and TBACN (1 mmol) were added to the reaction mixture. The reaction tube was sealed and heated in an oil bath at 120 °C. The progress of the reaction was monitored by thin-layer chromatography (TLC), and the reaction was quenched after approximately 30 minutes, upon completion. The reaction mixture was cooled to room temperature and extracted with ethyl acetate (3 × 10 mL) and water (30 mL). The combined organic layers were dried over anhydrous $MgSO_4$, filtered, and concentrated under reduced pressure. The crude product was analyzed by 1H-NMR spectroscopy without further purification.[1]

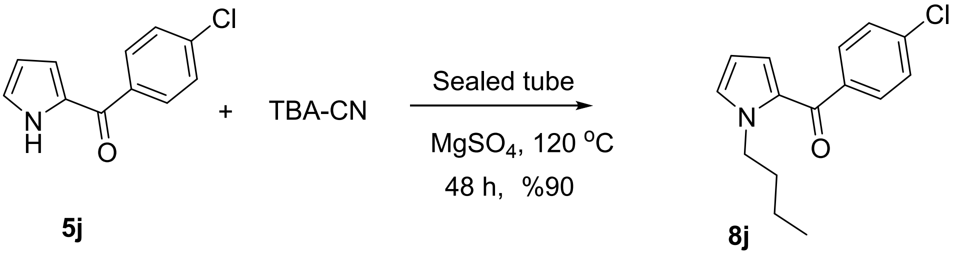

Scheme S2. Reaction of *N*H-Pyrrole derivative with TBACN

A solution of pyrrole derivative 5j (1 mmol) in anhydrous 1,4-dioxane (4 mL) was prepared. To this, anhydrous magnesium sulfate (50 mg) and TBACN (1 mmol) were added. The reaction tube was sealed and heated at 120 °C in an oil bath. Reaction progress was monitored by TLC, and the reaction was allowed to proceed for 48 hours before being quenched. After cooling to room temperature, the reaction mixture was extracted with ethyl acetate (3 × 10 mL) and water (30 mL). The combined organic extracts were dried over anhydrous $MgSO_4$, filtered, and concentrated under reduced pressure. The crude product was purified by silica gel column chromatography, using hexane/EtOAc (10:1) as the eluent, and characterized by NMR spectroscopy.

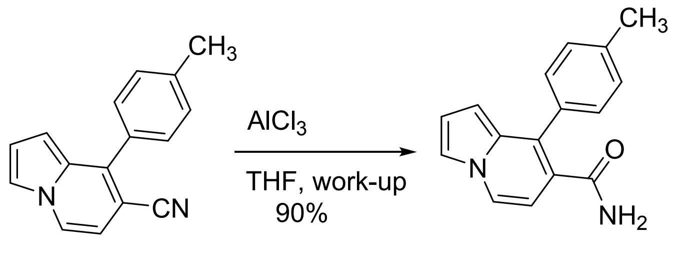

3f **9f**

Scheme S3. Conversion of CN group in acidic media.

Aluminum chloride ($AlCl_3$, 0.9 mmol) was suspended in dry THF (4 mL), and the reaction mixture was cooled to 0 °C. A solution of indolizine derivative 3f (1 mmol) in dry THF (2 mL) was added dropwise to the suspension under continuous stirring. The reaction was maintained in an ice bath and stirred for 2 hours. Subsequently, methanol (2 mL) was added to the reaction mixture in portions at 5-minute intervals, followed by the dropwise addition of water (2 mL) after an additional 10 minutes. Upon completion of the additions, the reaction was quenched and extracted with ethyl acetate in a 1 N NaOH aqueous solution. The combined organic layers were dried over anhydrous $MgSO_4$, filtered, and concentrated under reduced pressure. The crude product was purified by column chromatography on silica gel, eluting with hexane/ethyl acetate (5:1), and characterized by NMR spectroscopy.

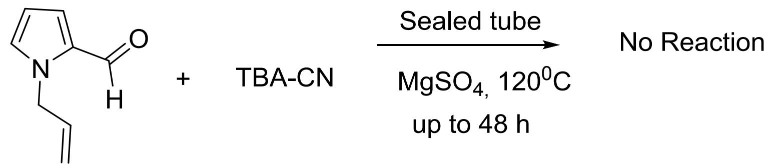

Scheme S4. Reaction of allyl-pyrrole derivative with TBACN

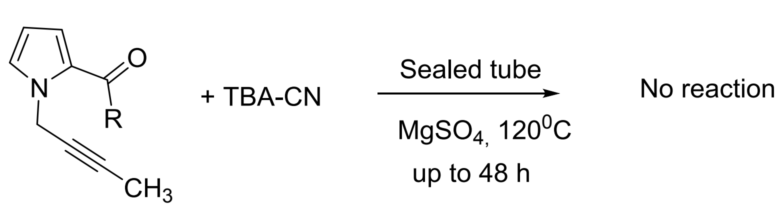

Scheme S5. Additional experiment 1

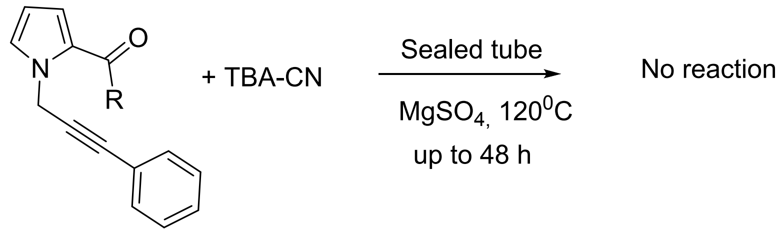

Scheme S6. Additional experiment 2.

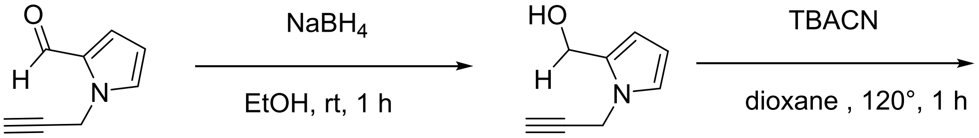

Scheme S7. Additional experiment 3.

1-(Prop-2-yn-1-yl)-1H-pyrrole-2-carbaldehyde (1 mmol) was dissolved in ethanol, and sodium borohydride ($NaBH_4$, 2 mmol) was added slowly at room temperature. The reaction mixture was stirred for 1 hour and monitored by TLC. Upon completion, the reaction was extracted with dichloromethane (DCM) and the organic layer was dried over magnesium sulfate. No further purification was performed at this stage. Subsequently, 1 mmol of the crude product was dissolved in dioxane. To this solution, TBACN (1 mmol) and additional magnesium sulfate were added. The reaction mixture was sealed in a tube and heated at 120 °C for 1 hour under stirring. Crude product was subjected for NMR analysis.

Table S1. Attempts to find out optimized reaction conditions.

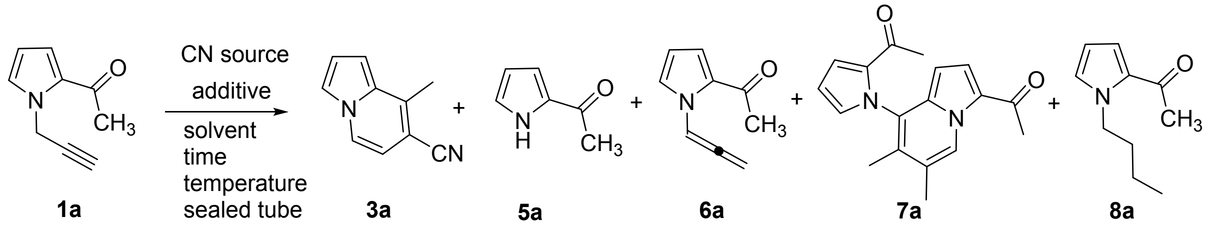

Entry	Solvent	Temperature[b]	$^{-}$CN/additive	Time	Product
1	EtOH	rt	TBACN	1 h	-[a]
2	EtOH	40	TBACN	1 h	-[a]
3	EtOH	70	TBACN	1 h	-[a]
4	EtOH	120	TBACN	1 h	-[a]
5	EtOH	120	TBACN	3 h	**1a** 5%; **3a** 15%; **5a** 80%; **7a** 5%
6	$CHCl_3$	120	TBACN	1 h	**1a** 90%; **3a** 10%

7	MeCN	rt	TBACN	1 h	-[a]
8	MeCN	50	TBACN	1 h	-[a]
9	MeCN	120	TBACN	3 h	**1a** %50; **3a** 50%
10	THF	rt	TBACN	1 h	-[a]
11	THF	120	TBACN	2 h	-[a]
12	THF	120	TBACN	6 h	**1a** 30%; **3a** %20; **5a** 50%
13	1,4-dioxane	rt	TBACN	1 h	-[a]
14	1,4-dioxane	40	TBACN	1 h	-[a]
15	1,4-dioxane	60	TBACN	1 h	-[a]
16	1,4-dioxane	80	TBACN	1 h	**1a** 20%; **3a** 40%; **5a** 20%; **6a** 20%
17	1,4-dioxane	120	TBACN	1 h	**3a** 50%; **5a** 40%; **6a** 10%,
**18***	**1,4-dioxane**	**120**	**TBACN** **$MgSO_4$**	**30 min**	**3a 75%; 5a 20%; 8a 5%**
19	1,4-dioxane	120	$MgSO_4$	30 min	-[a]
20	1,4-dioxane	120	TBACN $MgSO_4$	2 h	**3a** 75%; **5a** 15% ; **8a** 7%
21	1,4-dioxane	120	NaCN	3 h	-[a]

22	Ethylene glycol	Reflux	KCN	3 h	NR
23	1,4-dioxane	Reflux	CuCN	3 h	-[a]
24	Ethylene glycol	Reflux	KCN, TBAI	24 h	**3a** 55%, **7a** 25%
25	Ethylene glycol	Reflux	KCN, AcOH	24 h	**5a** 8%, **7a** 14%,

a: starting material was recovered. b: temperature represents the temperature of the oil bath. NR: no reaction.

Spectral data for the synthesized compounds

1-(1-(prop-2-yn-1-yl)-1H-pyrrol-2-yl)ethan-1-one (1a) [1]

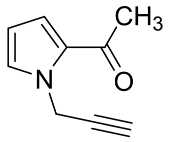

Orange-yellow liquid, Yield: 80%. ^{1}H NMR (400 MHz, $CDCl_3$) δ 7.12 (dd, J=2.4 Hz, 2.0 Hz, 1H, Ar-H), 6.92 (dd, J= 4.0, 2.0 Hz, 1H, Ar-H), 6.12 (dd, J=4.0, 2.4 Hz, 1H, Ar-H), 5.15 (d, J=2.6 Hz, 2H, $-CH_2$), 2.37 (s, 3H, CH_3), 2.36 (t, J=2.6 Hz, 1H, -CH). ^{13}C NMR (100 MHz, $CDCl_3$) δ 187.6, 129.0, 128.2, 119.5, 107.6, 77.2, 72.9, 37.8, 26.1.

1-(prop-2-yn-1-yl)-1H-pyrrole-2-carbaldehyde (1b) [1]

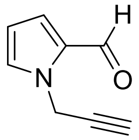

Yellow liquid, Yield: 89%. ^{1}H NMR (400 MHz, $CDCl_3$) δ 9.49 (bd, J=1.2 Hz, 1H, CHO), 7.20−7.19 (m, 1H, Ar-H), 6.90 (dd, J=4.0, 1.6 Hz, 1H, Ar-H), 6.22 (dd, J=4.0,2.4 Hz, 1H, Ar-H), 5.14 (d, J = 2.6 Hz, 2H, $-CH_2$), 2.39 (t, J = 2.6 Hz, 1H, -CH). ^{13}C NMR (100 MHz, $CDCl_3$) δ 179.5, 131.1, 130.4, 124.9, 110.1, 77.8, 74.4, 38.1.

2,2-dimethyl-1-(1-(prop-2-yn-1-yl)-1H-pyrrol-2-yl)propan-1-one (1c)

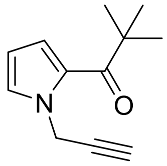

Clorless viscous liquid, Yield: 71%.^{1}H NMR (400 MHz, $CDCl_3$) δ = 7.43 (t, J=1.87 Hz, 1H, Ar-H), 6.71-6.70 (m, 1H, Ar-H), 6.67 (dd, J=1.87 Hz, J=3.00 Hz, 1H, Ar-H), 4.68 (d, J=2.60 Hz, 2H, $-CH_2-$), 2.49 (t, J=2.60 Hz, 1H, -CH), 1.32 (s, 9H, $-CH_3$). 13C NMR (100 MHz, $CDCl_3$) δ = 197.9, 128.1, 127.8, 119.3, 107.8, 78.7, 73.6, 43.8, 39.4, 28.8. HRMS: [M+Na] Calculated for $C_{12}H_{15}NONa$: 212.1051, Found: 212.1050.

Cyclopropyl(1-(prop-2-yn-1-yl)-1H-pyrrol-2-yl)methanone (1d)

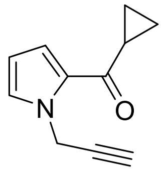

Gray viscous liquid, Yield: 82%.^{1}H NMR (400 MHz, $CDCl_3$) δ 7.19 (dd, J= 1.7 Hz, J= 2.6 Hz, 1H, Ar-H), 7.13 (dd, J= 1.7 Hz, J= 4.1 Hz, 1H, Ar-H), 6.22 (dd, J= 2.6 Hz, J= 4.1 Hz, 1H, Ar-H), 5.21 (d, J= 2.6 Hz, 2H, $-CH_2-$), 2.49-2.44 (m, 1H, -CH-), 2.42 (t, J= 2.6 Hz, 1H, -CH), 1.15-1.10 (m, 2H, $-CH_2-$), 0.92-0.86 (m, 2H, $-CH_2-$). ^{13}C NMR (100 MHz, $CDCl_3$) δ 190.6, 130.6, 128.9, 119.6, 108.7, 78.3, 73.8, 38.7, 17.8, 10.1. HRMS: $[M+H]^+$ Calculated for $C_{11}H_{12}NO$: 174.0913, Found: 174.0914.

Cyclohexyl(1-(prop-2-yn-1-yl)-1H-pyrrol-2-yl)methanone (1e)

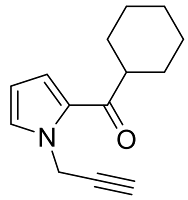

Dark Yellow viscous liquid, Yield: 95%. ^{1}H NMR (400 MHz, $CDCl_3$) δ = 7.18-7.17 (m, 1H, Ar-H), 6.99 (dd, *J*=1.7, 4.1 Hz, 1H, Ar-H), 6.16 (dd, *J*= 2.7, 4.1 Hz, 1H, Ar-H), 5.19 (d, *J*= 2.6 Hz, 2H, $-CH_2-$), 2.99 (tt, *J*= 3.2, 11.7 Hz, 1H, -CH), 2.41 (t, *J*=2.6 Hz, 1H, -CH), 1.83-1.78 (m, 4H, -CH), 1.71-1.66 (m, 1H, -CH), 1.55-1.45 (m, 2H, -CH), 1.35-1.23 (m, 3H, -CH). ^{13}C NMR (100 MHz, $CDCl_3$) δ = 195.1, 129.2, 129.1, 119.3, 108.4, 78.3, 73.9, 47.0, 38.9, 29.9, 29.8, 25.9. HRMS: $[M+H]^+$ Calculated for $C_{14}H_{18}NO$: 216.1388, Found: 216.1367.

(1-(prop-2-yn-1-yl)-1H-pyrrol-2-yl)(p-tolyl)methanone (1f)

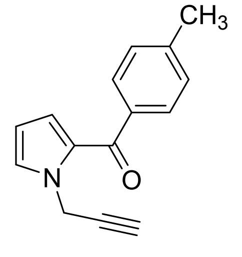

White solid, M.p: 66-68 °C, Yield: 90%. ^{1}H NMR (400 MHz, $CDCl_3$) *δ* = 7.76-7.70 (m, AA'BB'system, 2H, Ar-H), 7.29 (dd, *J*= 1.7, 2.6 Hz, 1H, Ar-H), 7.26-7.24 (m, AA'BB'system, 2H, Ar-H), 6.78 (dd, *J*= 1.7, 4.0 Hz, 1H, Ar-H), 6.22 (dd, *J*= 2.6, 4.0 Hz, 1H, Ar-H), 5.29 (d, *J*= 2.6 Hz, 2H, $-CH_2-$), 2.45 (t, *J*= 2.6 Hz, 1H, CH), 2.43 (s, 3H, $-CH_3$). ^{13}C NMR (100 MHz, $CDCl_3$) δ= 186.0, 142.2, 136.9, 129.9, 129.5, 129.4, 128.7, 123.1, 108.6, 78.3, 73.9, 38.6, 21.5. HRMS: $[M+H]^+$ Calculated for $C_{15}H_{14}NO^+$ 224.1075, Found 224.1041.

(4-(tert-butyl)phenyl)(1-(prop-2-yn-1-yl)-1H-pyrrol-2-yl)methanone (1g)

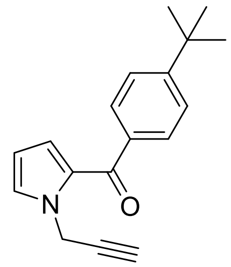

Light Brown viscous liquid, Yield: 95%. ^{1}H NMR (400 MHz, $CDCl_3$) *δ* 7.80-7.74 (m, AA'BB' system, 2H, Ar-H), 7.51-7.43 (m, AA'BB' system, 2H, Ar-H), 7.32-7.27 (m, 1H, Ar-H), 6.83-6.79 (m, 1H, Ar-H), 6.25-6.20 (m, 1H, Ar-H), 5.30 (d, J= 2.6 Hz, 2H, $-CH_2-$), 2.45 (t, J= 2.6 Hz, 1H, -CH), 1.36 (s, 9H, $-C(CH_3)_3$). ^{13}C NMR (100 MHz, $CDCl_3$) *δ* 185.9, 155.2, 136.8, 129.9, 129.4, 129.3, 125.0, 123.3, 108.6, 78.3, 73.9, 38.6, 35.0, 31.2. HRMS: $[M+H]^+$ Calculated for $C_{18}H_{20}NO$: 266.1539, Found: 266.1538.

(4-methoxyphenyl)(1-(prop-2-yn-1-yl)-1H-pyrrol-2-yl)methanone (1h)

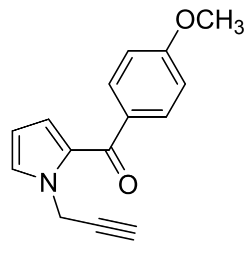

Brown solid, M.p: 82-83°C, Yield: 95%. 1H NMR (400 MHz, $CDCl_3$) δ = 7.86-7.82 (m, AA'BB' system, 2H, Ar-H), 7.27 (dd, J=1.7, 2.6 Hz, 1H, Ar-H), 6.97-6.93 (m, AA'BB' system, 2H, Ar-H), 6.76 (dd, J=1.7, 4.0 Hz, 1H, Ar-H), 6.22 (dd, J=2.6, 4.0 Hz, 1H, Ar-H), 5.27 (d, J=2.6 Hz, 2H, -NCH2), 3.87 (s, 3H, -OMe), 2.44 (t, J= 2.6 Hz, S4 1H, -CH). ^{13}C NMR (100 MHz, $CDCl_3$) δ= 185.1, 162.6, 132.1, 131.5, 129.9, 129.2, 122.6, 113.4, 108.6, 78.4, 73.9, 55.4, 38.5. HRMS: [M+H] Calculated for $C_{15}H_{14}NO_2$: 240.1025, Found: 240.1023.

(4-Fluorophenyl)(1-(prop-2-yn-1-yl)-1H-pyrrol-2-yl)methanone (1i)

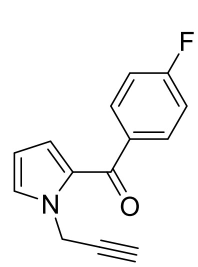

White solid, M.p: 87-89 °C, Yield: 92%. ^{1}H NMR (400 MHz, $CDCl_3$) *δ* = 7.86-7.82 (m, AA'BB'system, 2H, Ar-H), 7.31-7.30 (m, 1H, Ar-H), 7.16-7.10 (m, AA'BB'system, 2H, Ar-H), 6.76-6.74 (m, 1H, Ar-H), 6.246.22 (m, 1H, Ar-H), 5.28 (d, *J*= 2.5 Hz, 2H, $-CH_2-$), 2.46 (t, *J*= 2.5 Hz, 1H, -CH). ^{13}C NMR (100 MHz, $CDCl_3$) δ= 184.6, 164.9 (d, *J*= 252.4 Hz) , 135.8 (d, *J*= 3.0 Hz), 131.6 (d, *J*= 8.9 Hz), 129.9, 129.5, 123.3, 115.1 (d, *J*= 21.8 Hz), 108.9, 78.2, 74.0, 38.6. HRMS: $[M+H]^+$ Calculated for $C_{14}H_{11}FNO$: 228.0825, Found: 228.0811.

(4-chlorophenyl)(1-(prop-2-yn-1-yl)-1H-pyrrol-2-yl)methanone (1j)

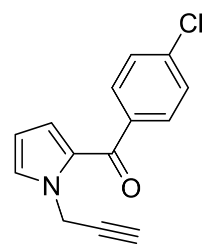

White solid, M.p: 89-91 °C, Yield: 92%. ^{1}H NMR (400 MHz, $CDCl_3$) δ = 7.77-7.74 (m, AA'BB'system, 2H, Ar-H), 7.44-7.41 (m, AA'BB'system, 2H, Ar-H), 7.31 (dd, *J*= 1.7, 2.6 Hz, 1H, Ar-H), 6.75 (dd, *J*= 1.7, 4.1 Hz, 1H, Ar-H), 6.23 (dd, *J*= 2.6, 4.1 Hz, 1H, Ar-H), 5.28 (d, *J*= 2.6 Hz, 2H, $-CH_2-$), 2.46 (t, *J*= 2.6 Hz, 1H, CH). ^{13}C NMR (100 MHz, $CDCl_3$) δ= 184.7, 137.9, 137.8, 130.6, 130.1, 129.4, 128.4, 123.5, 109.0, 78.1, 74.1, 38.7. HRMS: $[M+H]^+$ Calculated for $C_{14}H_{11}ClNO$: 244.0529, Found: 244.0501.

(4-bromophenyl)(1-(prop-2-yn-1-yl)-1H-pyrrol-2-yl)methanone (1k)

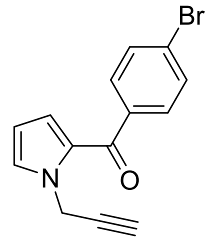

Light yellow solid, M.p: 129-130°C, Yield: 84%. ^{1}H NMR (400 MHz, $CDCl_3$) *δ* 7.70-7.66 (m, AA'BB' system, 2H, Ar-H), 7.61-7.57 (m, AA'BB' system, 2H, Ar-H), 7.31 (dd, J= 1.7 Hz, J=2.6 Hz, 1H, Ar-H), 6.75 (dd, J= 1.7 Hz, J= 4.1 Hz, 1H, Ar-H), 6.23 (dd, J= 2.6 Hz, J= 4.1 Hz, 1H, Ar-H), 5.28 (d, J= 2.6 Hz, 2H, $-CH_2-$), 2.46 (t, J= 2.6 Hz, 1H, -CH). ^{13}C NMR (100 MHz, $CDCl_3$) *δ* 184.8, 138.3, 131.3, 130.7, 130.1, 128.0, 126.4, 123.6, 109.0, 78.1, 74.1, 38.7. HRMS: $[M+H]^+$ Calculated for $C_{14}H_{11}BrNO$: 288.0018, Found: 288.0018.

(4-Nitrophenyl)(1-(prop-2-yn-1-yl)-1H-pyrrol-2-yl)methanone (1l)

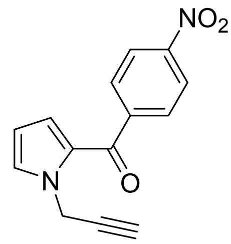

Light brown solid, M.p: 149-150°C, Yield: 96%. ^{1}H NMR (400 MHz, $CDCl_3$) *δ* = 8.32-8.29 (m, AA'BB' system, 2H, Ar-H), 7.94-7.91 (m, AA'BB' system, 2H, Ar-H), 7.38 (dd, *J*=1.7, 2.6 Hz, 1H, Ar-H), 6.74 (dd, *J*= 1.7, 4.1 Hz, 1H, Ar-H), 6.27 (dd, *J*=2.6, 4.1 Hz, 1H, Ar-H), 5.30 (d, *J*= 2.6 Hz, 2H, $-CH_2-$), 2.49 (t, *J*=2.6 Hz, 1H, -CH). ^{13}C NMR (100 MHz, $CDCl_3$) δ=183.7, 145.0, 131.1, 130.8, 129.9, 129.0, 124.4, 123.4, 109.5, 77.8, 74.4, 38.9. HRMS: $[M+H]^+$ Calculated for $C_{14}H_{11}N_2O_3$: 255.0770, Found 255.0748.

4-(1-(prop-2-yn-1-yl)-1H-pyrrole-2-carbonyl)benzonitrile (1m)

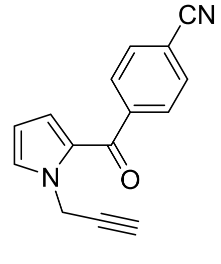

White solid, M.p: 149-150°C, Yield: 68%. ^{1}H NMR (400 MHz, $CDCl_3$) *δ* 7.88-7.85 (m, AA'BB' system, 2H, Ar-H), 7.77-7.74 (m, AA'BB' system, 2H, Ar-H), 7.36-7.33 (dd, J= 1.7 Hz, J= 2.6 Hz, 1H, Ar-H), 6.73 (dd, J= 1.7 Hz, J= 4.1 Hz, 1H, Ar-H), 6.26 (dd, J= 2.6 Hz, J= 4.1 Hz, 1H, Ar-H), 5.29 (d, J= 2.6 Hz, 2H, $-CH_2-$), 2.48 (t, J= 2.6 Hz, 1H, -CH). ^{13}C NMR (100 MHz, $CDCl_3$) *δ* 184.0, 143.3, 132.0, 130.9, 129.5, 129.0, 124.2, 118.1, 114.9, 109.4, 77.8, 74.4, 38.9. HRMS: $[M+H]^+$ Calculated for $C_{15}H_{11}N_2O$: 235.0866, Found: 235.0865.

(1-(prop-2-yn-1-yl)-1H-pyrrol-2-yl)(4-(trifluoromethyl)phenyl)methanone (1n)

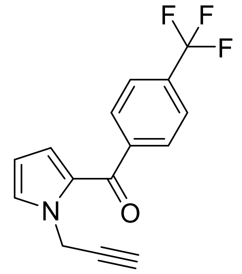

Light pink solid, M.p: 54-55°C, Yield: 66%. ^{1}H NMR (400 MHz, $CDCl_3$) δ 7.93-7.86 (m, AA'BB' system, 2H, Ar-H), 7.76-7.68 (m, AA'BB' system, 2H, Ar-H), 7.36-7.33 (m, 1H, Ar-H), 6.75 (dd, J= 1.7 Hz, J= 4.1 Hz, 1H, Ar-H), 6.25 (dd, J= 2.6 Hz, J= 4.1 Hz, 1H, Ar-H), 5.3 (d, J= 2.6 Hz, 2H, -CH_2-), 2.48 (t, J= 2.6 Hz, 1H, -CH). ^{13}C NMR (100 MHz, $CDCl_3$) δ 184.6, 142.7, 132.9 (q, J= 32.6 Hz), 130.6, 129.4, 129.3, 125.1 (q, J= 3.8 Hz), 124.2, 122.4, 78.0, 74.3, 38.8. HRMS: $[M+H]^+$ Calculated for $C_{15}H_{10}F_3NO$: 278.0787, Found: 278.0786.

Phenyl(1-(prop-2-yn-1-yl)-1H-pyrrol-2-yl)methanone (1o) [1]

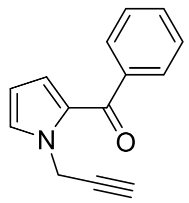

Light Brown viscous liquid, Yield: 89%. ^{1}H NMR (400 MHz, $CDCl_3$) δ = 7.83-7.79 (m, 2H, Ar-H), 7.56-7.52 (m, 1H, Ar-H), 7.47-7.42 (m, 2H, Ar-H), 7.31 (dd, J=1.7, 2.6 Hz, 1H, Ar-H), 6.78 (dd, J=1.7, J=4.1 Hz, 1H, Ar-H), 6.23 (dd, J=2.6,4.1 Hz, 1H, Ar-H), 5.31 (d, J=2.6 Hz, 2H, -CH_2-), 2.46 (t, J=2.6 Hz, 1H, Ar-H).^{13}C NMR (100 MHz, $CDCl_3$) δ= 186.2, 139.6, 131.6, 129.8, 129.2, 128.1, 123.6, 108.8, 78.3, 74.0, 38.7.

[1,1'-biphenyl]-4-yl(1-(prop-2-yn-1-yl)-1H-pyrrol-2-yl)methanone (1p)

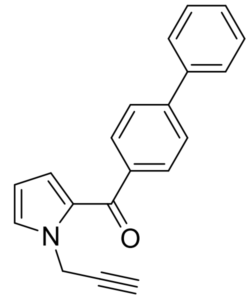

Light gray solid, M.p: 101-102°C, Yield: 88%. ^{1}H NMR (400 MHz, $CDCl_3$) δ = 7.93-7.89 (m, AA'BB' system, 2H, Ar-H), 7.71-7.67 (m, AA'BB' system, 2H, Ar-H), 7.67-7.63 (m, 2H, Ar-H), 7.51-7.45 (m, 2H, Ar-H), 7.43-7.38 (m, 1H, Ar-H), 7.33 (dd, J= 1.7 Hz, J= 2.6 Hz, 1H, Ar-H), 6.86 (dd, J= 1.7 Hz, J= 4.0 Hz, 1H, Ar-H), 6.26 (d, J= 2.6 Hz, J= 4.0 Hz, 1H, Ar-H), 5.33 (d, J= 2.6 Hz, 2H, -CH_2-), 2.48 (t, J= 2.6 Hz, 1H, -CH). ^{13}C NMR (100 MHz, $CDCl_3$) δ 185.7, 144.4, 140.2, 138.3, 129.8, 129.7, 128.9, 128.0, 127.2, 126.8, 123.4, 108.8, 78.3, 74.0, 38.7. HRMS: $[M+H]^+$ Calculated for $C_{20}H_{16}NO$: 286.1225, Found: 286.1226.

Naphthalen-2-yl(1-(prop-2-yn-1-yl)-1H-pyrrol-2-yl)methanone (1r)

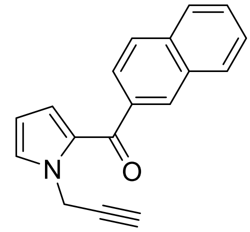

Yellow solid, M.p: 126-128°C, Yield: 90%. ^{1}H NMR (400 MHz, $CDCl_3$) δ =8.35 (bs, 1H, Ar-H), 7.95-7.89 (m, 4H, Ar-H), 7.61-7.53 (m, 2H, Ar-H), 7.34 (dd, *J*=1.8, 2.5 Hz, 1H, Ar-H), 6.87 (dd, *J*=1.8, 4.1 Hz, 1H, Ar-H), 6.27 (dd, *J*=2.5, 4.1 Hz, 1H, Ar-H), 5.36 (d, *J*=2.6 Hz, 2H, -CH_2-), 2.50 (t, *J*=2.6 Hz, 1H, -CH). ^{13}C NMR (100 MHz, $CDCl_3$) δ= 186.1, 136.8, 134.9, 132.3, 130.4, 130.0, 129.8, 129.2, 128.1, 127.9, 127.8, 126.7, 125.6, 123.7, 108.9, 78.4, 74.2, 38.7. HRMS: [M+Na] Calculated for $C_{18}H_{13}NONa$: 282.0889, Found: 282.0894.

Furan-2-yl(1-(prop-2-yn-1-yl)-1H-pyrrol-2-yl)methanone (1s)

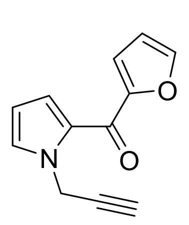

Yellow viscous liquid, Yield: 62%. ^{1}H NMR (400 MHz, $CDCl_3$) δ = 7.62 (dd, *J*=0.8, 1.7 Hz, 1H, Ar-H), 7.36 (dd, *J*=1.7, 4.1 Hz, 1H, Ar-H), 7.29-7.28 (m, 1H, Ar-H), 7.25 (dd, *J*=0.8, 3.5 Hz, 1H, Ar-H), 6.55 (dd, *J*=1.7, 3.5 Hz, 1H, Ar-H), 6.27 (dd, *J*=2.6, 4.1 Hz, 1H, Ar-H), 5.27 (d, *J*=2.6 Hz, 2H, $-CH_2-$), 2.44 (t, *J*=2.6 Hz, 1H, -CH). ^{13}C NMR (100 MHz, $CDCl_3$) δ= 171.9, 153.1, 145.8, 129.8, 128.6, 121.8, 117.9, 111.9, 109.2, 78.2, 74.0, 38.8. HRMS: [M+H] Calculated for $C_{12}H_{10}NO_2$: 200.0712, Found: 200.0719.

(1-(prop-2-yn-1-yl)-1H-pyrrol-2-yl)(thiophen-2-yl)methanone (1t)

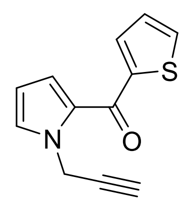

Dark Brown viscous liquid, Yield: 74%. ^{1}H NMR (400 MHz, $CDCl_3$) δ = 7.75 (dd, *J*=1.2, 3.8 Hz, 1H, Ar-H), 7.60 (dd, *J*=1.2, 5.0 Hz, 1H, Ar-H), 7.27 (dd, *J*=1.7, 2.6 Hz, 1H, Ar-H), 7.14 (dd, *J*=3.8, 5.0 Hz, 1H, Ar-H), 7.08 (dd, *J*=1.7, 4.1 Hz, 1H, Ar-H), 6.24 (dd, *J*=2.6, 4.1 Hz, 1H, Ar-H), 5.24 (d, *J*=2.6 Hz, 2H, CH_2-), 2.44 (t, *J*=2.6 Hz, 1H, -CH). ^{13}C NMR (100 MHz, $CDCl_3$) δ= 177.1, 144.4, 132.5, 132.3, 129.7, 129.4, 127.6, 121.7, 109.0, 78.2, 74.1, 38.5. HRMS: [M+H] Calculated for $C_{12}H_{10}NOS$: 216.0477, Found: 216.0482.

(2-methoxyphenyl)(1-(prop-2-yn-1-yl)-1H-pyrrol-2-yl)methanone (1u)

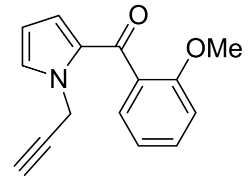

Dark Brown viscous liquid, Yield: 70%. ^{1}H NMR (400 MHz, $CDCl_3$) δ =7.34-7.22 (m, 3H), 6.92-6.88 (m, 2H), 6.48 (dd, J= 4.0, 1.7 Hz, 1H), 6.07 (dd, J= 4.0, 2.5 Hz, 1H), 5.29 (d, J= 2.6 Hz, 2H), 2.41 (t, J= 2.6 Hz, 1H). ^{13}C NMR (100 MHz, $CDCl_3$) δ = 185.7, 157.0, 131.1, 130.7, 129.9(2), 129.2, 124.2, 119.9, 111.5, 108.8, 78.1, 74.4, 55.8, 38.8.

1-(1-(but-2-yn-1-yl)-1H-pyrrol-2-yl)ethan-1-one (1v)

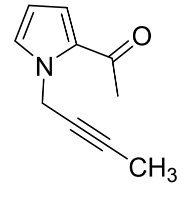

Dark Brown liquid, yield: 69%, ^{1}H NMR (400 MHz, $CDCl_3$) δ = 7.18-7.17 (m, 1H), 6.93-6.92 (m, 2H), 6.13-6.12 (m, 1H), 5.10 (q, J=2.6 Hz, 2H), 2.38 (s, 3H), 1.81 (t, J = 2.6 Hz, 3H). ^{13}C NMR (100 MHz, $CDCl_3$) δ = 188.3, 129.9, 129.1, 120.3, 108.1, 81.8, 73.6, 39.2, 29.6.

1-(3-phenylprop-2-yn-1-yl)-1H-pyrrole-2-carbaldehyde (1y)

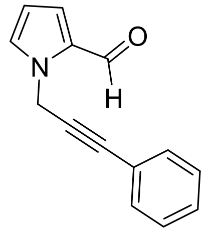

Dark Brown liquid, yield, 73%, ^{1}H NMR (400 MHz, $CDCl_3$) δ = 9.58 (d, J = 1.2Hz, 1H, CHO), 7.47-7.43 (m, 2H), 7.37-7.28 (m, 3H), 6.98-6.96 (m, 1H), 6.30-6.28 (m, 1H), 5.42 (s, 2H), ^{13}C NMR (100 MHz, $CDCl_3$) δ = 179.5, 131.8, 131.2, 130.4, 128.7, 128.3, 124.9, 122.2, 110.0, 86.1, 82.8, 39.0

(1-(prop-2-yn-1-yl)-1H-pyrrol-2-yl)methanol

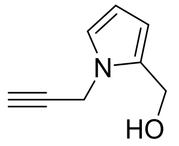

Yield: 99 % ^{1}H NMR (400 MHz, $CDCl_3$) δ 6.73 (dd, *J* = 2.8, 1.8 Hz, Ar-1H), 6.01 (qd, *J* = 3.6, 2.3 Hz, Ar-2H), 4.64 (d, *J* = 2.7 Hz, CH_2- 2H), 4.49 (s, CH_2 -2H), 2.31 (t, *J* = 2.6 Hz, CH -1H). ^{13}C NMR (101 MHz, $CDCl_3$) δ 131.3, 122.4, 109.7, 107.6, 78.6, 73.5, 56.5, 36.3.

Spectral data for indolizine derivatives (3a-u, 4j-k)

8-methylindolizine-7-carbonitrile (3a)

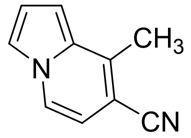

Green solid, Mp : 80°C, Yield: 75%. ^{1}H NMR (400 MHz, $CDCl_3$) δ = 7.81 (d, J=7.19 Hz, 1H, Ar-H), 7.42 (dd, J= 1,12 Hz, J= 2.61 Hz, 1H, Ar-H), 6.89 (dd, J= 2.61 Hz, J= 4.10 Hz, 1H, Ar-H), 6.72 (dd, J=1.12 Hz, J=4.10 Hz, 1H, Ar-H), 6.54 (d, J=7.19 Hz, 1H, Ar-H), 2.65 (s, 3H, $-CH_3$). ^{13}C NMR (100 MHz, $CDCl_3$): *δ* (ppm) = 136.8, 123.3, 118.5, 116.5, 115.2, 110.1, 103.4, 98.7, 16.7. HRMS: $[M+H]^+$ Calculated for $C_{10}H_9N_2$: 157.0774, Found: 157.0760.

indolizine-7-carbonitrile (3b)

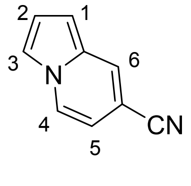

Brown viscous liquid, Yield: 70%. ^{1}H NMR (400 MHz, $CDCl_3$) δ = 7.93 (dt, $J_{4,3}$, $J_{4,6}$= 1.00 Hz, $J_{4,5}$= 7.23 Hz, 1H, H-4), 7.82 (bs, 1H, H-6), 7.46 (m, 1H, H-3), 6.93 (dd, $J_{2,3}$= 2.62 Hz, $J_{2,1}$=4.11 Hz, 1H, H-2), 6.73 (dd, $J_{1,3}$=0.95 Hz, $J_{1,2}$=4.11 Hz, 1H, H-1), 6.56 (dd, $J_{5,6}$=1.71 Hz, $J_{5,4}$=7.23 Hz, 1H, H-5). ^{13}C NMR (100 MHz, $CDCl_3$): *δ* (ppm) = 130.6, 126.6, 125.3, 119.1, 116.2, 116.1, 109.8, 104.8, 99.1. HRMS: $[M+H]^+$ Calculated for $C_9H_7N_2$: 143.0960, Found: 143.0965.

8-(tert-butyl)indolizine-7-carbonitrile (3c)

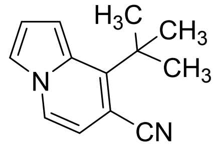

Light brown viscous liquid, Yield: 78%. ^{1}H NMR (400 MHz, $CDCl_3$) δ = 7.82 (td, J=0.75 Hz, J=7.13 Hz, 1H, Ar-H), 7.37 (quasi dd, J=0.75 Hz, J= 2.43 Hz, 1H, Ar-H), 7.03 (quasi dt, J=0.75 Hz, J=4.01 Hz, 1H, Ar-H), 6.86 (dd, J= 2.43 Hz, J= 4.01 Hz, 1H, Ar-H), 6.55 (d, J= 7.13 Hz, 1H, Ar-H), 1.72 (s, 9H, $-CH_3$). ^{13}C NMR (100 MHz, $CDCl_3$): *δ* (ppm) = 123.6, 121.2,

115.5, 114.5, 108.0, 38.0, 31.4. HRMS: $[M+H]^+$ Calculated for $C_{13}H_{15}N_2O$: 199.1230, Found: 199.1229.

8-cyclopropylindolizine-7-carbonitrile (3d)

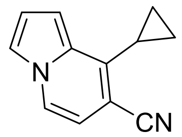

Brown viscous liquid, Yield: 74%. ^{1}H NMR (400 MHz, $CDCl_3$) δ = 7.80 (d, J= 7.2 Hz, 1H, Ar-H), 7.42-7.38 (m, 1H, Ar-H), 6.90-6.86 (m, 2H, Ar-H), 6.52 (d, J= 7.2 Hz, 1H, Ar-H), 2.25-2.18 (m, 1H, -CH-), 1.21-1.16 (m, 2H, $-CH_2-$), 1.15-1.11 (m, 2H, $-CH_2-$). ^{13}C NMR (100 MHz, $CDCl_3$): *δ* (ppm) = 141.0, 131.7, 123.5, 118.7, 116.1, 115.2, 110.7, 103.7, 99.3, 12.9. HRMS: $[M+H]^+$ Calculated for $C_{12}H_{11}N_2$: 183.0916, Found: 183.0919.

8-cyclohexylindolizine-7-carbonitrile (3e)

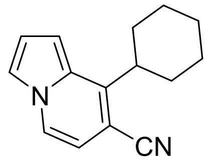

Dark green viscous liquid, Yield: 73%. ^{1}H NMR (400 MHz, $CDCl_3$) δ = 7.79 (dd, J= 1.14 Hz, J= 7.15 Hz, 1H, Ar-H), 7.41 (dd, J= J=1.14 Hz, J=2.59 Hz, 1H, Ar-H), 6.92 (dt, J= 1.14 Hz, J= 4.21 Hz, 1H, Ar-H), 6.87 (dd, J= 2.59 Hz, J= 4.21 Hz, 1H, Ar-H), 6.53 (d, J= 7.15 Hz, 1H, Ar-H), 3.25-3.17 (m, 1H), 2.20-2.13 (m, 2H), 1.94-1.80 (m, 4H), 1.49-1.40 (m, 3H). ^{13}C NMR (100 MHz, $CDCl_3$): *δ* (ppm) = 145.6, 123.3, 119.3, 116.2, 114.8, 111.2, 104.4, 30.7, 29.7, 26.8, 25.8. HRMS: $[M+H]^+$ Calculated for $C_{15}H_{17}N_2$: 225.1386, Found: 225.1383.

8-(p-tolyl)indolizine-7-carbonitrile (3f)

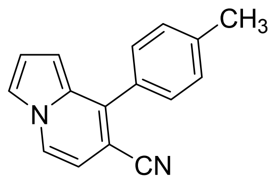

Dark green solid, Mp: 70°C, Yield: 78%. ^{1}H NMR (400 MHz, $CDCl_3$): *δ* (ppm) = 7.90 (dd, J= 0.99 Hz, J= 7.20 Hz, 1H, Ar-H), 7.55-7.52 (m, AA'BB' system, 2H, Ar-H), 7.49 (dd, J= 1.26 Hz, J= 2.62 Hz, 1H, Ar-H), 7.36-7.31 (m, AA'BB' system, 2H, Ar-H), 6.89 (dd, J= 2.62 Hz, J= 4.16 Hz, 1H, Ar-H), 6.68 (d, J= 7.20 Hz, 1H, Ar-H), 6.60 (dt, J= 1.26 Hz, J= 4.16 Hz, 1H, Ar-H), 2.45 (s, 3H, $-CH_3$). ^{13}C NMR (100 MHz, $CDCl_3$): *δ* (ppm) = 140.5, 139.5, 131.9, 131.3, 129.4, 128.9, 124.0, 119.0, 116.6, 115.8, 111.1, 105.6, 97.9, 21.4. HRMS: $[M+H]^+$ Calculated for $C_{16}H_{13}N_2$: 233.10912, Found: 233.10732.

8-(4-(tert-butyl)phenyl)indolizine-7-carbonitrile (3g)

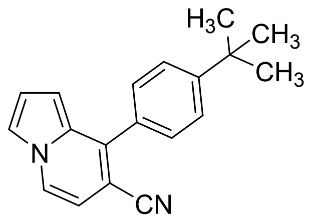

Light green solid, Mp: 78-79°C, Yield: 85%. ^{1}H NMR (400 MHz, $CDCl_3$): *δ* (ppm) = 7.90 (dd, J= 1.2 Hz, J= 7.2 Hz, 1H, Ar-H), 7.60-7.57 (m, AA'BB' system, 2H, Ar-H), 7.55-7.52 (m, AA'BB' system, 2H, Ar-H), 7.49 (dd, J= 1.2 Hz, J= 2.6 Hz, 1H, Ar-H), 6.89 (dd, J= 2.6 Hz, J= 4.2 Hz, 1H, Ar-H), 6.68 (d, J= 7.2 Hz, 1H, Ar-H), 6.65 (dt, J= 1.2 Hz, J= 4.2 Hz, 1H, Ar-H), 1.39 (s, 9H, $-C(CH_3)_3$).^{13}C NMR (100 MHz, $CDCl_3$): *δ* (ppm) = 131.8, 129.6, 128.8, 125.6, 123.9, 121.7, 119.1, 116.5, 115.8, 113.5, 111.2, 105.8, 100.2, 34.8, 31.3. HRMS: $[M+H]^+$ Calculated for $C_{19}H_{19}N_2$: 275.1542, Found: 275.1541.

8-(4-methoxyphenyl)indolizine-7-carbonitrile (3h)

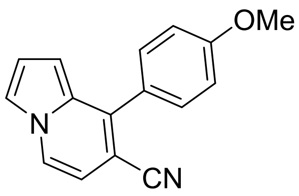

Green viscous liquid, Yield: 78%. ^{1}H NMR (400 MHz, $CDCl_3$): δ (ppm) = 7.89 (dd, J= 0.99 Hz, J= 7.19 Hz, 1H, Ar-H), 7.61-7.58 (m, AA'BB' system, 2H, Ar-H), 7.48 (dd, J= 1.26, J= 2.62 Hz, 1H, Ar-H), 7.07-7.03 (m, AA'BB' system, 2H, Ar-H), 6.89 (dd, J= 2.62 Hz, J= 4.16 Hz, 1H, Ar-H), 6.67 (d, J= 7.19 Hz, 1H, Ar-H), 6.61 (dt, J= 1.26 Hz, J= 4.16 Hz, 1H, Ar-H), 3.89 (s, 3H, OMe). ^{13}C NMR (100 MHz, $CDCl_3$): δ (ppm) = 160.5, 140.1, 130.4, 127.1, 123.9, 119.2, 116.6, 115.8, 114.1, 111.1, 105.6, 97.8, 55.3. HRMS: $[M+H]^+$ Calculated for $C_{16}H_{13}N_2O$: 249.10403, Found: 249.10224.

8-(4-fluorophenyl)indolizine-7-carbonitrile (3i)

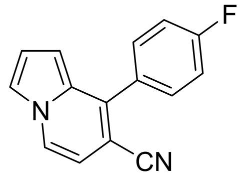

Green solid, Mp : 92°C, Yield: 70%. ^{1}H NMR (400 MHz, $CDCl_3$) δ = 7.93 (dd, J= 1.14 Hz, J=7.19 Hz, 1H, Ar-H), 7.65-7.60 (m, AA'BB' system, 2H, Ar-H), 7.51 (dd, J= 1.14 Hz, J= 2.61 Hz, 1H, Ar-H), 7.25-7.21 (m, AA'BB' system, 2H, Ar-H), 6.91 (dd, J= 2.61 Hz, J= 4.15 Hz, 1H, Ar-H), 6.69 (d, J= 7.19 Hz, 1H, Ar-H), 6.56 (dt, J= 1.14 Hz, J= 4.15 Hz, 1H, Ar-H). ^{13}C NMR (100 MHz, $CDCl_3$): δ (ppm) = 131.1, 131.0, 124.3, 116.8, 116.1, 115.8, 110.9, 105.5. HRMS: $[M+H]^+$ Calculated for $C_{15}H_{10}FN_2$: 237.0822, Found: 237.0823.

8-(4-chlorophenyl)indolizine-7-carbonitrile (3j)

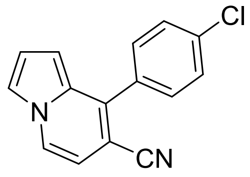

Yellow solid, Mp : 66°C, Yield: 72%. ^{1}H NMR (400 MHz, $CDCl_3$) δ = 7.93 (dd, J= 1.1 Hz, J= 7.2 Hz, 1H, Ar-H), 7.60-7.57 (m, AA'BB' system, 2H, Ar-H), 7.53-7.50 (m, 3H, Ar-H), 6.91 (dd, J= 2.6 Hz, J= 4.2 Hz, 1H, Ar-H), 6.69 (d, J= 7.2 Hz, 1H, Ar-H), 6.56 (dt, J= 1.1 Hz, J= 4.2 Hz, 1H, Ar-H). ^{13}C NMR (100 MHz, $CDCl_3$): δ (ppm) = 139.0, 135.6, 133.3, 130.4, 129.1, 128.4, 124.4, 118.6, 116.8, 116.1, 110.9, 105.5, 98.2. HRMS: $[M+H]^+$ Calculated for $C_{15}H_{10}ClN_2$: 253.0527, Found: 253.0524.

8-(4-bromophenyl)indolizine-7-carbonitrile (3k)

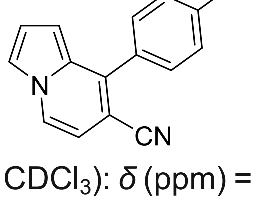

Yellow-green solid, Mp: 103-104°C, Yield: 74%. ^{1}H NMR (400 MHz, $CDCl_3$) δ = 7.94 (dd, J= 1.1 Hz, J= 7.2 Hz, 1H, Ar-H), 7.69-7.65 (m, AA'BB' system, 2H, Ar-H), 7.53-7.50 (m, 3H, Ar-H), 6.91 (dd, J= 2.6 Hz, J= 4.2 Hz, 1H, Ar-H), 6.69 (d, J= 7.2 Hz, 1H, Ar-H), 6.55 (dt, J= 1.1 Hz, J= 4.2 Hz, 1H, Ar-H). ^{13}C NMR (100 MHz, $CDCl_3$): δ (ppm) = 139.0, 133.7, 132.0, 130.7, 129.1, 124.5, 123.8, 118.6, 116.9, 116.1, 110.9, 105.5, 98.1. HRMS: $[M+H]^+$ Calculated for $C_{15}H_{10}BrN_2$: 297.0022, Found: 297.0023.

8-(4-nitrophenyl)indolizine-7-carbonitrile (3l)

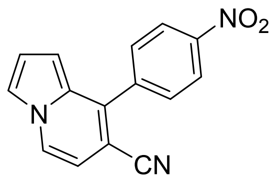

Black viscous liquid, Yield: 45%. ^{1}H NMR (400 MHz, $CDCl_3$) δ = 7.94 (dd, J= 1.08 Hz, J= 7.20 Hz, 1H, Ar-H), 7.60-7.57 (m, AA'BB' system, 2H, Ar-H), 7.53-7.50 (m, 3H, Ar-H), 6.91 (dd, J= 2.60 Hz, J= 4.18 Hz, 1H, Ar-H), 6.69 (d, J=7.20 Hz, 1H, Ar-H), 6.56 (dt, J= 1.08 Hz, J=4.18 Hz, 1H, Ar-H). ^{13}C NMR (100 MHz, $CDCl_3$): δ (ppm) = 139.0, 135.6, 133.3, 131.0, 130.4, 129.1, 124.5, 116.9, 116.1, 110.9, 105.5, 98.2. HRMS: $[M+Na]^+$ Calculated for $C_{15}H_9N_3O_2Na$: 286.0587, Found: 286.0599.

8-(4-cyanophenyl)indolizine-7-carbonitrile (3m)

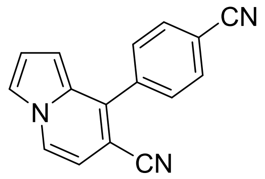

Brown viscous liquid, Yield: 52%.^{1}H NMR (400 MHz, $CDCl_3$): δ (ppm) = 7.99 (dd, J= 1.1 Hz, J= 7.2 Hz, 1H, Ar-H), 7.86-7.82 (m, AA'BB' system, 2H, Ar-H), 7.78-7.73 (m, AA'BB' system, 2H, Ar-H), 7.55 (dd, J= 1.1 Hz, J= 2.6 Hz, 1H, Ar-H), 6.94 (dd, J= 2.6 Hz, J= 4.2 Hz, 1H, Ar-H), 6.72 (dd, J= 7.2 Hz, 1H, Ar-H), 6.52 (dt, J= 1.1 Hz, J= 4.2 Hz, 1H, Ar-H). ^{13}C NMR (100 MHz, $CDCl_3$): δ (ppm) = 139.4, 138.0, 132.6, 130.4, 130.0, 125.1, 118.3, 118.1, 117.2, 116.5, 113.4, 110.8, 105.3, 95.5. HRMS: $[M+H]^+$ Calculated for $C_{16}H_{10}N_3$: 244.0869, Found: 244.0866.

8-(4-(trifluoromethyl)phenyl)indolizine-7-carbonitrile (3n)

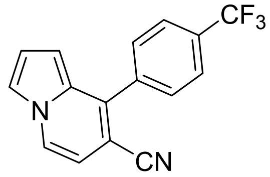

Dark green solid, Mp : 79-80°C, Yield: 58%. ^{1}H NMR (400 MHz, $CDCl_3$): δ (ppm) = 7.97 (dd, J= 1.2 Hz, J= 7.2 Hz, 1H, Ar-H), 7.83-7.78 (m, AA'BB' system, 2H, Ar-H), 7.78-7.74 (m, AA'BB' system, 2H, Ar-H), 7.54 (dd, J= 1.2 Hz, J= 2.6 Hz, 1H, Ar-H), 6.93 (dd, J= 2.6 Hz, J= 4.2 Hz, 1H, Ar-H), 6.72 (d, J= 7.2 Hz, 1H, Ar-H), 6.54 (dt, J= 1.2 Hz, J= 4.2 Hz, 1H, Ar-H). ^{13}C NMR (100 MHz, $CDCl_3$): δ (ppm) = 138.6, 138.5, 138.4, 130.8, 129.6, 125.8 (q, J= 3.8 Hz), 125.2, 124.8, 118.3, 117.0, 116.3, 110.9, 105.5, 98.4. HRMS: $[M+H]^+$ Calculated for $C_{16}H_{10}F_3N_2$: 287.0790, Found: 287.0788.

8-phenylindolizine-7-carbonitrile (3o)

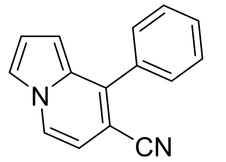

Yellow solid, Mp : 147-148°C, Yield: 80%. ^{1}H NMR (400 MHz, $CDCl_3$) δ = 7.92 (dd, J= 1.07 Hz, 7.20 Hz, 1H, Ar-H), 7.65-7.62 (m, 2H, Ar-H), 7.56-7.50 (m, 4H, Ar-H), 6.90 (dd, J= 2.61 Hz, J= 4.15 Hz, 1H, Ar-H), 6.69 (d, J=7.20 Hz, 1H, Ar-h), 6.59 (dt, J= 1.07 Hz, J= 4.15 Hz, 1H, Ar-H). ^{13}C NMR (100 MHz, $CDCl_3$): δ (ppm) = 140.3, 134.9, 131.2, 129.5, 129.1, 128.7, 124.2, 118.9, 116.7, 115.9, 111.1, 105.6, 98.1. HRMS: $[M+H]^+$ Calculated for $C_{15}H_{11}N_2$: 219.0916, Found: 219.0915.

8-([1,1'-biphenyl]-4-yl)indolizine-7-carbonitrile (3p)

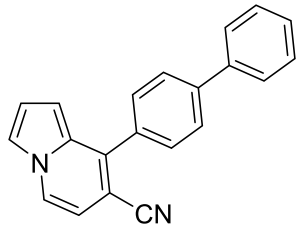

Dark yellow solid, Mp: 91°C , Yield: 72%. ^{1}H NMR (400 MHz, $CDCl_3$): δ (ppm) = 7.94 (dd, J= 1.2 Hz, J= 7.2 Hz, 1H, Ar-H), 7.77-7.71 (m, 4H, Ar-H), 7.69-7.66 (m, 2H, Ar-H), 7.52 (dd, J= 1.2 Hz, J= 2.6 Hz, 1H, Ar-H), 7.51-7.46 (m, 2H, Ar-H), 7.42-7.32 (m, 1H, Ar-H), 6.92 (dd, J=2.6 Hz, J= 4.1 Hz, 1H, Ar-H), 7.71 (d, J= 7.2 Hz, 1H, Ar-H), 6.67 (dt, J= 1.2 Hz, J= 4.1 Hz, 1H, Ar-H). ^{13}C NMR (100 MHz, $CDCl_3$): δ (ppm) = 142.3, 140.4, 140.0, 133.7, 129.5, 128.9, 128.8, 127.7, 127.4, 127.2, 124.2, 119.0, 116.7, 116.0, 111.1, 105.7, 98.0. HRMS: $[M+H]^+$ Calculated for $C_{21}H_{15}N_2$: 295.1229, Found: 295.1228.

8-(naphthalen-2-yl)indolizine-7-carbonitrile (3r)

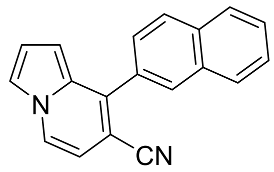

Black solid, Mp: 63°C, Yield: 83%. ^{1}H NMR (400 MHz, $CDCl_3$) δ =8.14 (d, J= 1.88 Hz, 1H, Ar-H), 8.00 (d, J= 8.39 Hz, 1H, Ar-H), 7.96 (dd, J=1.15 Hz, J= 7.21 Hz, 1H, Ar-H), 7.94-7.92 (m, 2H, Ar-H), 7.72 (dd, J=1.78 Hz, J= 8.45 Hz, 1H, Ar-H), 7.60-7.55 (m, 2H, Ar-H), 7.53 (dd, J= 1.15 Hz, J=2.62 Hz, 1H, Ar-H), 6.92 (dd, J= 2.62 Hz, J= 4.15 Hz, 1H, Ar-H), 6.74 (d, J= 7.21 Hz, 1H, Ar-H), 6.62 (d,t, J= 1.15 Hz, J= 4.15 Hz, 1H, Ar-H). ^{13}C NMR (100 MHz, $CDCl_3$): δ (ppm) = 140.3, 133.6, 133.1, 132.3, 131.4, 128.8, 128.5, 127.8, 127.0, 126.6, 126.4, 124.3, 118.9, 116.7, 116.0, 111.1, 105.7, 98.4. HRMS: $[M+H]^+$ Calculated for $C_{19}H_{13}N_2$: 269.1073, Found: 269.1072.

8-(furan-2-yl)indolizine-7-carbonitrile (3s)

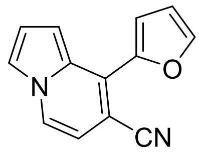

Light green solid, Mp : 164°C, Yield : 81%. ^{1}H NMR (400 MHz, $CDCl_3$): δ (ppm) = 7.88 (dd, J= 1.00 Hz, J= 7.15 Hz, 1H, Ar-H), 7.69 (dd, J= 0.71 Hz, 1.79 Hz, 1H, Ar-H), 7.48 (dd, J= 1.35 Hz, J= 2.63 Hz, 1H, Ar-H), 7.37 (dd, J= 0.71 Hz, J= 3.57 Hz, 1H, Ar-H), 7.24-7.22 (m, 1H, Ar-H), 6.96 (dd, J= 2.63 Hz, J=4.20 Hz, 1H, Ar-H), 6.66 -6.64 (m, 2H, Ar-H). ^{13}C NMR (100 MHz, $CDCl_3$): δ (ppm) = 145.3, 143.7, 139.0, 133.6, 128.2, 124.0, 119.6, 116.6, 116.1, 113.6, 112.0, 111.2, 106.7. HRMS: $[M+H]^+$ Calculated for $C_{13}H_9N_2O$: 209.07251, Found: 209.07094.

8-(thiophen-2-yl)indolizine-7-carbonitrile (3t)

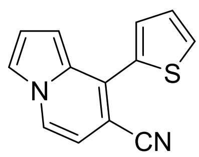

Dark green solid, Mp : 72°C, Yield : 85%. ^{1}H NMR (400 MHz, $CDCl_3$): δ (ppm) = 7.90 (dd, J= 0.59 Hz, J= 7.21 Hz, 1H, Ar-H), 7.62 (dd, J= 0.71 Hz, J= 3.26 Hz, 1H, Ar-H), 7.55 (dd, J= 1.25 Hz, J= 5.16 Hz, 1H, Ar-H), 7.49 (dd, J= 1.25 Hz, J= 2.54 Hz, 1H, Ar-H), 7.22 (dd, J= 3.81 H,z J= 5.16 Hz, 1H, Ar-H), 6.97-6.92 (m, 2H, Ar-H), 6.67 (d, J= 7.21 Hz, 1H, Ar-H). ^{13}C NMR (100 MHz, $CDCl_3$): δ (ppm) = 134.9, 132.8, 129.6, 127.8, 127.5, 124.3, 119.0, 116.9, 116.1, 111.1, 106.1, 98.3. HRMS: $[M+H]^+$ Calculated for $C_{13}H_9N_2S$: 225.04985, Found: 225.04810.

8-(2-methoxyphenyl)indolizine-7-carbonitrile (3u)

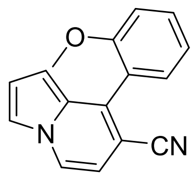

Light green solid, Mp : 171 °C, Yield: 54%. ^{1}H NMR (400 MHz, $CDCl_3$): δ (ppm) = 7.85 (dd, J= 7.1, 1.0 Hz, 1H), 7.42-7.37 (m, 2H), 7.33-7.30 (m, 1H), 7.03-6.99 (m, 2H), 6.93-6.89 (m, 1H), 6.79 (dd, J= 4.2, 2.5 Hz, 1H), 6.61 (d, J= 7.2 Hz, 1H). ^{13}C NMR (100 MHz, $CDCl_3$): δ (ppm) = 155.2, 129.9, 129.8, 128.2, 123.2, 123.1, 119.2, 118.8, 117.9, 115.1, 114.8, 110.9, 109.8, 104.0, 98.6, 54.3.

(1-butyl-1*H*-pyrrol-2-yl)(4-chlorophenyl) methanone (7j)

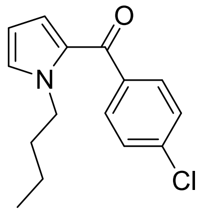

Dark red viscous liquid, Yield: 10%,^{1}H NMR (400 MHz, $CDCl_3$) δ = 8.32-8.28 (m, AA'BB' system, 2H, Ar-H), 7.92-7.89 (m, AA'BB' system, 2H, Ar-H), 7.05 (dd, J= 1.66 Hz, J= 2.45 Hz, 1H, Ar-H), 6.67 (dd, J= 1.66 Hz, J= 4.14 Hz, 1H, Ar-H), 6.19 (dd, J=2.45 Hz, J= 4.14 Hz, 1H, Ar-H), 4.40 (t, J= 7.33 Hz, 2H, $-CH_2-$), 1.85-1.77 (m, 2H, $-CH_2-$), 0.96 (t, J= 7.38 Hz, 3H, $-CH_3$). ^{13}C NMR (100 MHz, $CDCl_3$): δ (ppm) = 183.5, 145.7, 132.0, 129.8, 129.0, 124.2, 123.3, 108.8, 49.6, 33.7, 19.8, 13.7. HRMS: $[M+H]^+$ Calculated for $C_{15}H_{17}ClNO$: 262.0999, Found: 262.0998.

8-(4-chlorophenyl)indolizine-6-carbonitrile (4j)

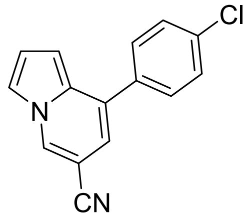

Dark green solid, Mp : 123°C, Yield : 5%,^{1}H NMR (400 MHz, $CDCl_3$) δ = 8.37 (t, J= 1.20 Hz, 1H, Ar-H), 7.59-756 (m, AA'BB' system, 2H, Ar-H), 7.49-7.45 (m, 3H, Ar-H), 6.96 (dd, J= 2.80 Hz, J= 4.00 Hz, 1H, Ar-H), 6.68 (d, J= 1.20 Hz, 1H, Ar-H), 6.63 (dt, J= 1.20 Hz, J= 4.00 Hz, 1H, Ar-H). ^{13}C NMR (100 MHz, $CDCl_3$): δ (ppm) = 135.6, 134.8, 133.0, 131.1, 129.4, 129.1, 117.7, 116.9, 115.4, 115.0, 102.5, 96.5. HRMS: $[M+H]^+$ Calculated for $C_{15}H_{10}ClN_2$: 253.0533, Found: 253.0530.

8-(4-bromophenyl)indolizine-6-carbonitrile (4k)

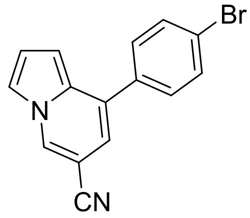

Dark green solid, Mp : 133°C, Yield : 7%, ^{1}H NMR (400 MHz, $CDCl_3$) δ = 8.40 (t, J= 1.2 Hz, 1H, Ar-H), 7.82-7.78 (m, AA'BB' system, 2H, Ar-H), 7.77-7.74 (m, AA'BB' system, 2H, Ar-H), 7.51 (dd, J= 1.2 Hz, J= 2.8 Hz, 1H, Ar-H), 6.99 (dd, J= 2.8 Hz, J= 4.0 Hz, 1H, Ar-H), 6.73 (d, J= 1.2 Hz, 1H, Ar-H), 6.62 (dt, J= 1.2 Hz, J= 4.0 Hz, 1H, Ar-H). ^{13}C NMR (100 MHz, $CDCl_3$): δ (ppm) = 145.0, 141.8, 137.8, 132.7, 132.2, 131.8, 128.8, 118.4, 117.2, 117.0, 115.8, 115.7, 102.4, 96.5. HRMS: $[M+H]^+$ Calculated for $C_{15}H_{10}BrN_2$: 297.0027, Found: 297.0028.

8-(p-tolyl)indolizine-7-carboxamide (9f)

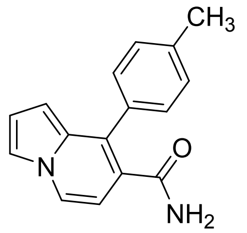

Dark green solid, m.p.: 201 ^{o}C, ^{1}H NMR (400 MHz, $CDCl_3$) δ = 7.93 (dd, J= 1.2 Hz, J= 7.2 Hz, 1H, Ar-H), 7.42 (dd, J= 1.2 Hz, J= 2.6 Hz, 1H, Ar-H), 7.38-7.36 (m, AA'BB' system, 2H, Ar-H), 7.33-7.31 (m, AA'BB' system, 2H, Ar-H), 7.16 (d, J= 7.2 Hz, 1H, Ar-H), 6.78 (dd, J= 2.6 Hz, J=4.0 Hz, 1H, Ar-H), 6.19 (dd, J= 1.2 Hz, J= 4.0 Hz, 1H, Ar-H), 5.46 (bs, 1H, -NH), 5.19 (bs, 1H, -NH), 2.45 (s, 3H, $-CH_3$). ^{13}C NMR (100 MHz, $CDCl_3$): δ (ppm) = 169.1, 138.8, 133.5, 132.5, 129.9, 129.0, 123.9, 120.3, 114.9, 114.7, 111.0, 104.1, 21.3. HRMS: $[M+H]^+$ Calculated for $C_{16}H_{15}N_2O$: 251.1184, Found: 251.1190.

Copies ^{1}H NMR and ^{13}C NMR spectra

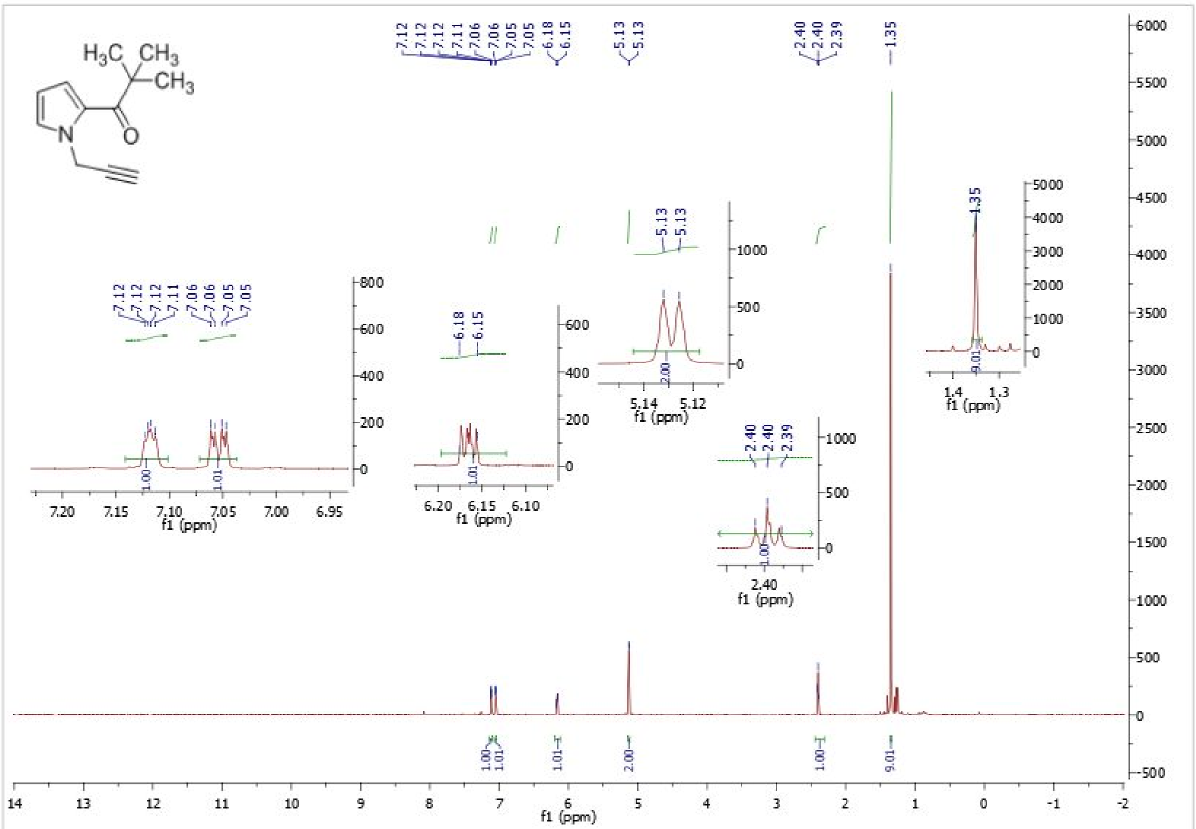

Figure S1.

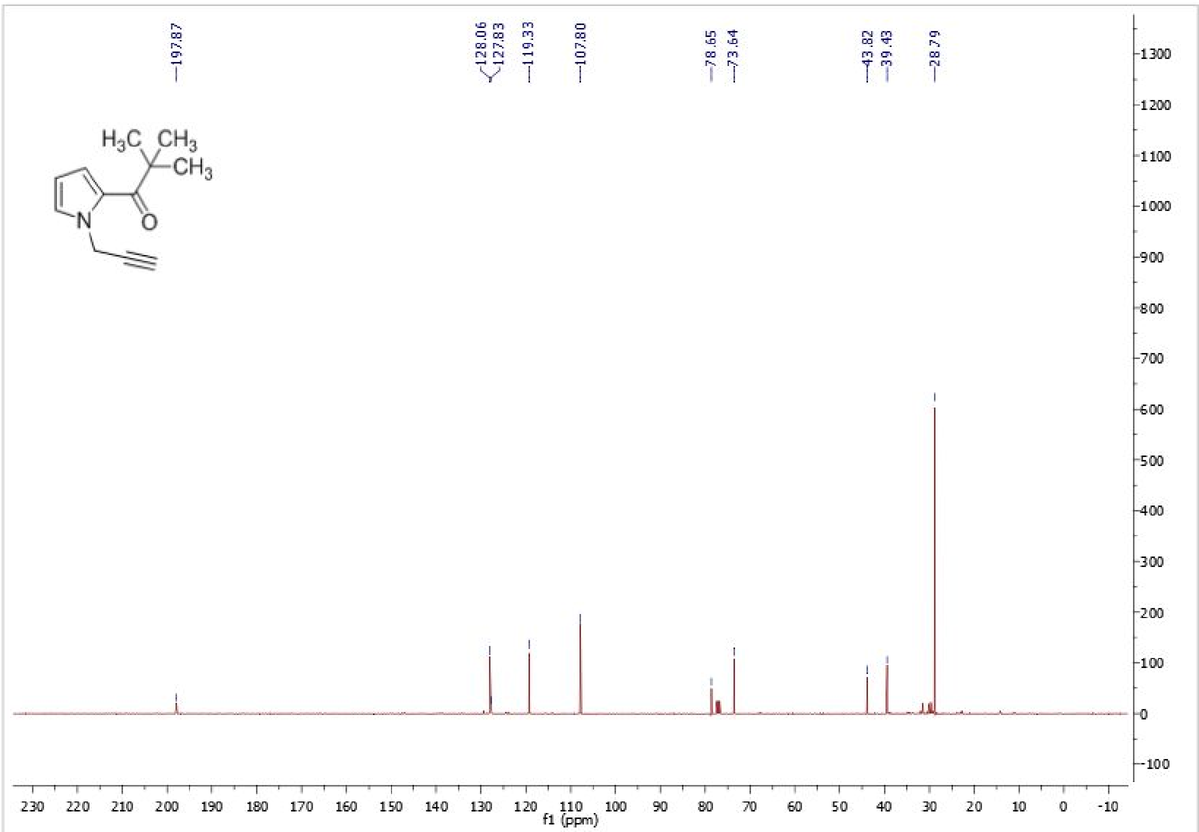

Figure S2.

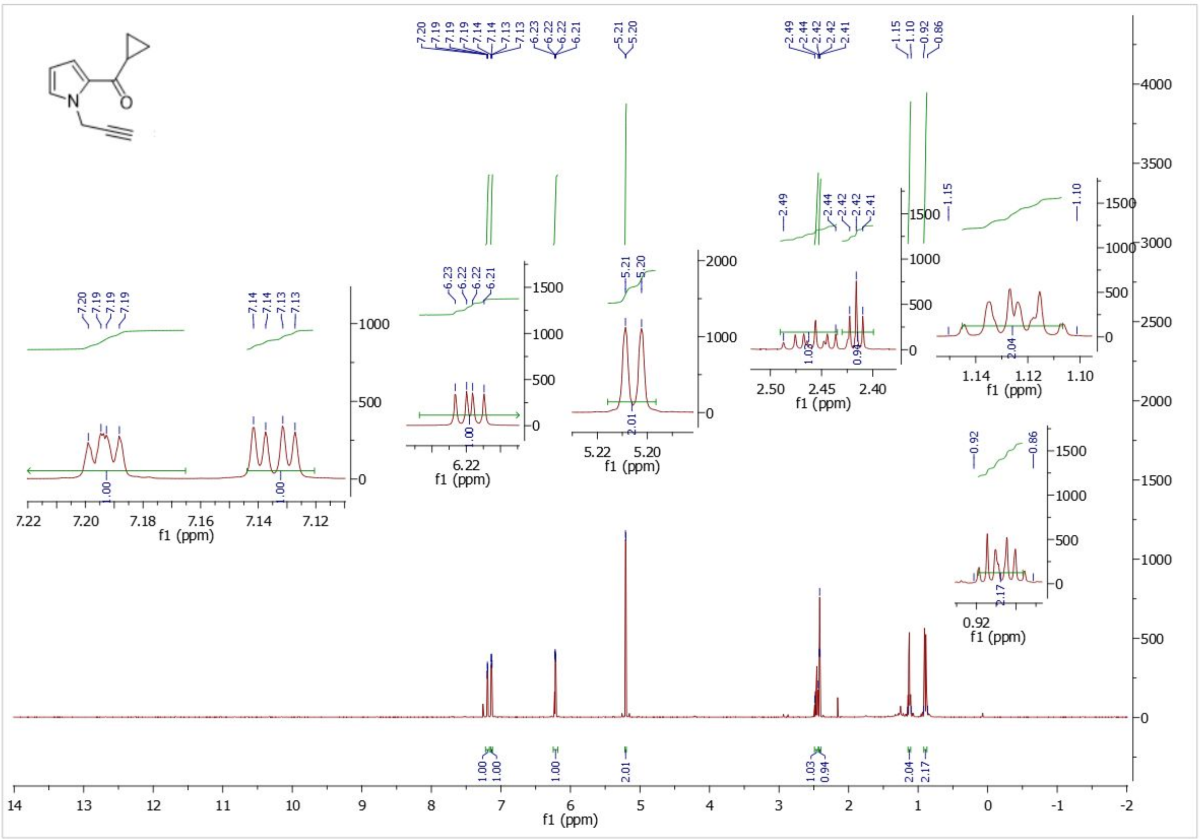

Figure S3.

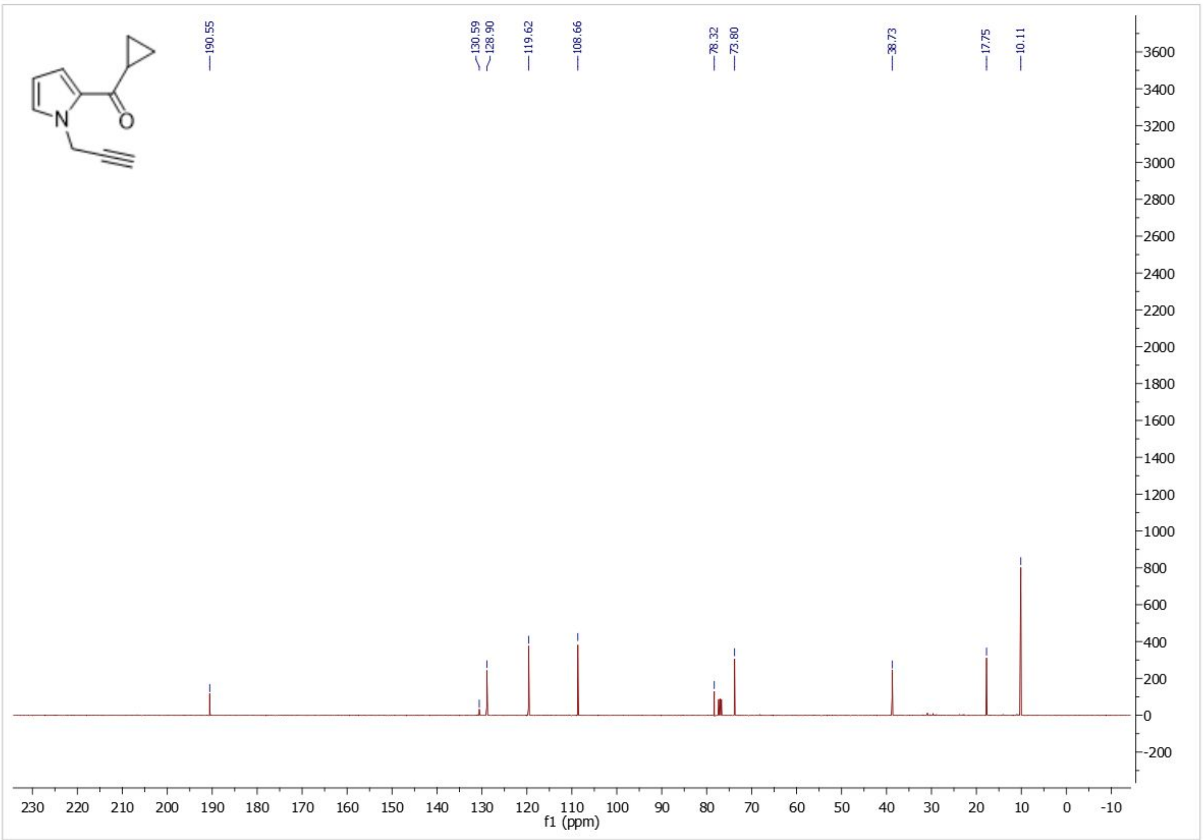

Figure S4.

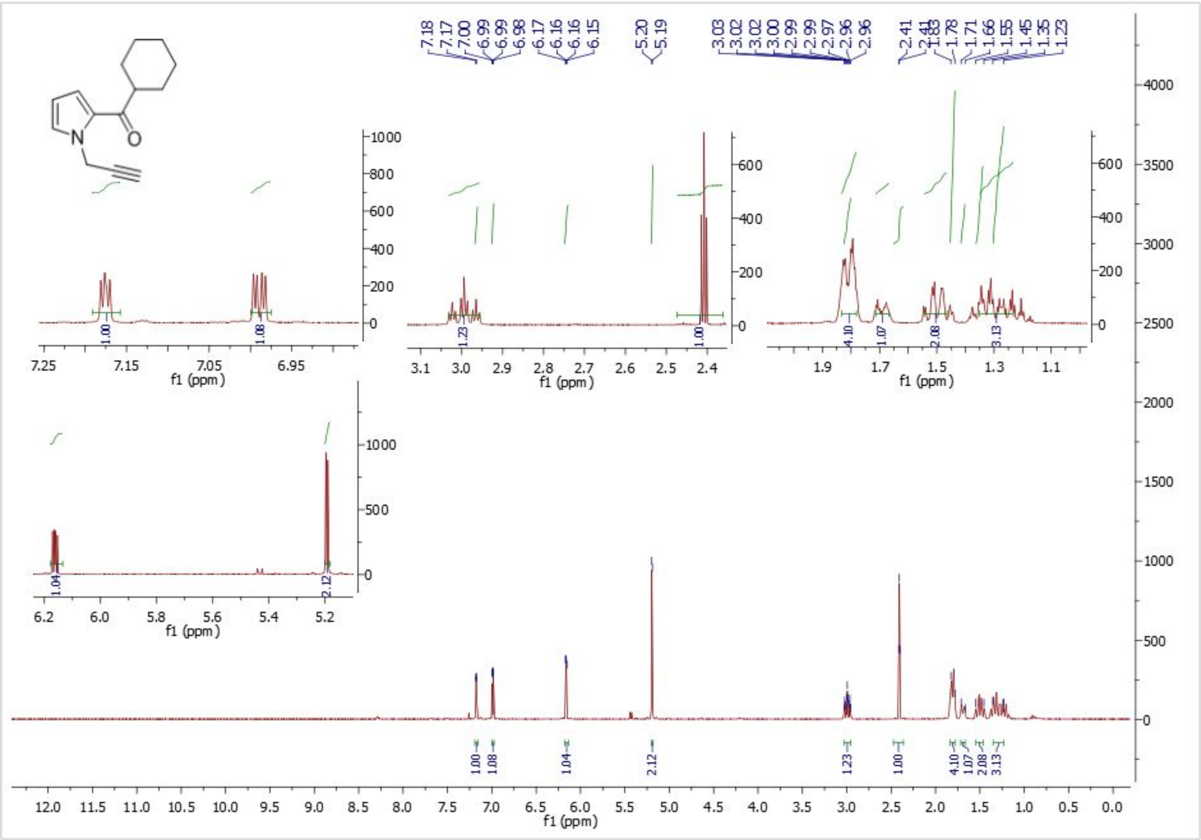

Figure S5.

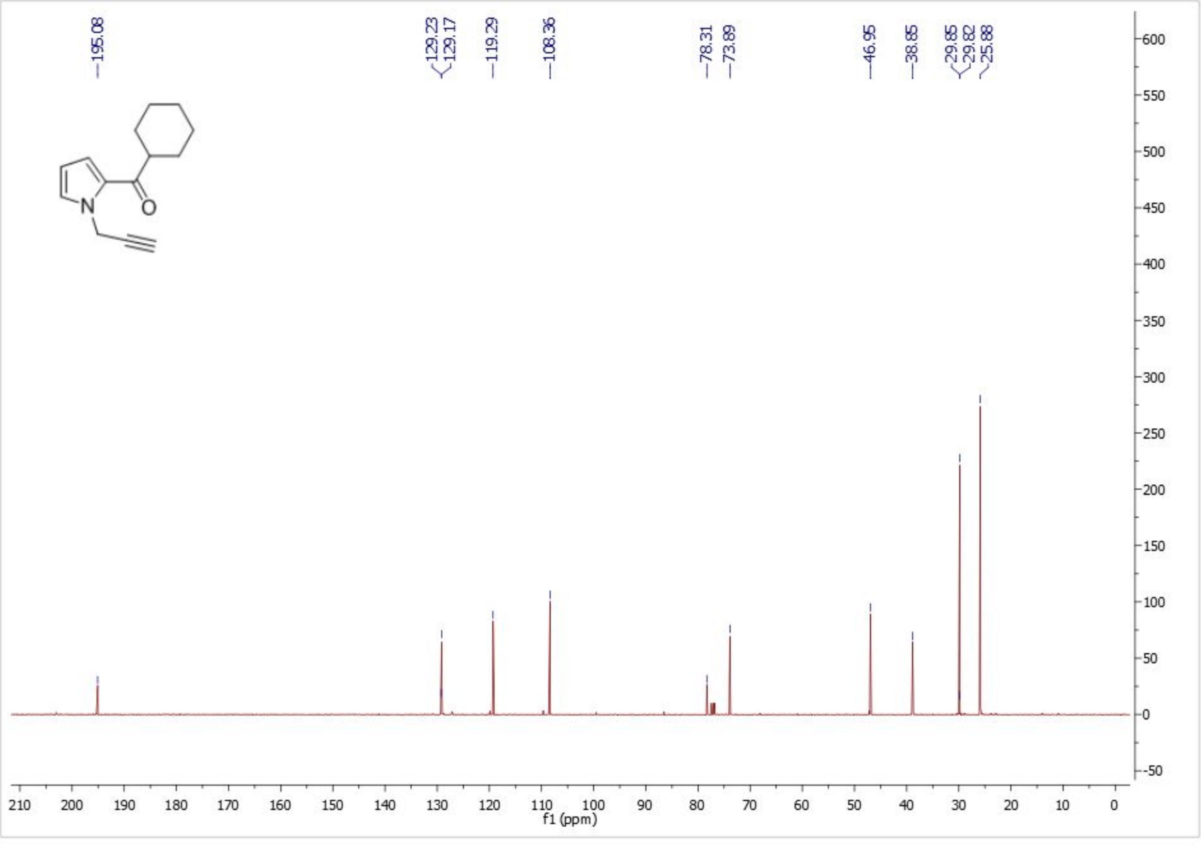

Figure S6.

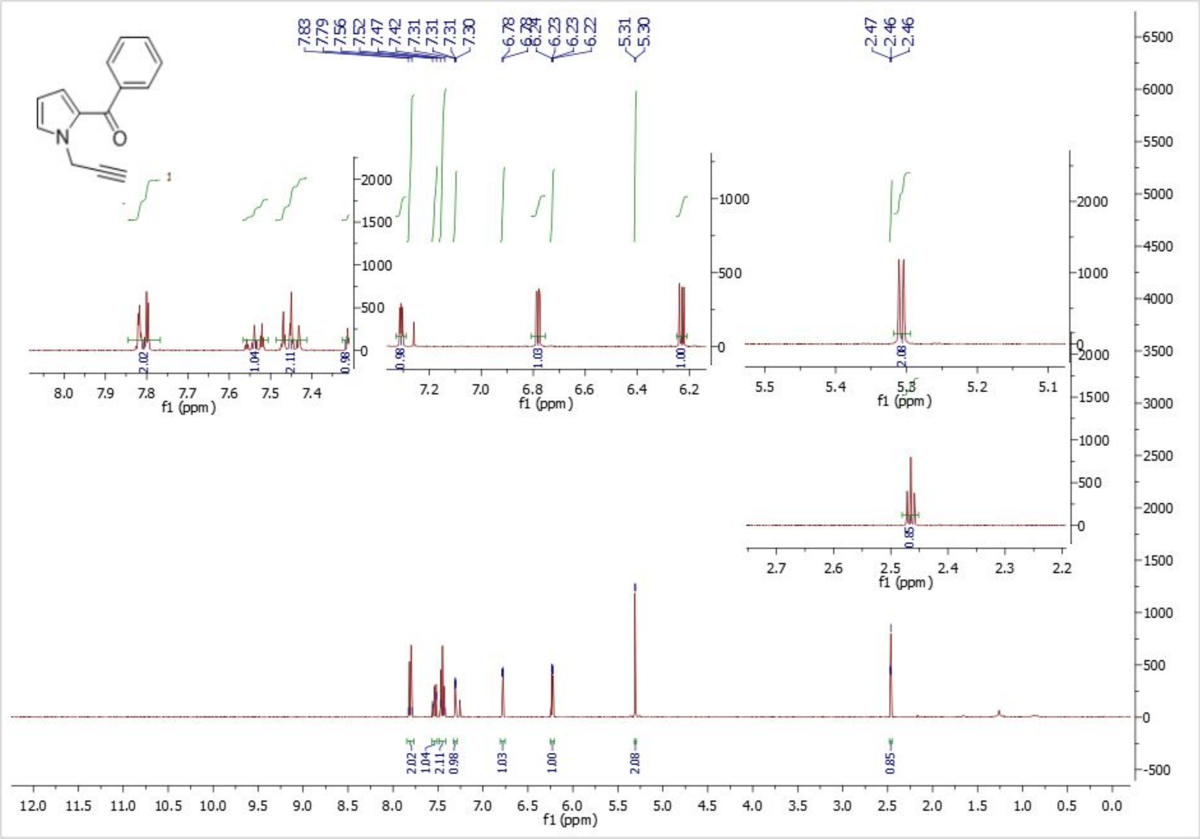

Figure S7.

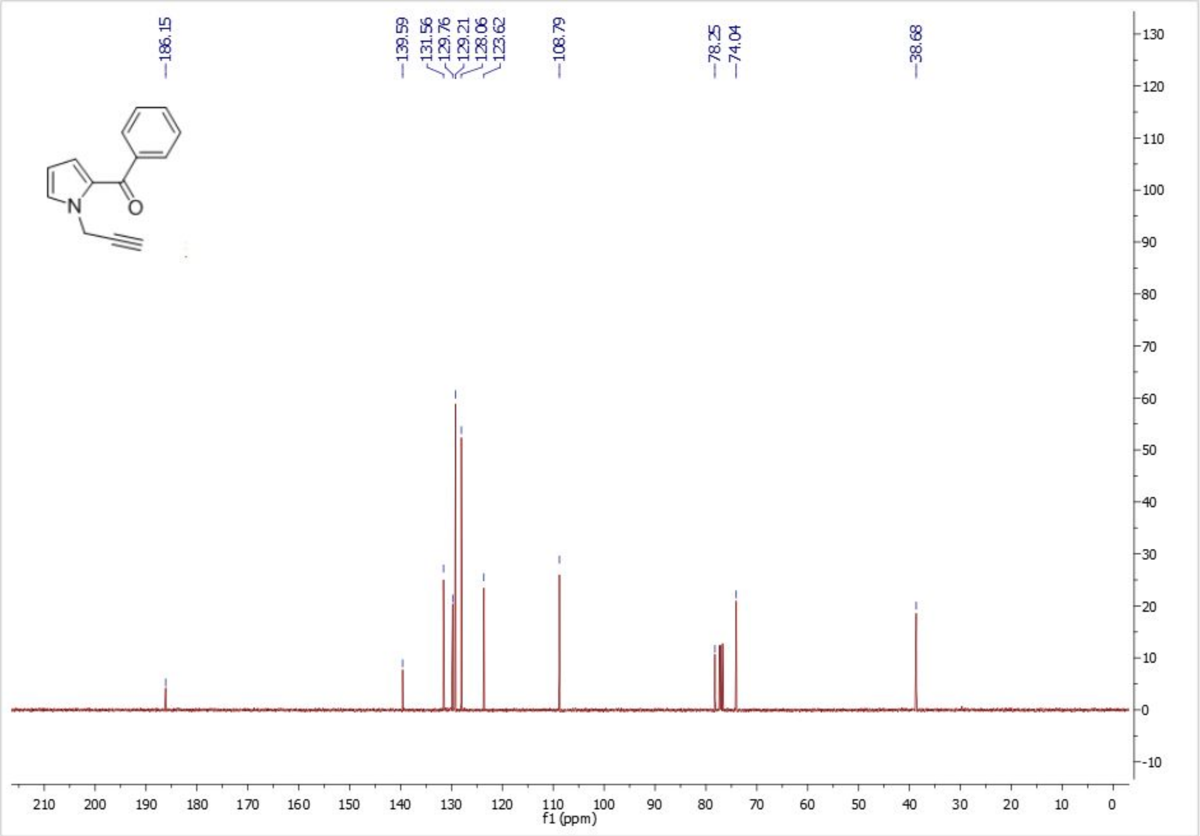

Figure S8.

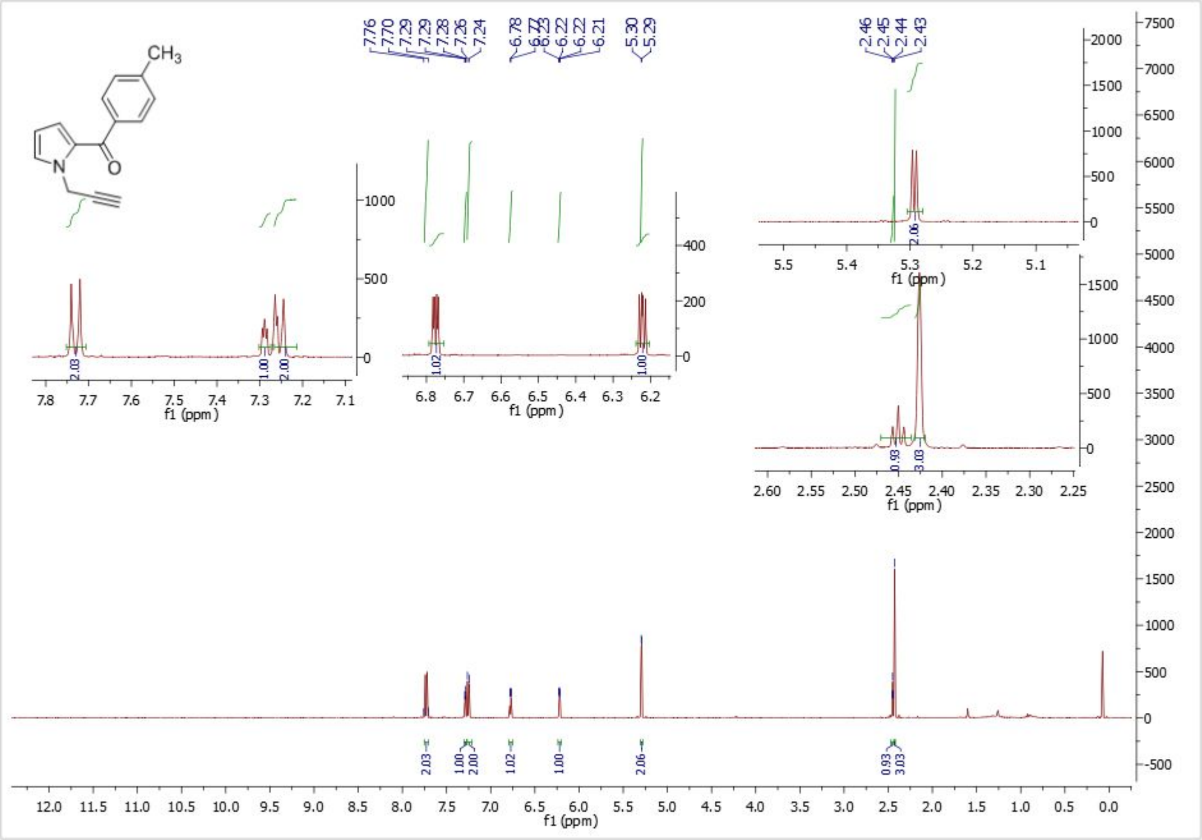

Figure S9.

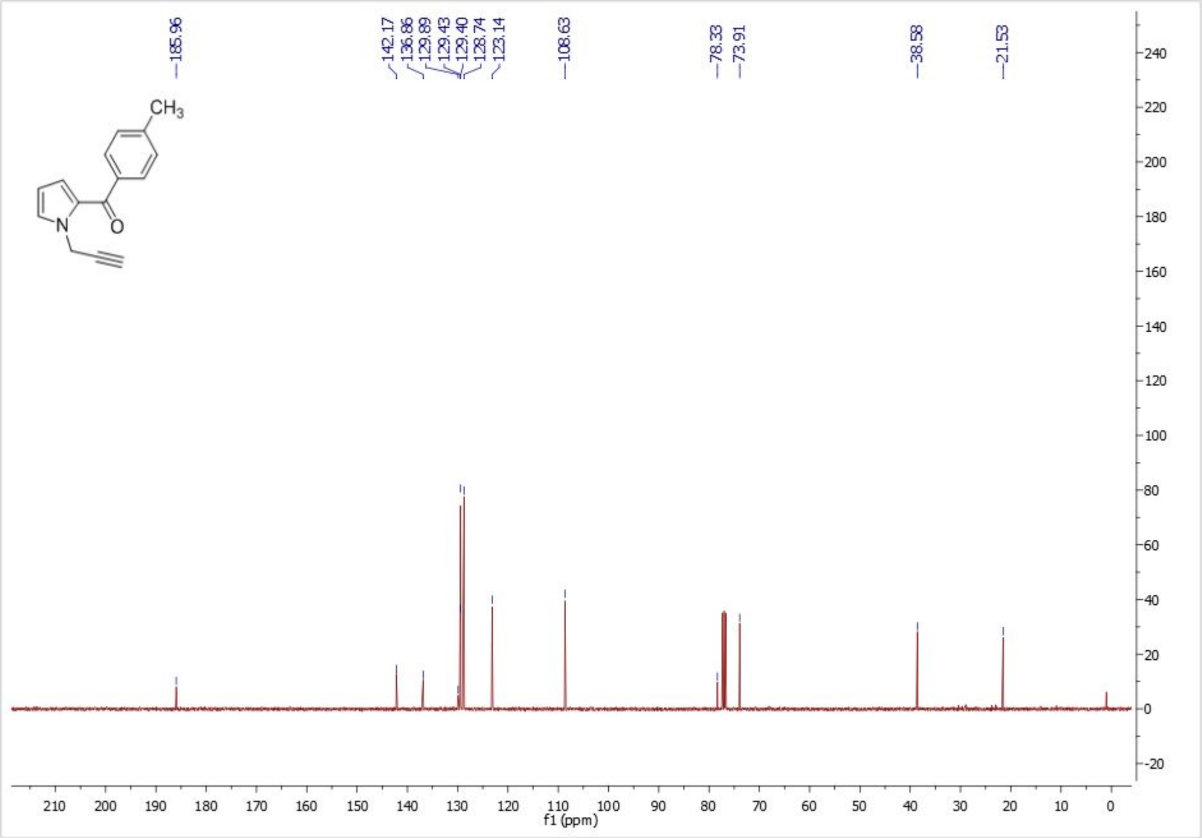

Figure S10.

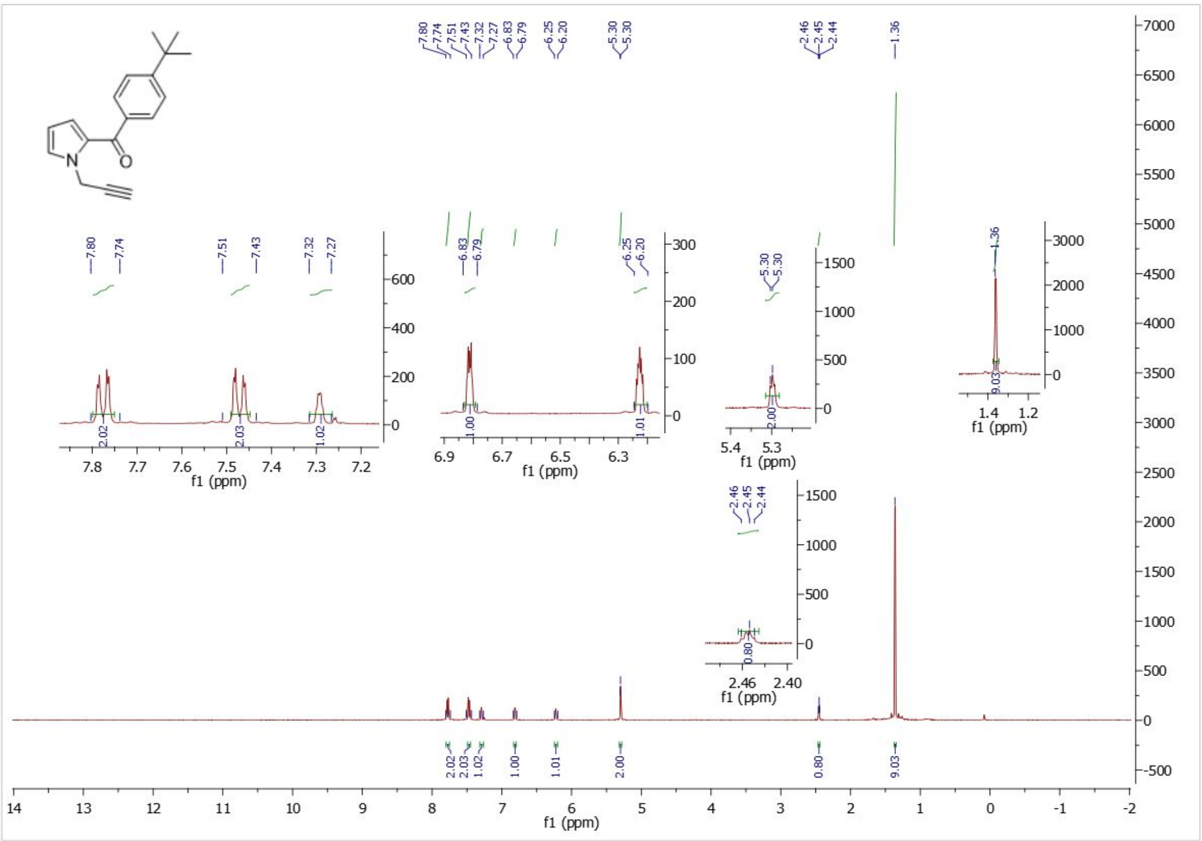

Figure S11.

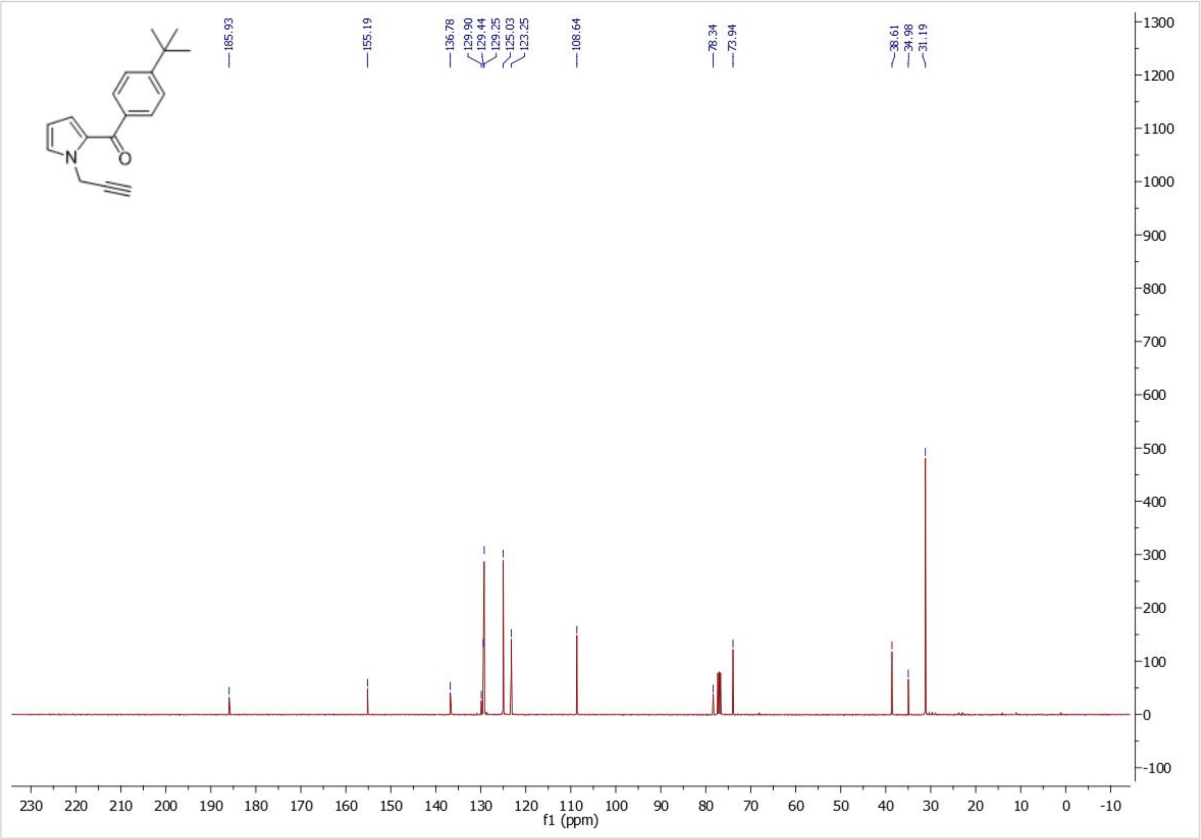

Figure S12.

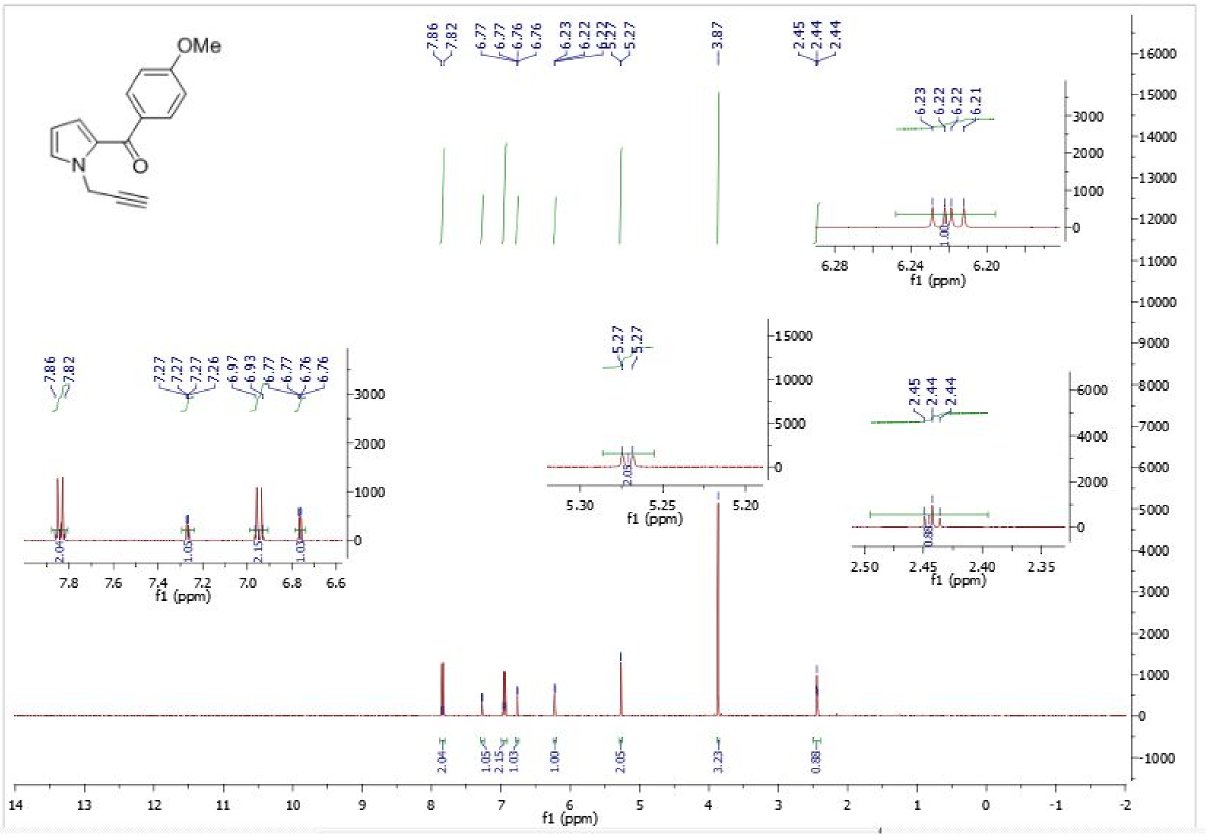

Figure S13.

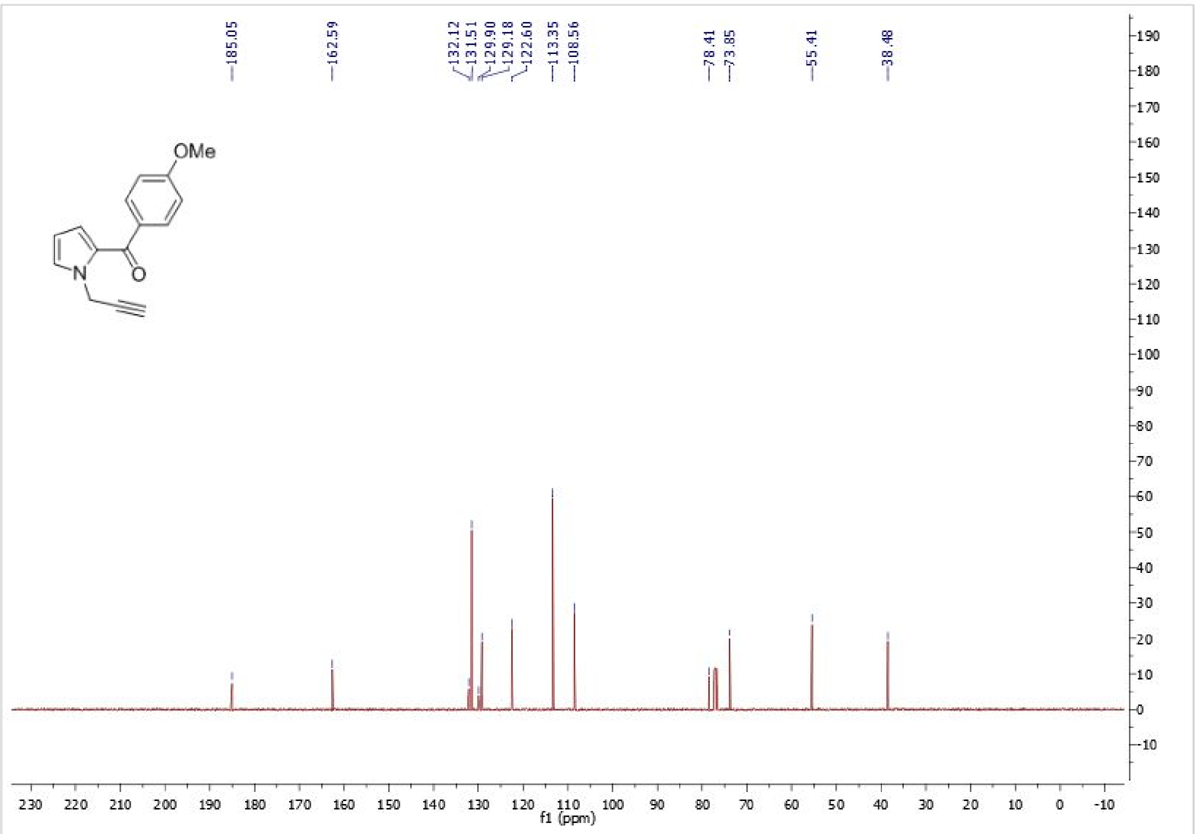

Figure S14.

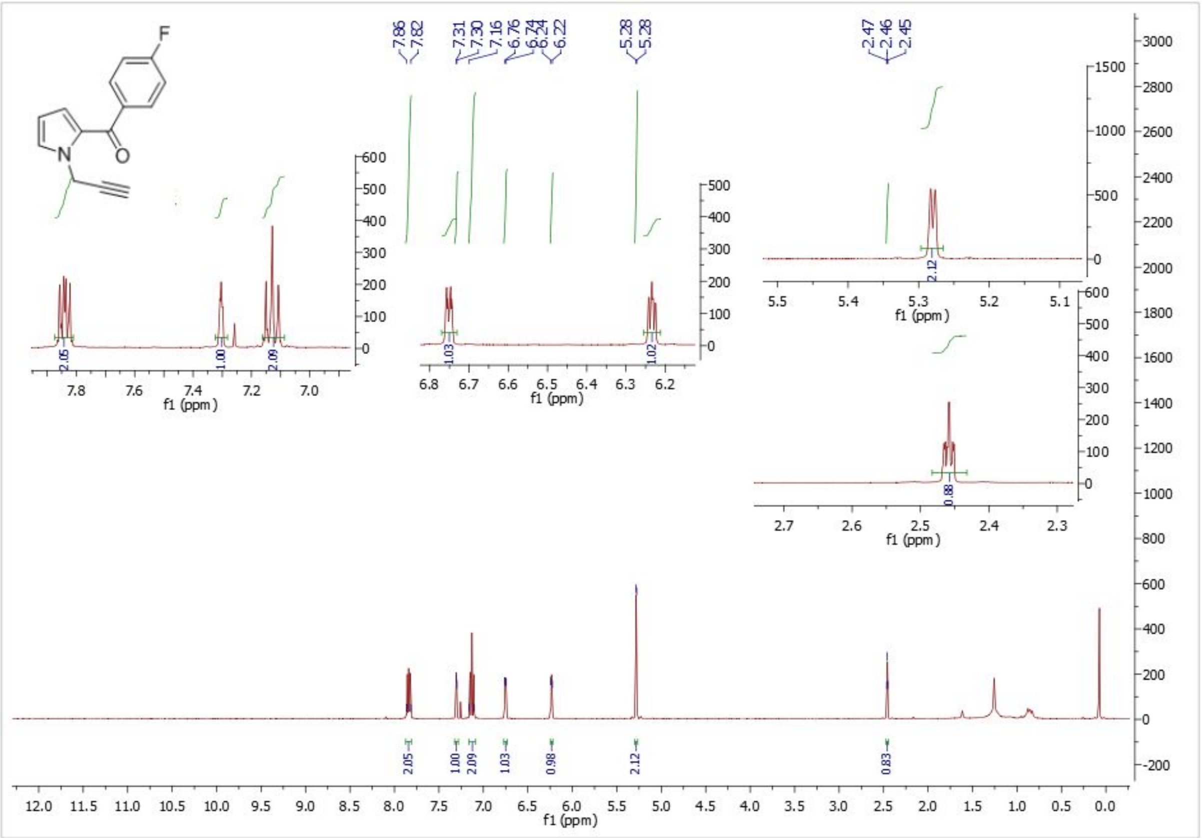

Figure S15.

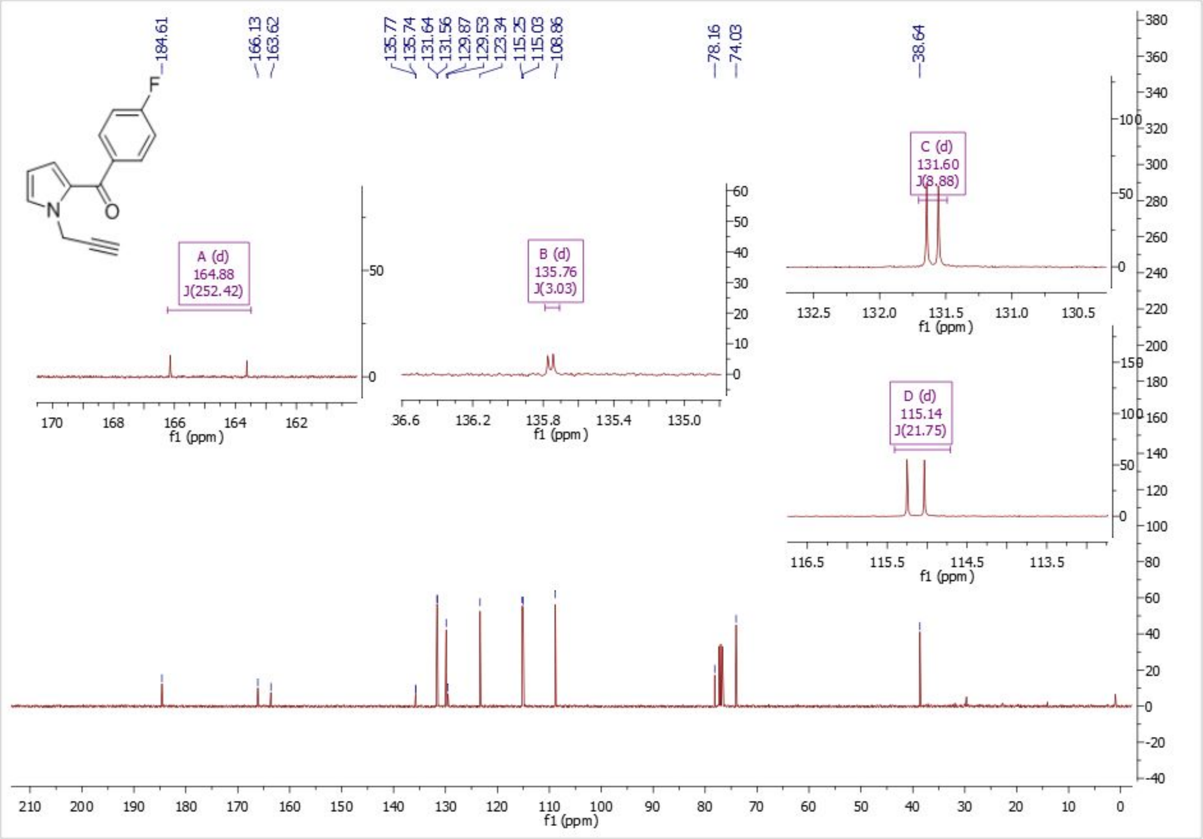

Figure S16.

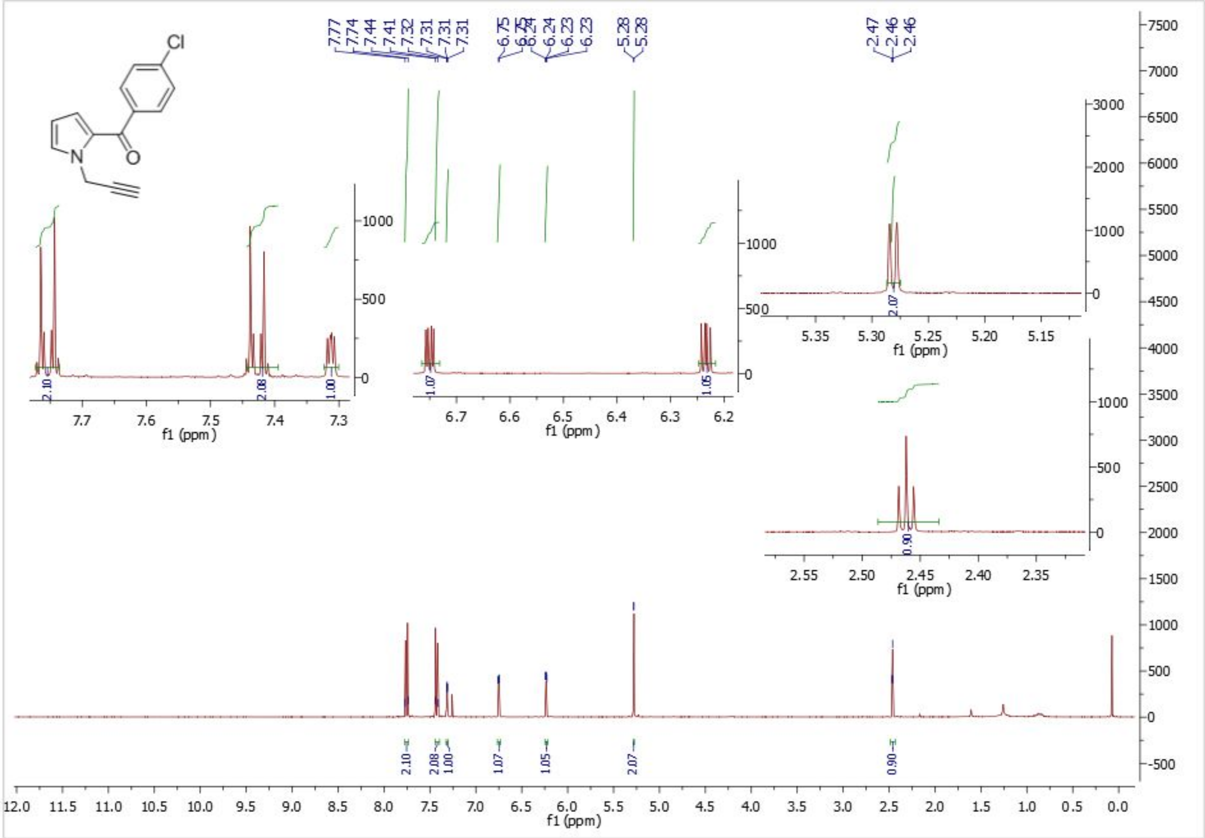

Figure S17.

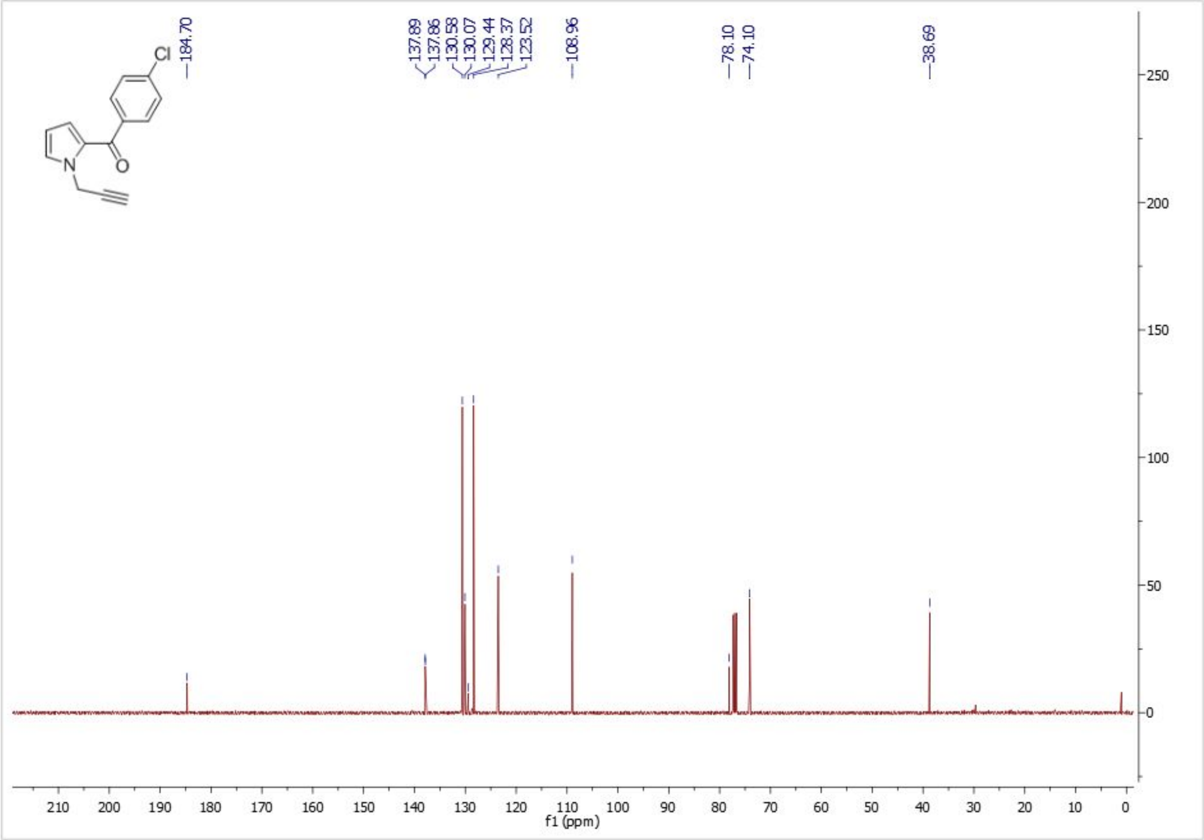

Figure S18.

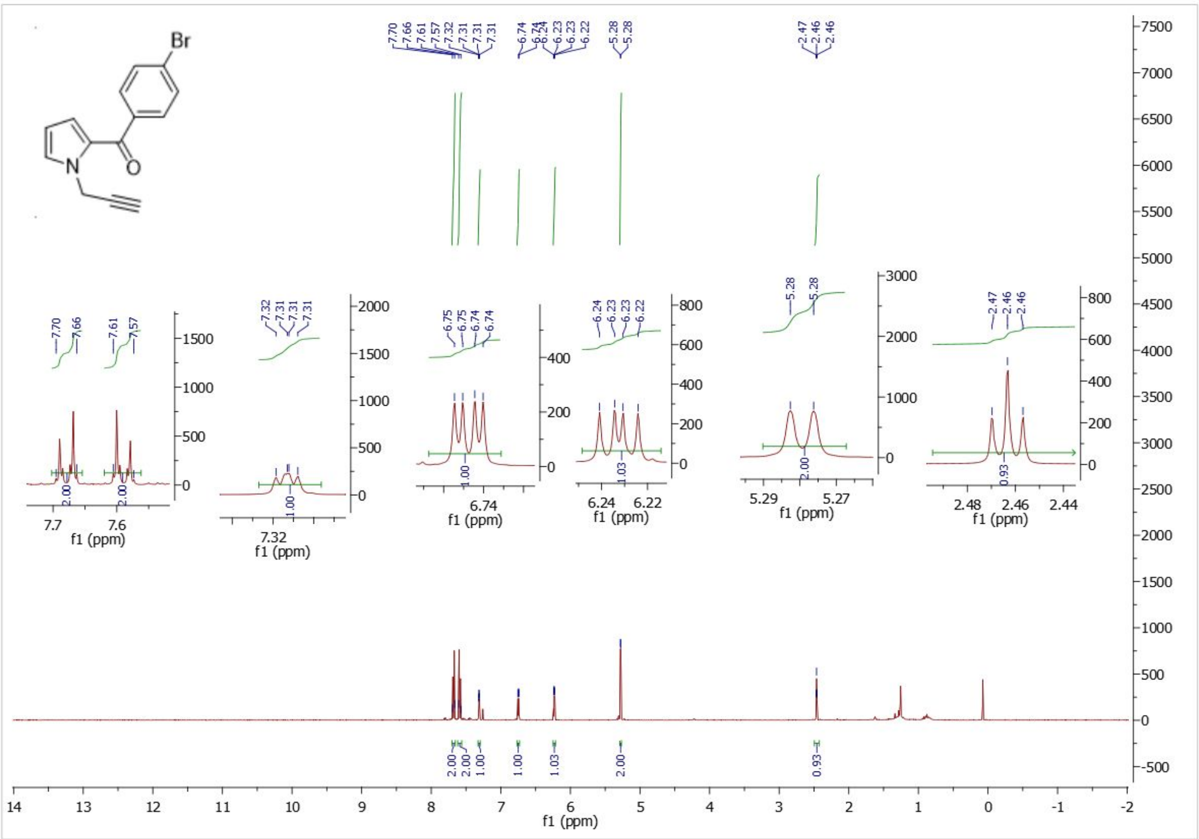

Figure S19.

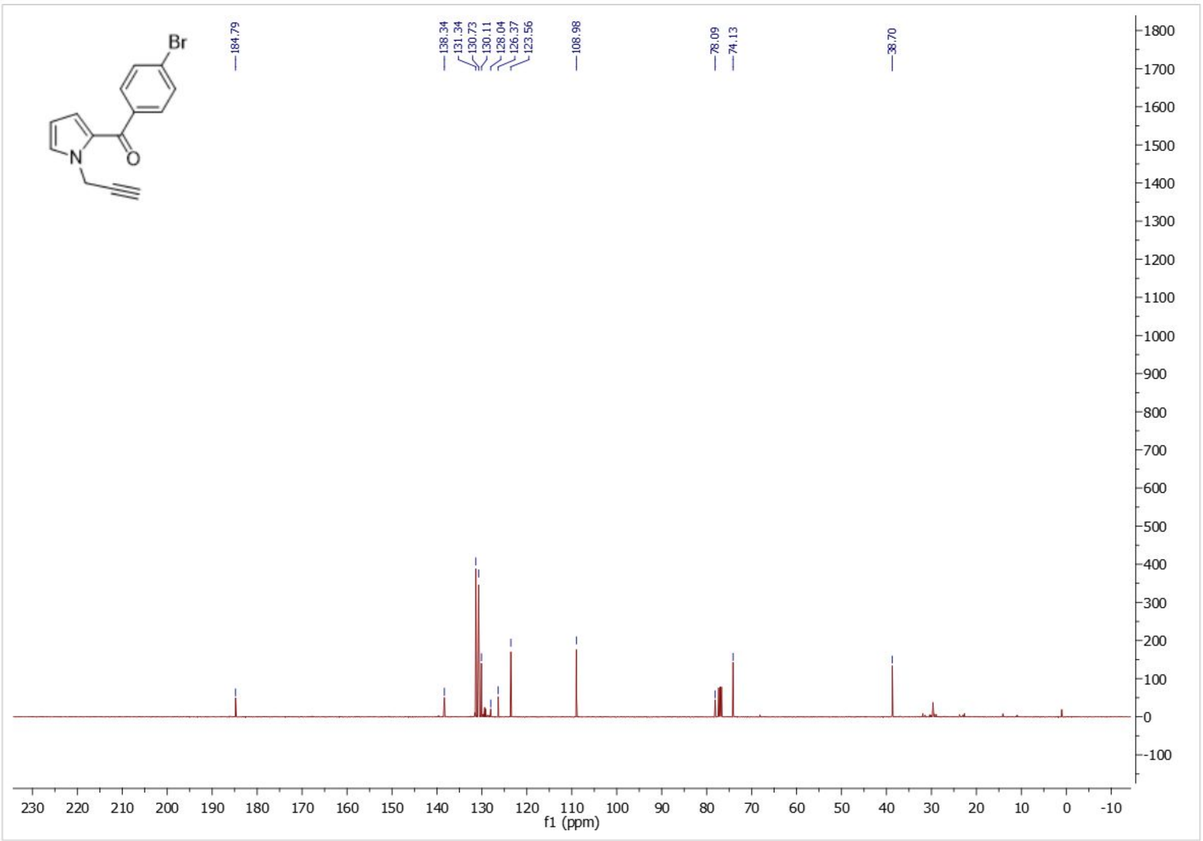

Figure S20.

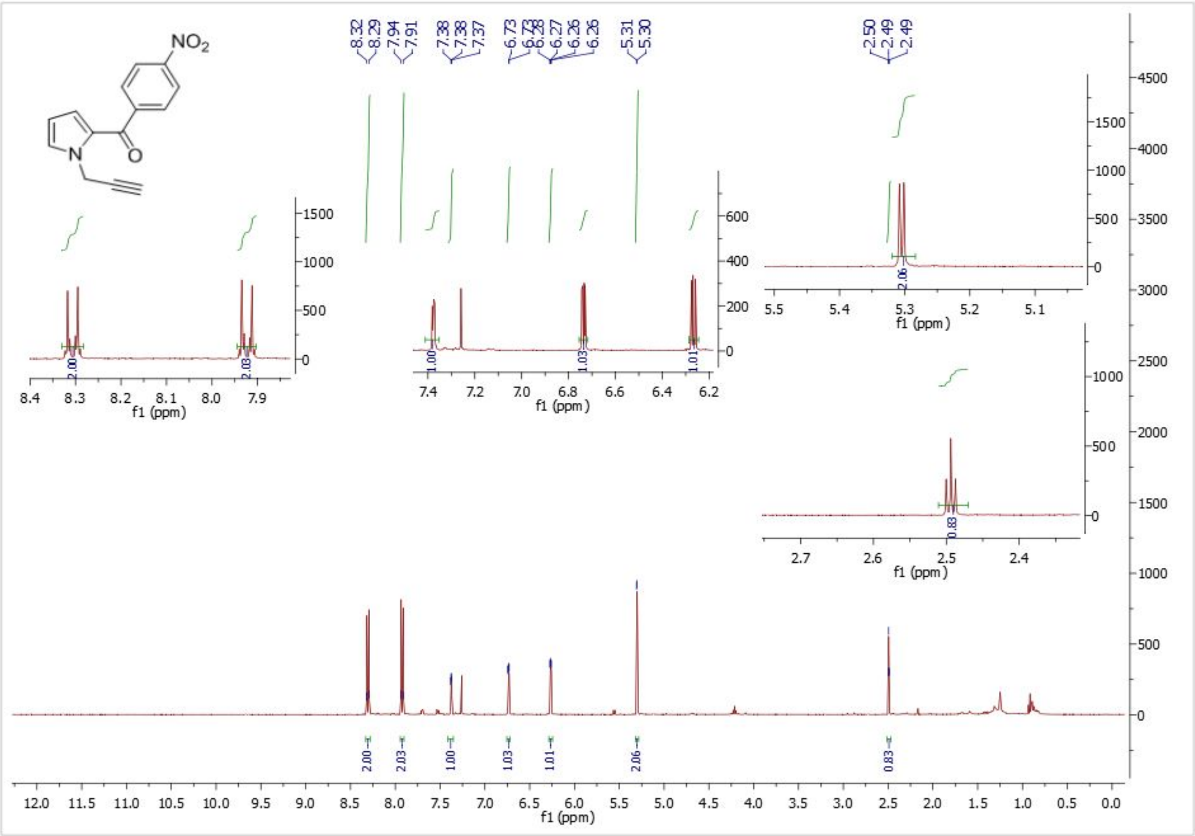

Figure S21.

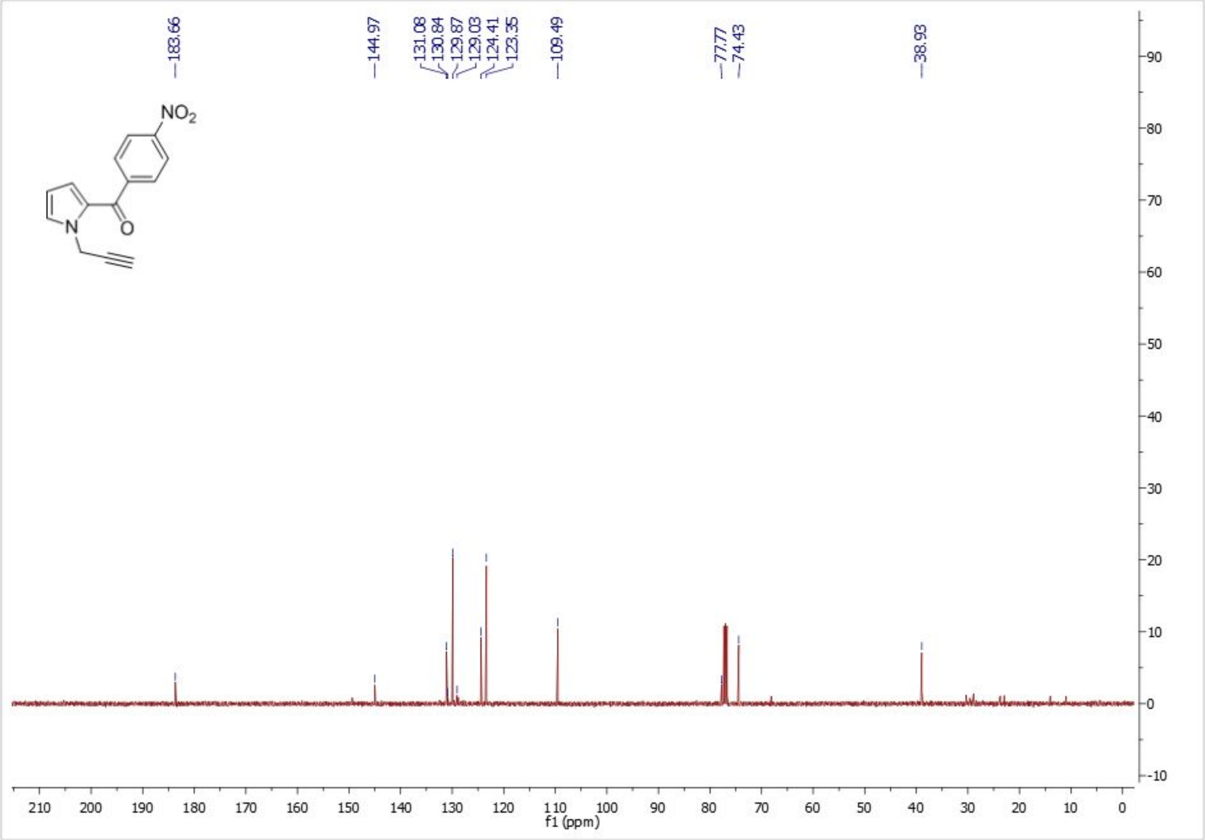
183.66
144.97
131.08
130.84
129.87
129.03
124.41
123.35
109.49
77.77
74.43
38.93
NO2
N
O
f1 (ppm)

Figure S22.

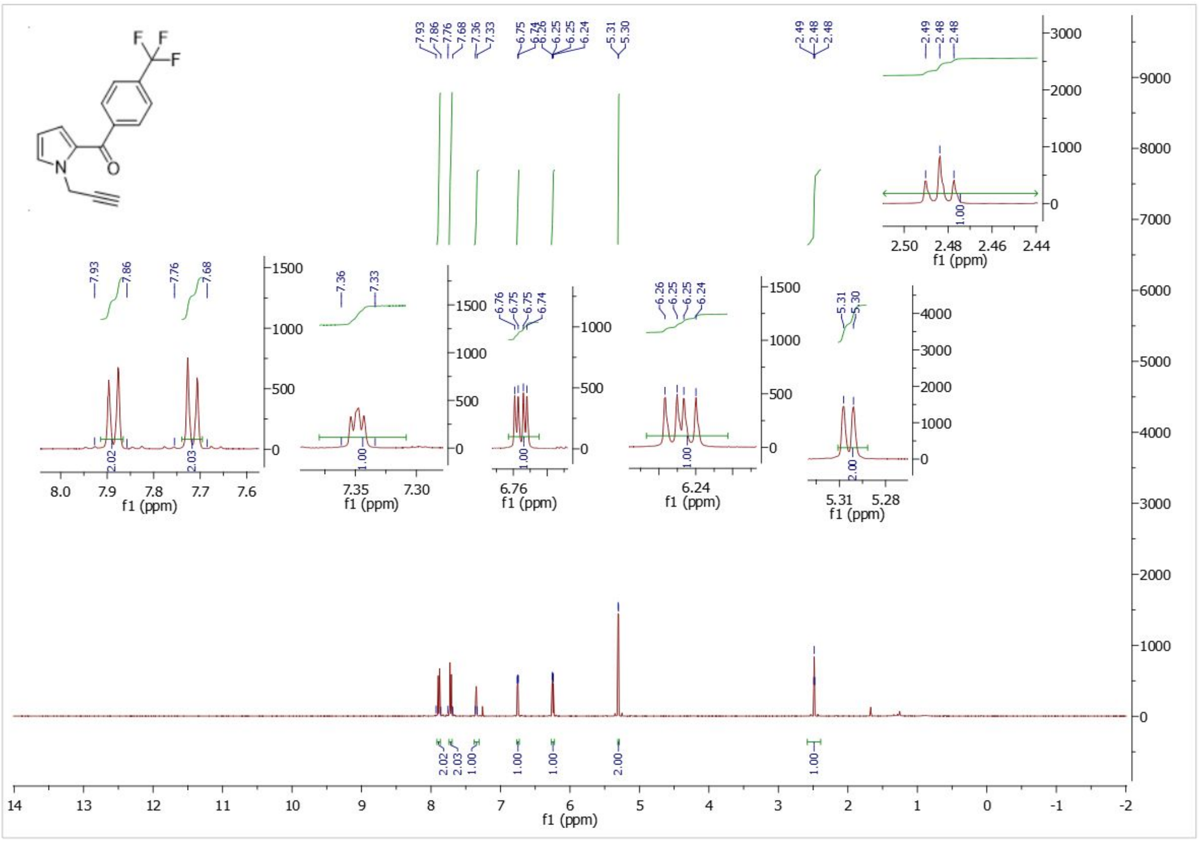
7.93
7.86
7.76
7.68
7.36
7.33
6.75
6.74
6.26
6.25
6.25
6.24
5.31
5.30
2.49
2.48
2.48
F
F
F
N
O
2.02
2.03
1.00
1.00
1.00
2.00
1.00
f1 (ppm)

Figure S23.

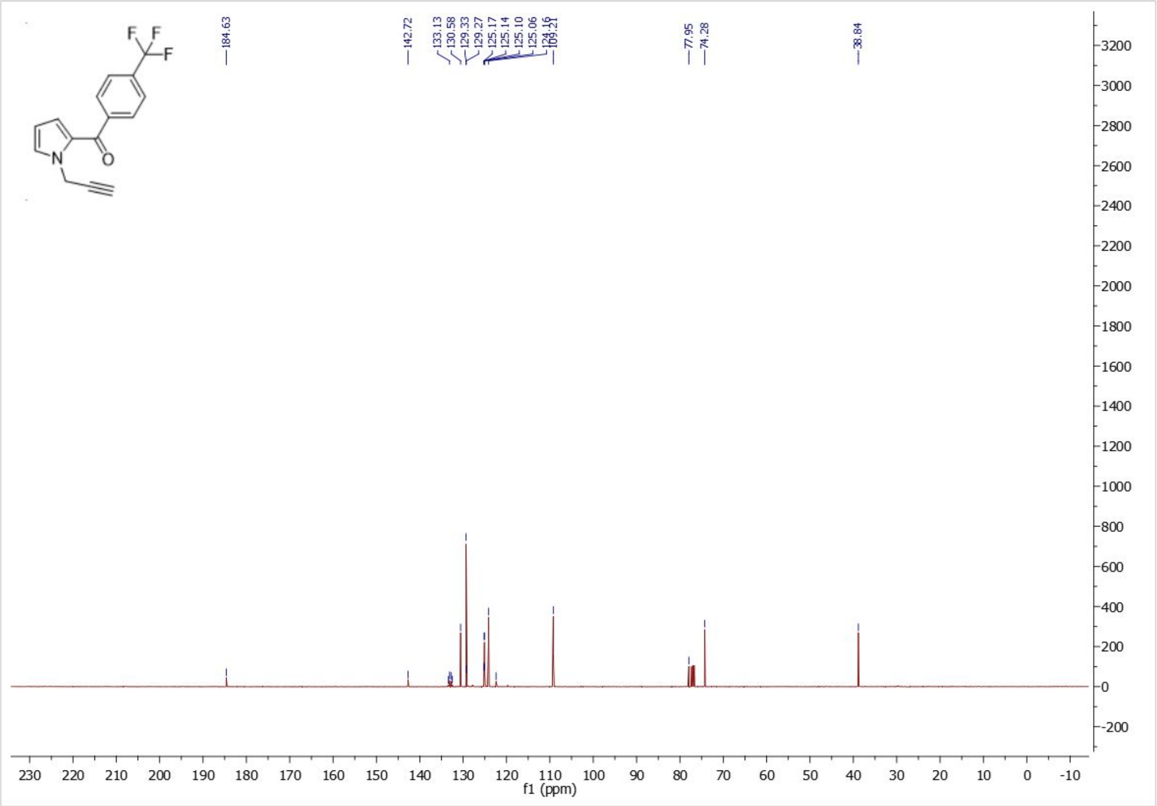

Figure S24.

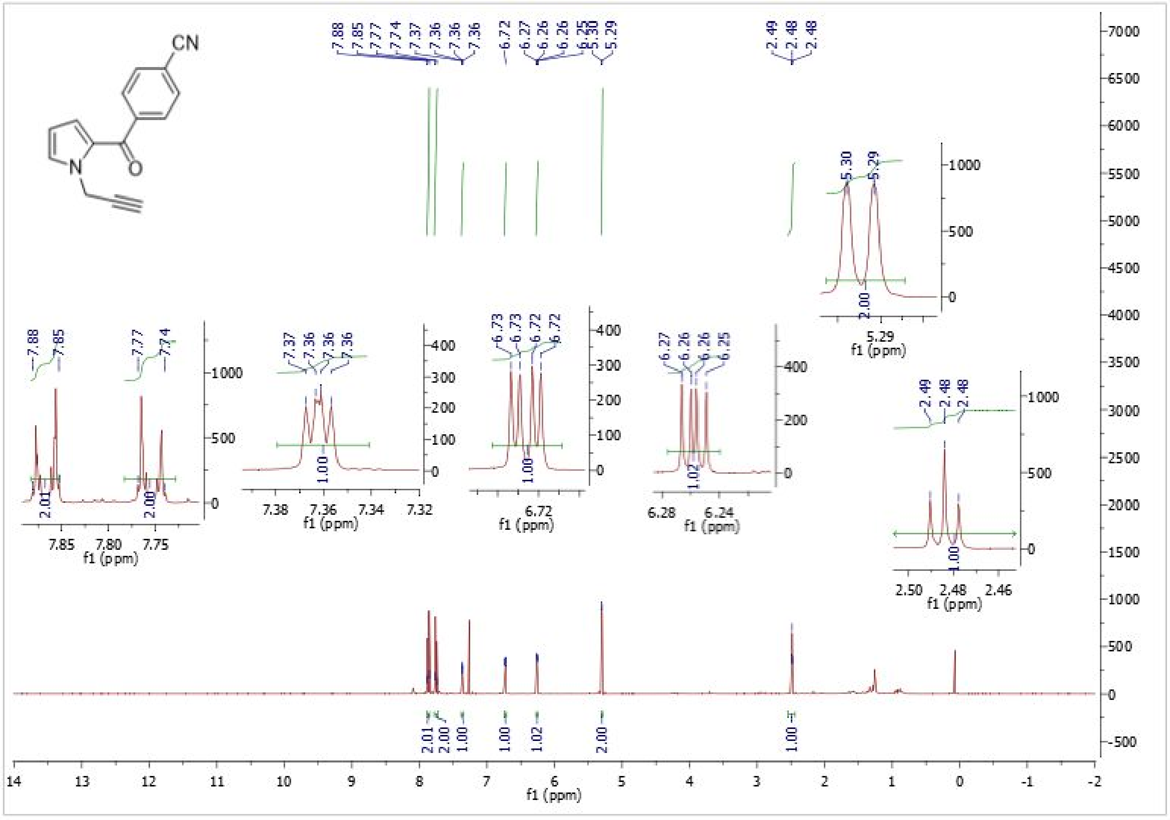

Figure S25.

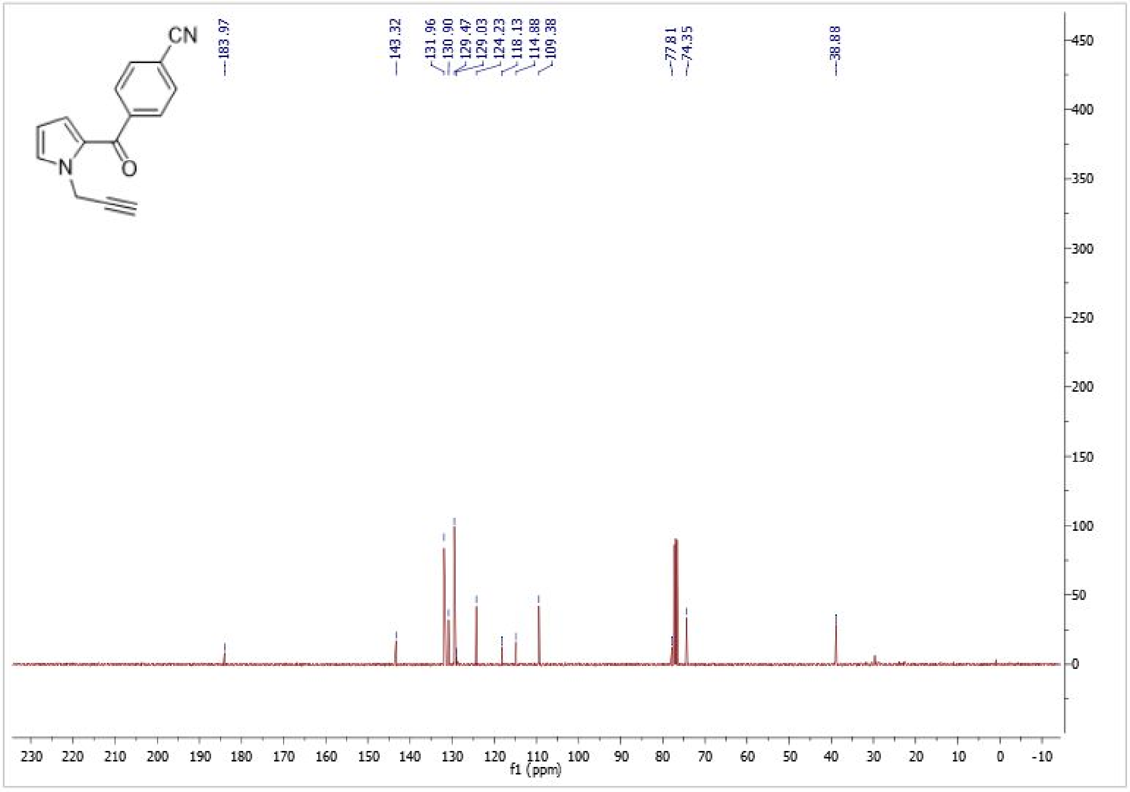

Figure S26.

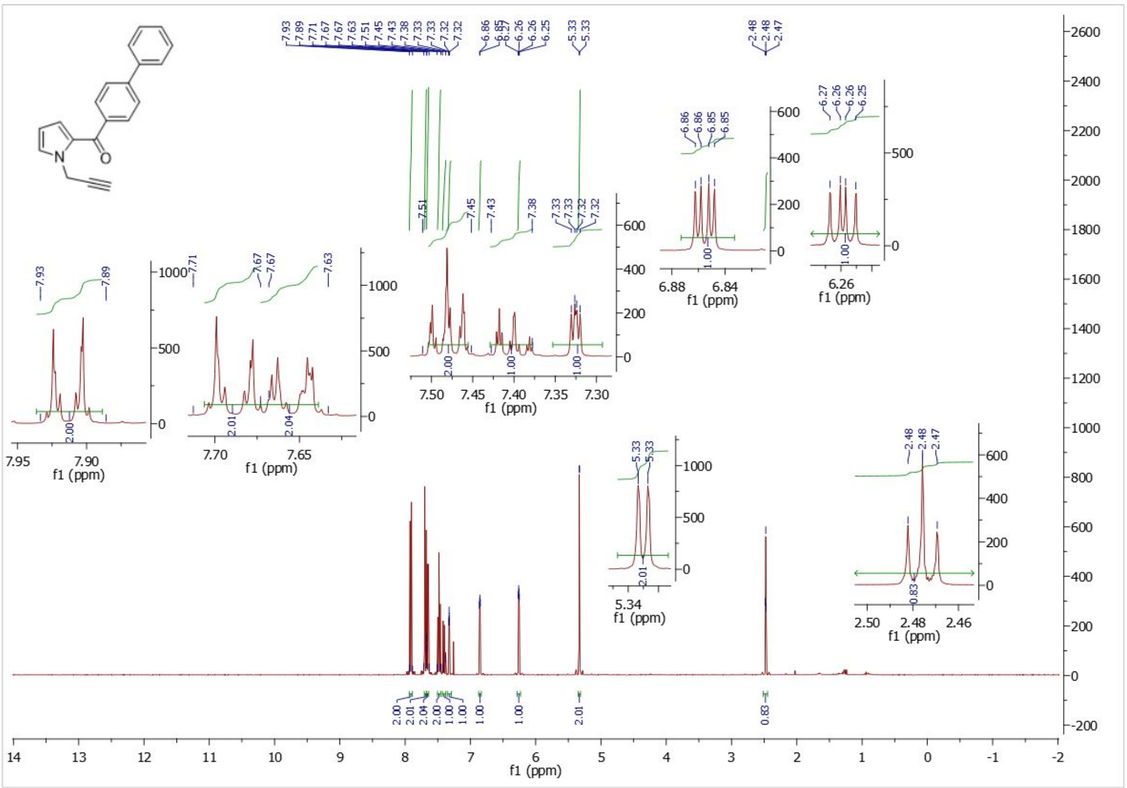

Figure S27.

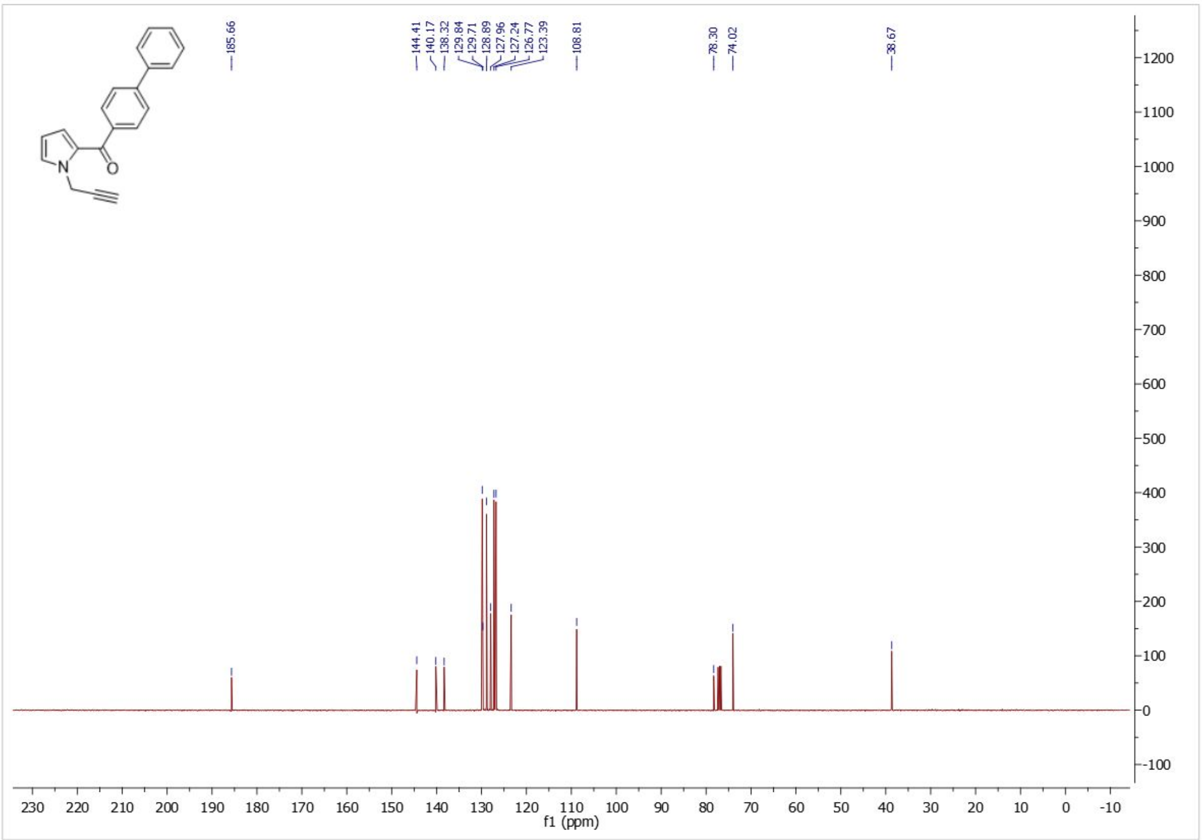

Figure S28.

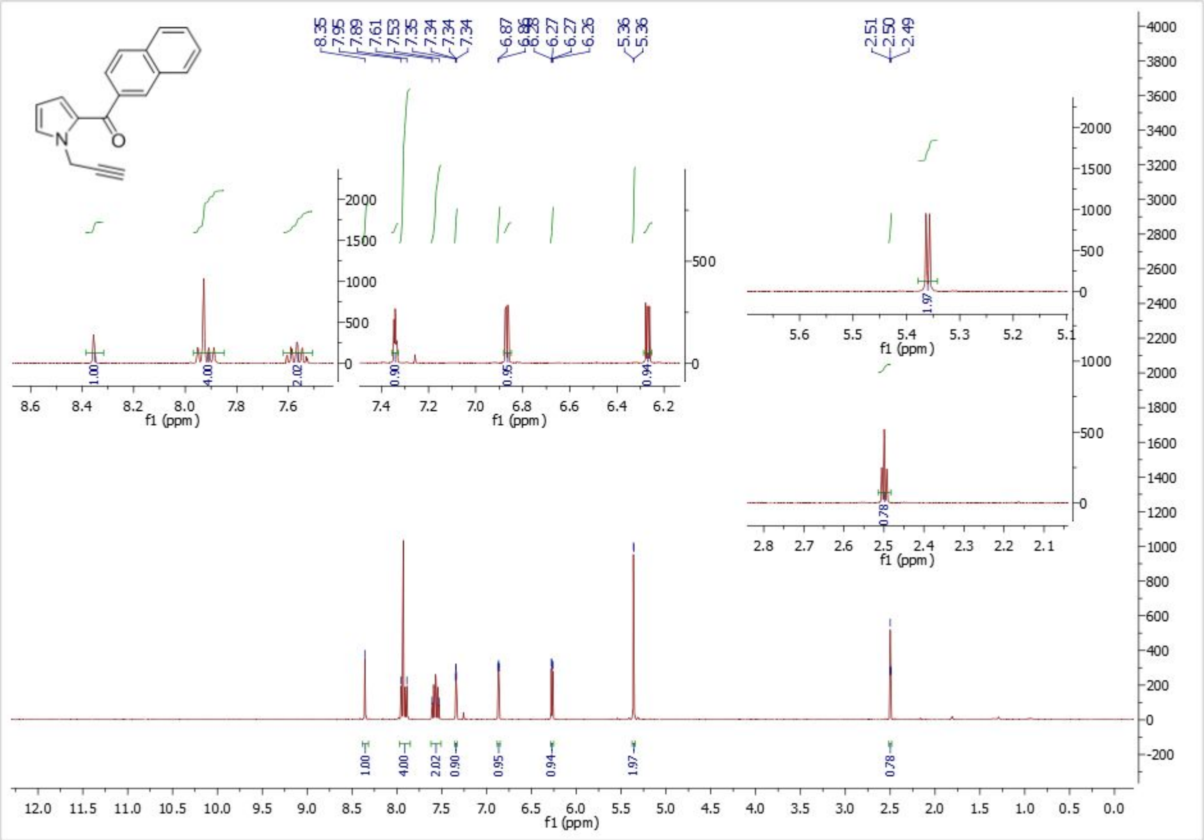

Figure S29.

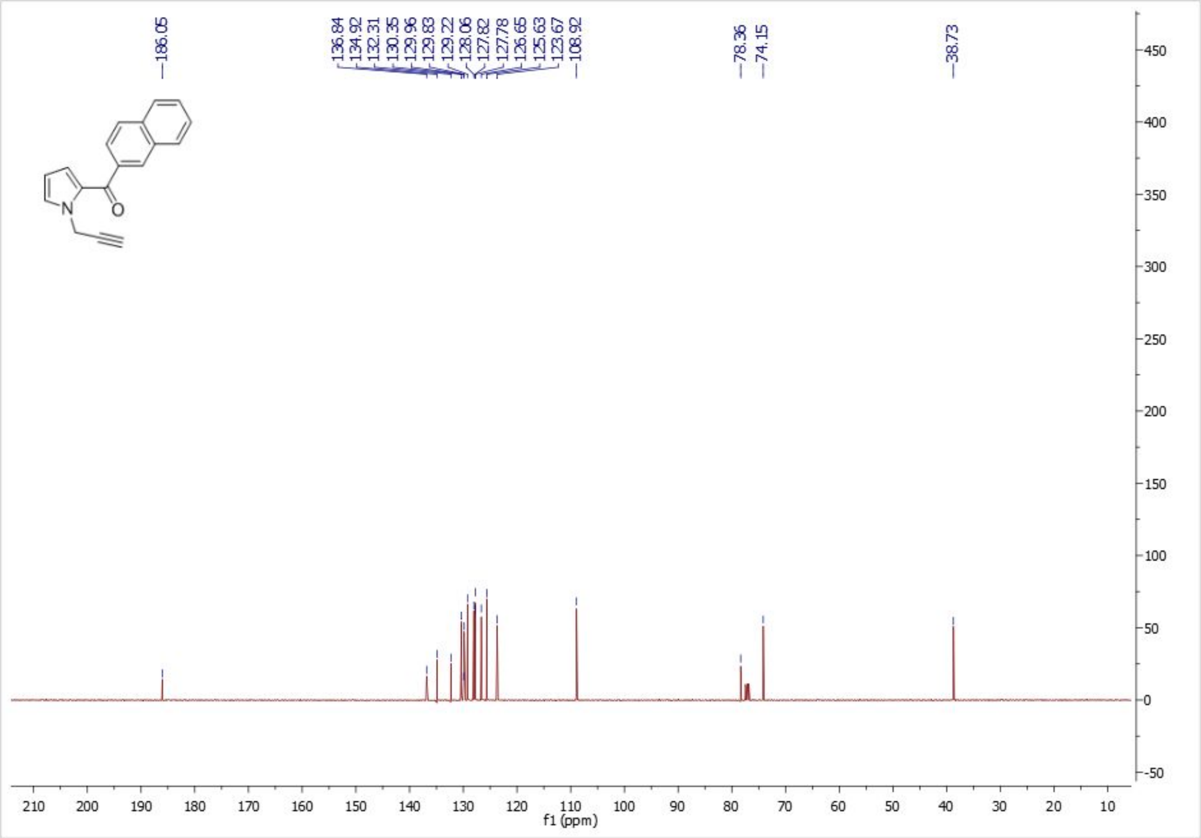

Figure S30.

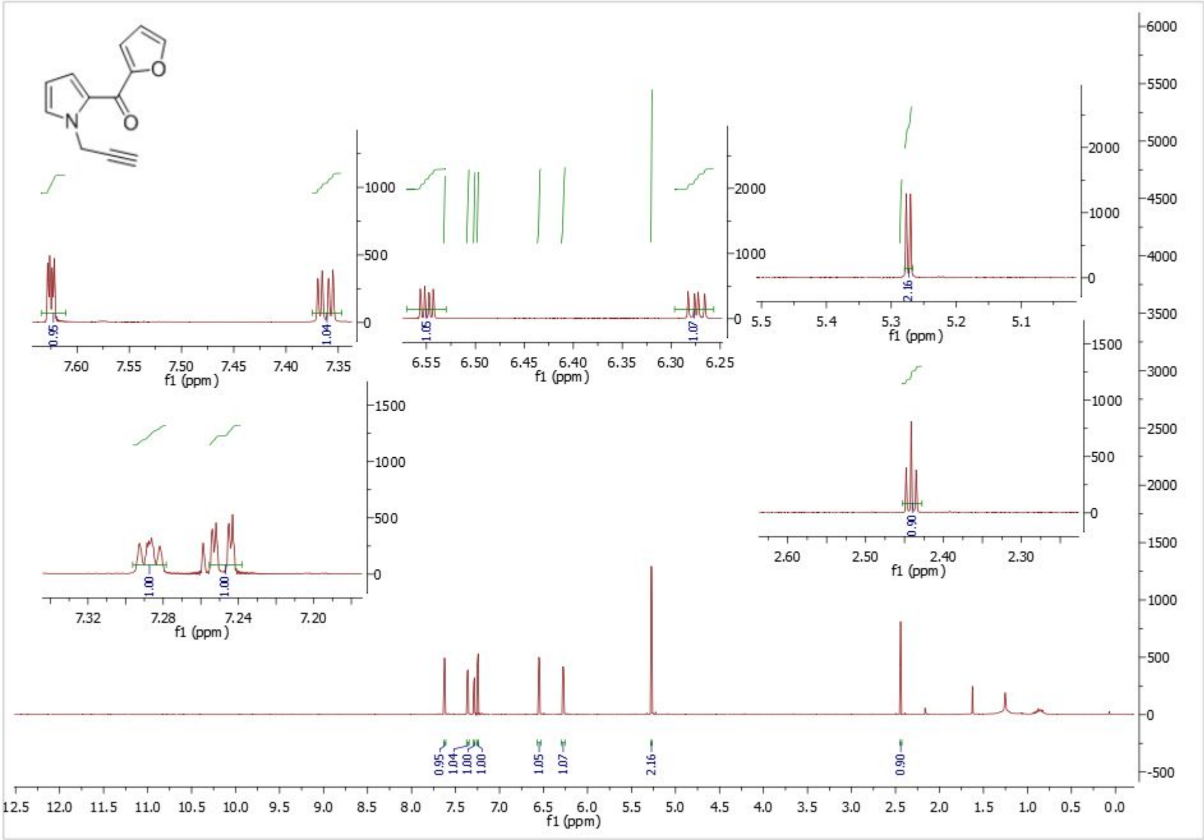

Figure S31.

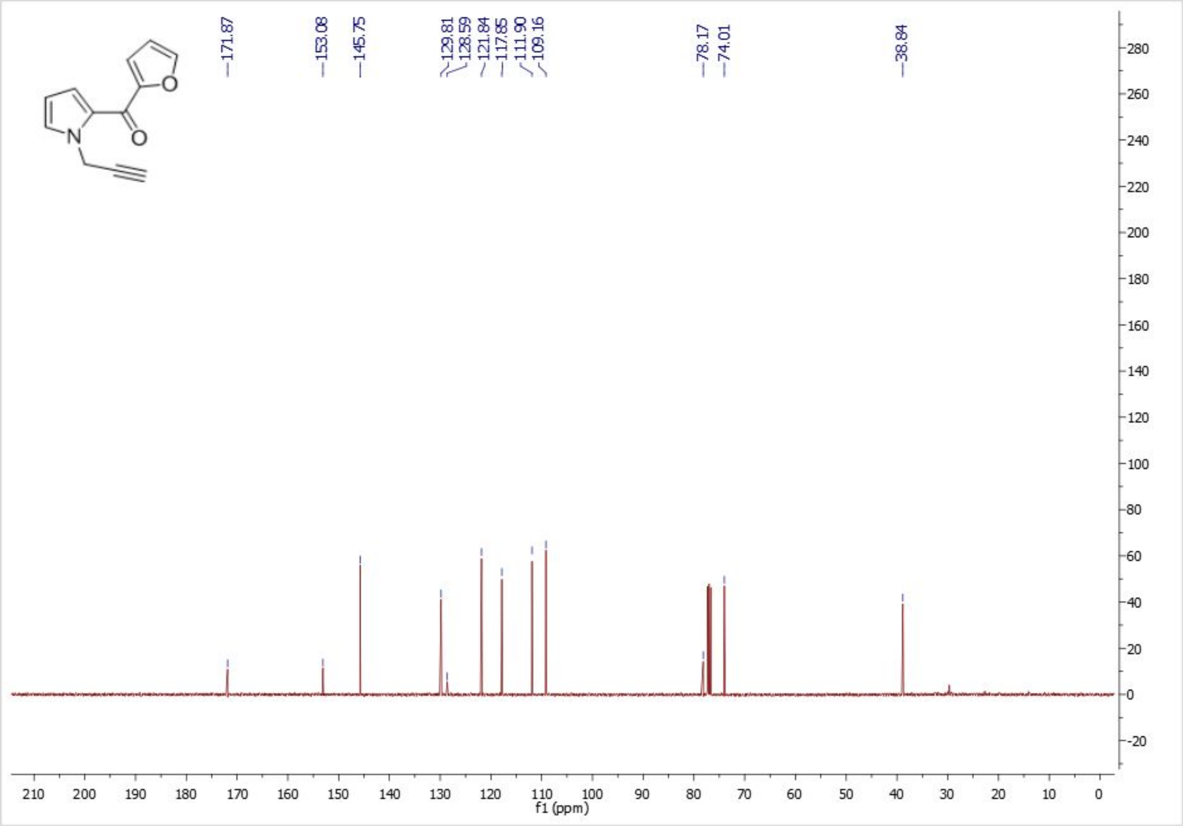

Figure S32.

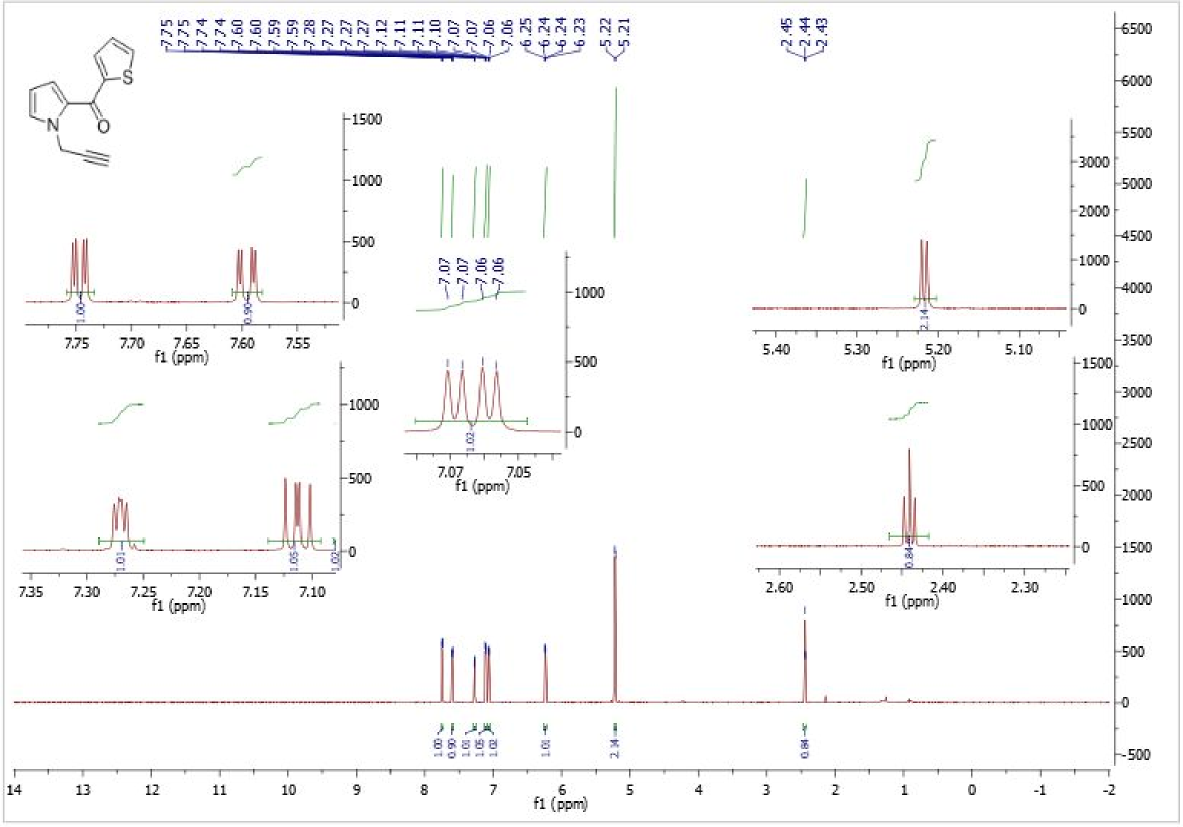

Figure S33.

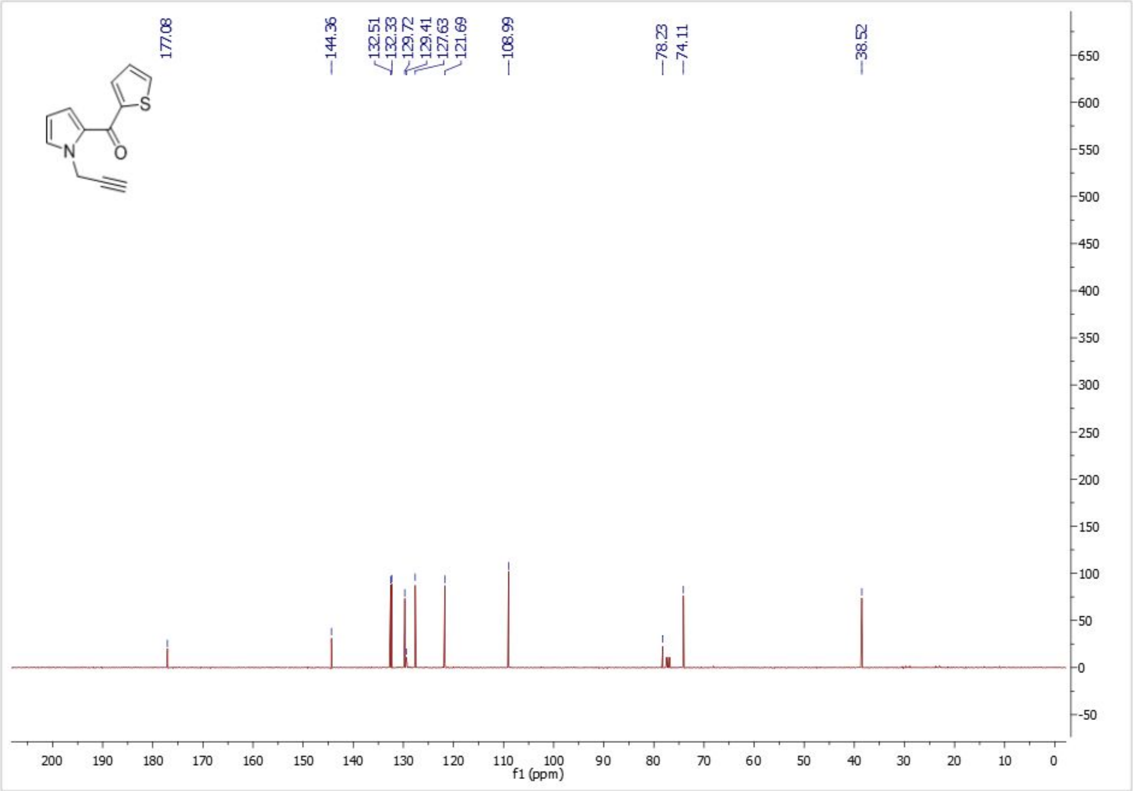

Figure S34.

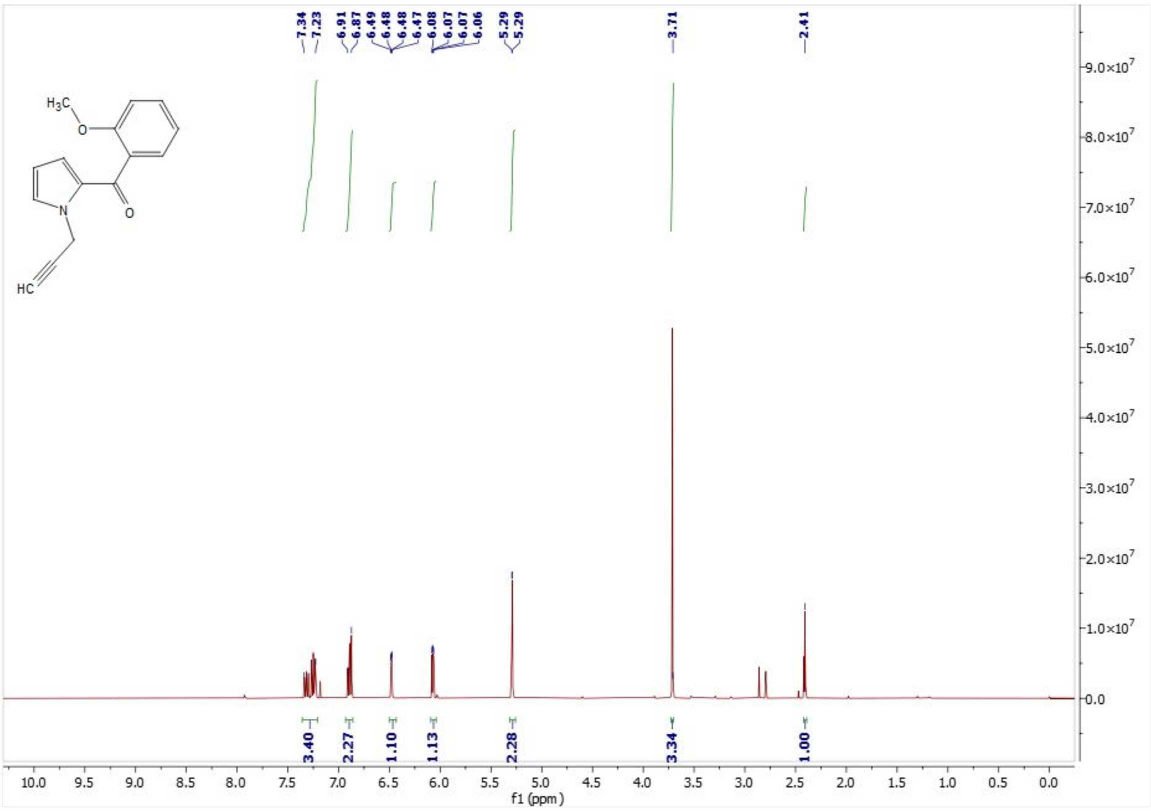

Figure S35.

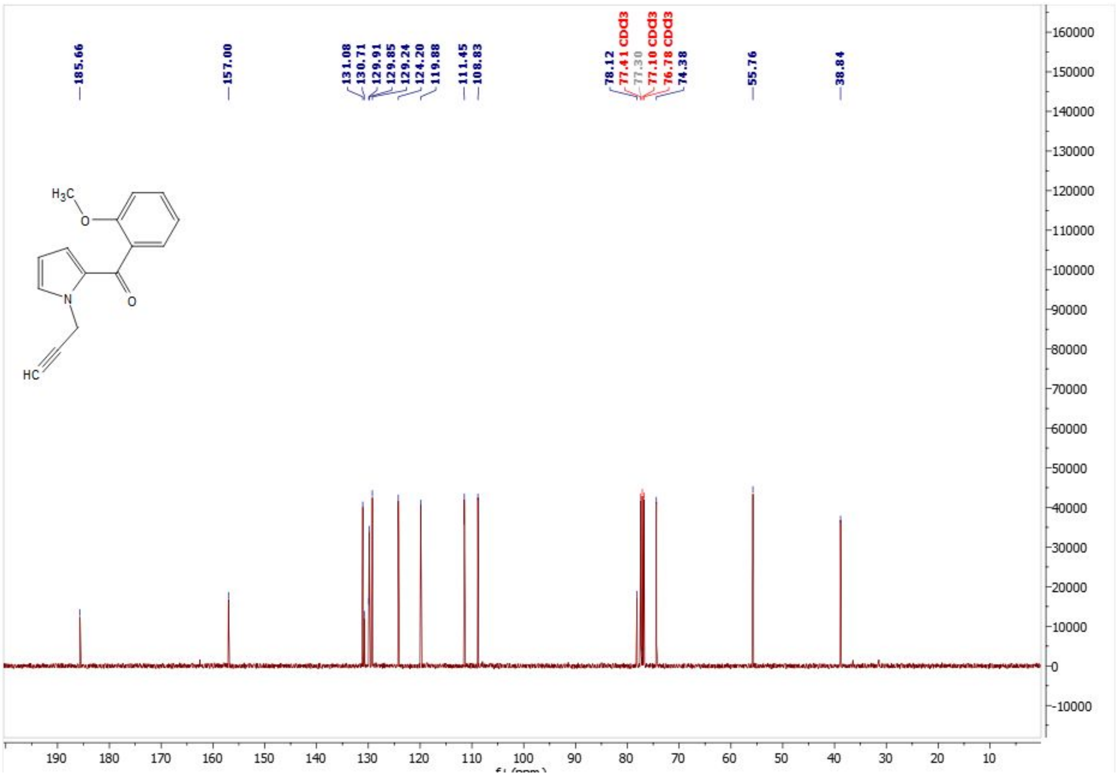

Figure S36.

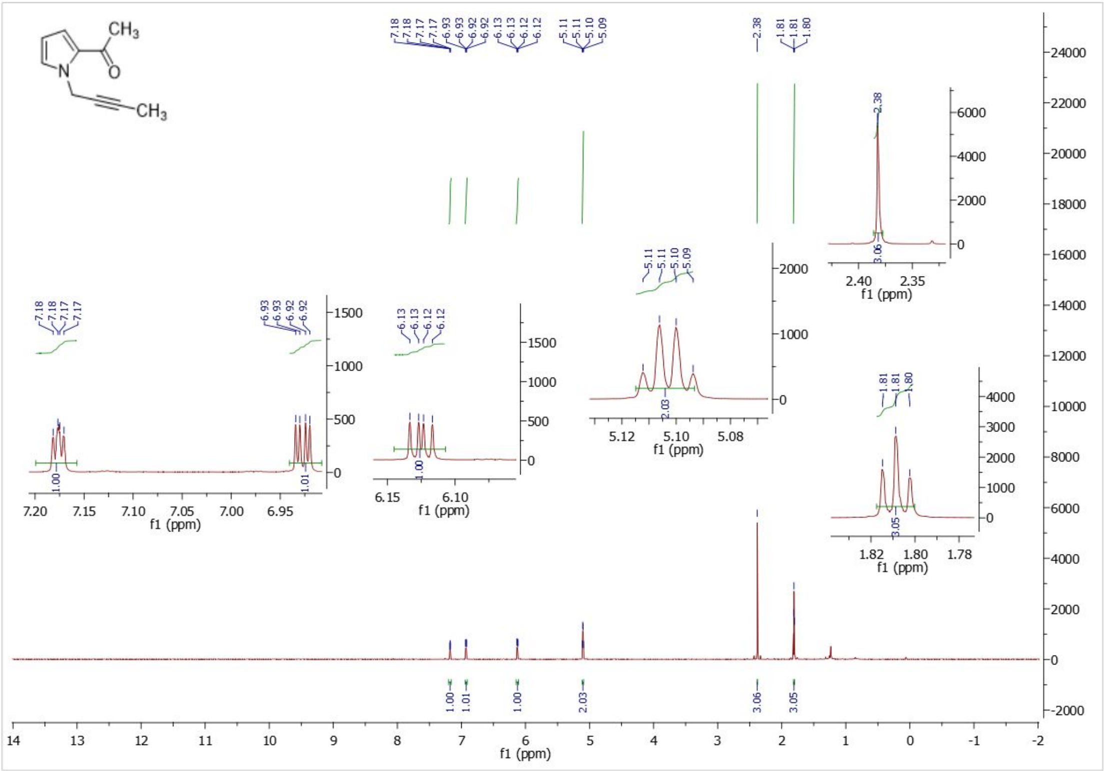

Figure S37.

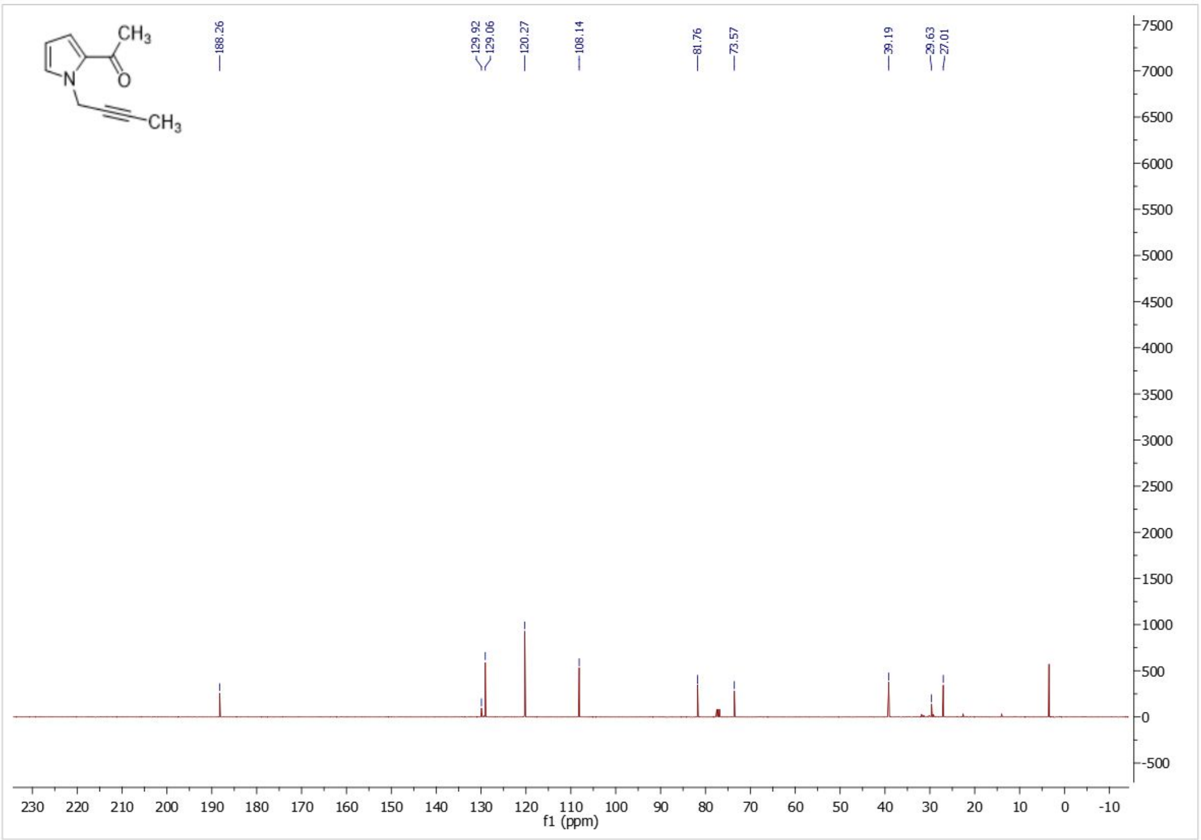

Figure S38.

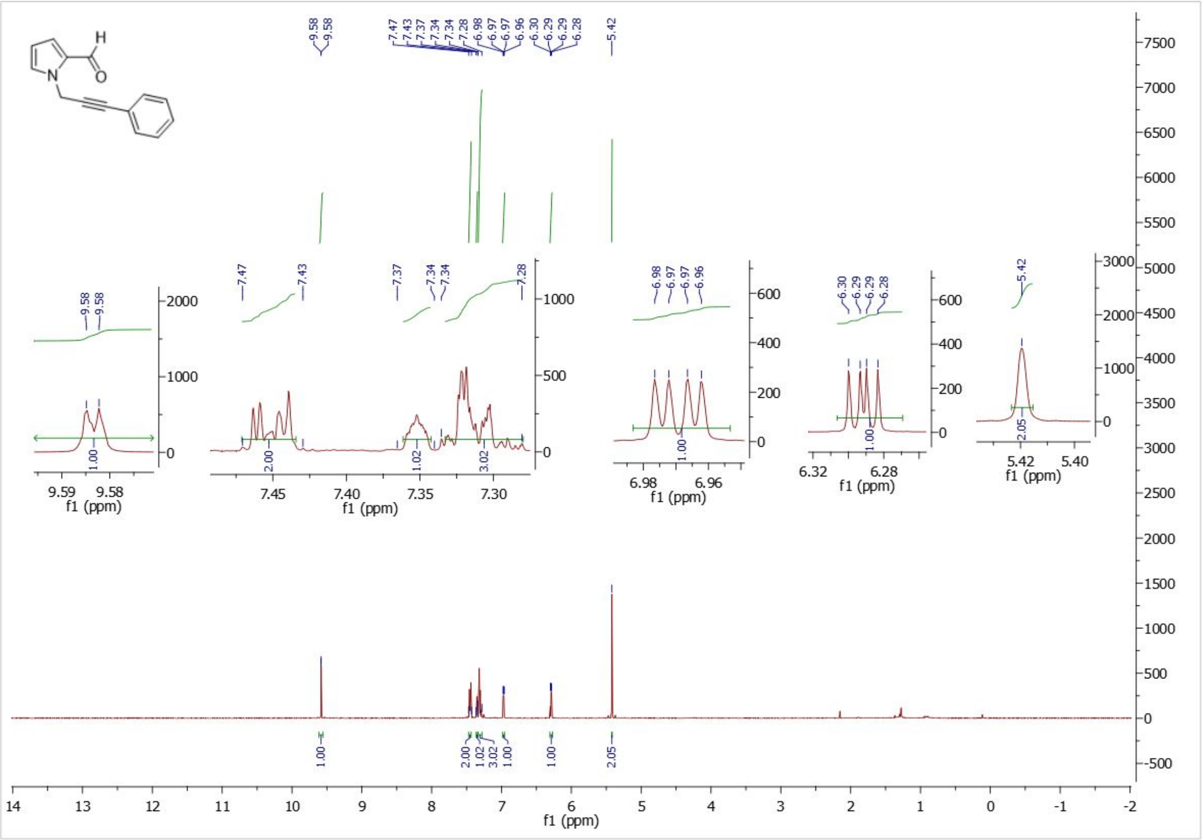

Figure S39.

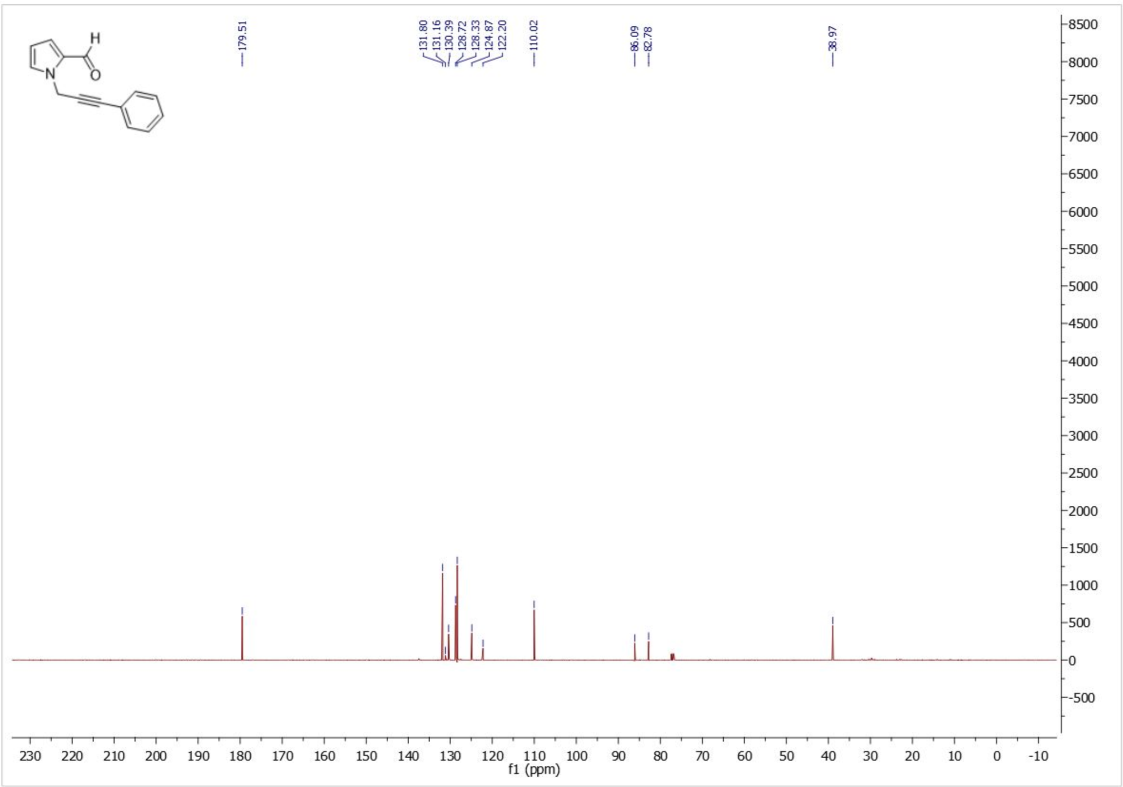

Figure S40.

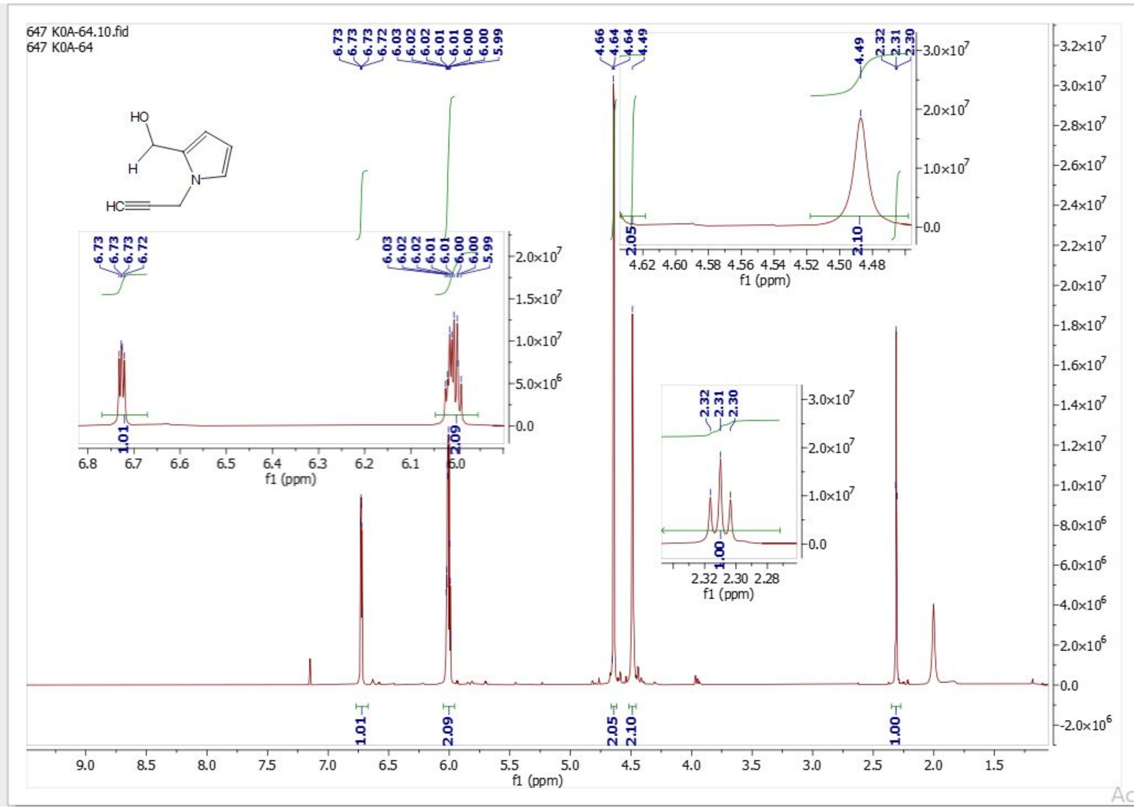

Figure S41.

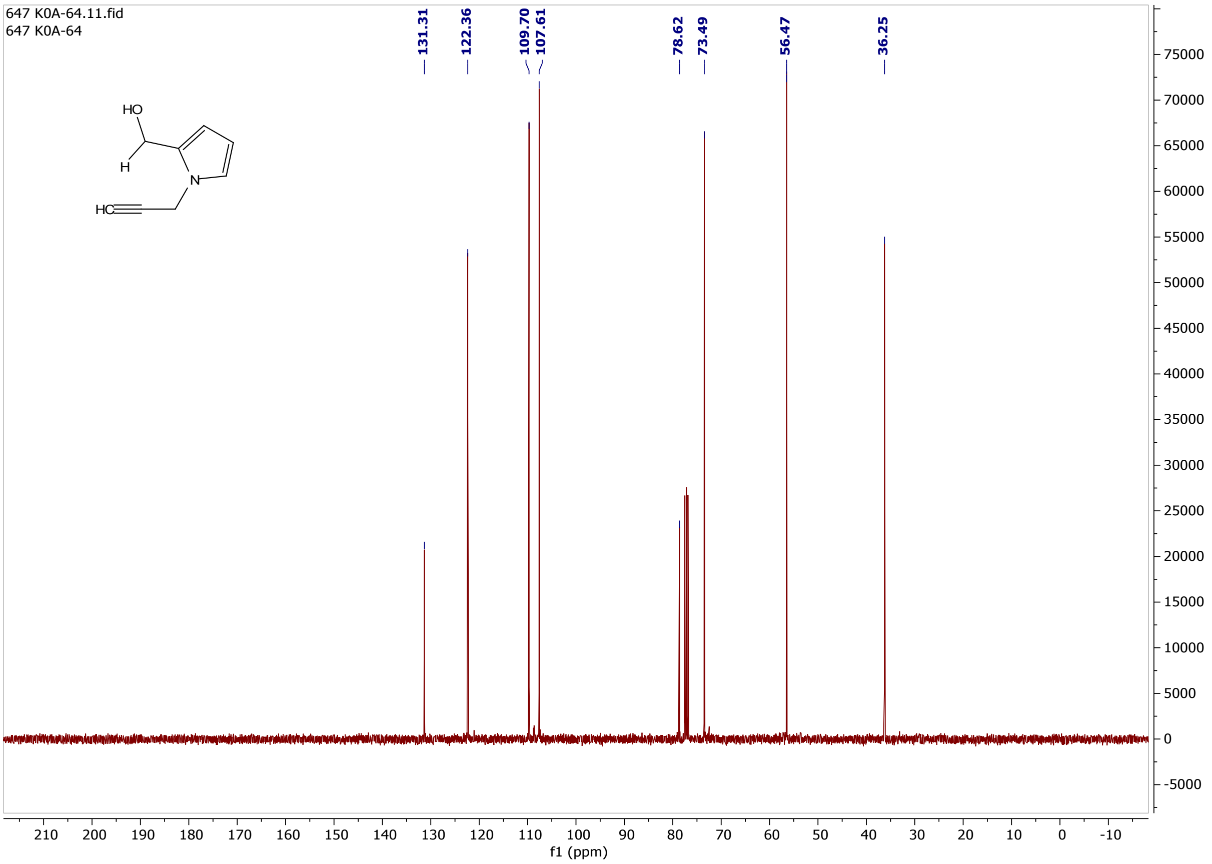

Figure S42.

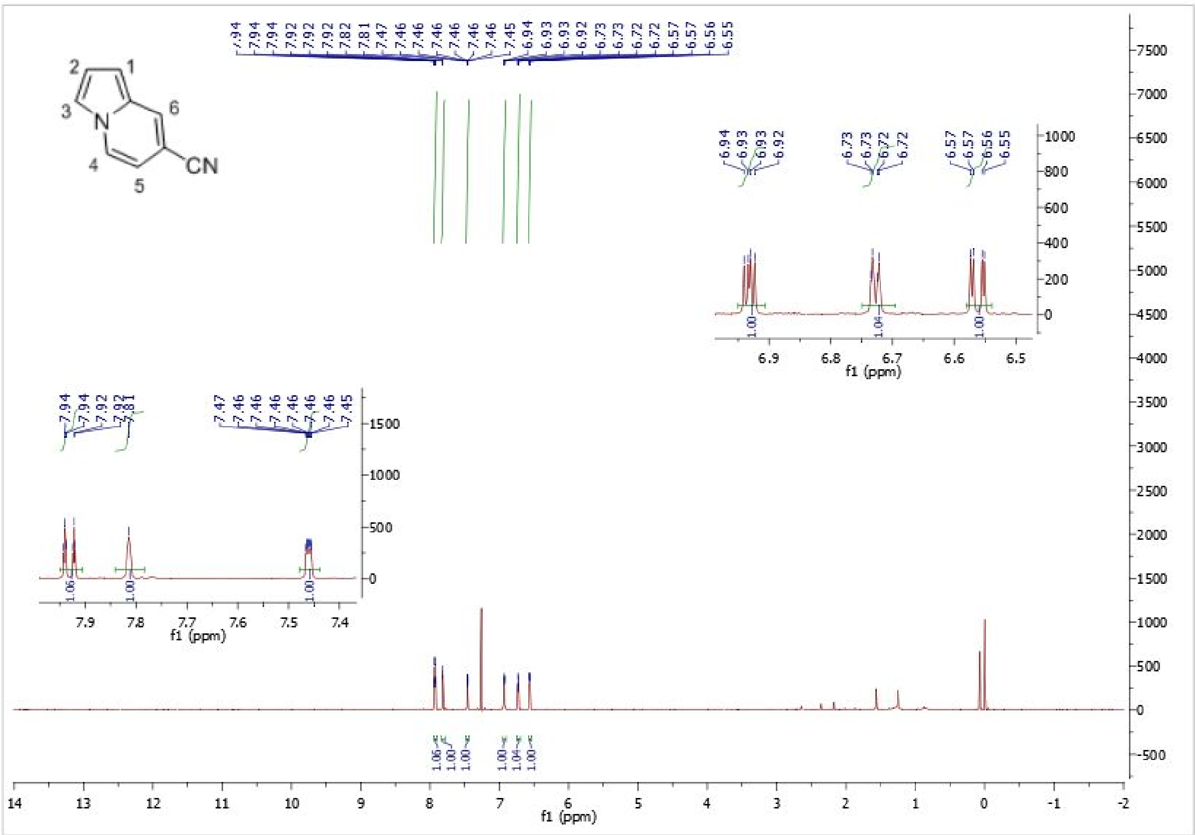

Figure S43

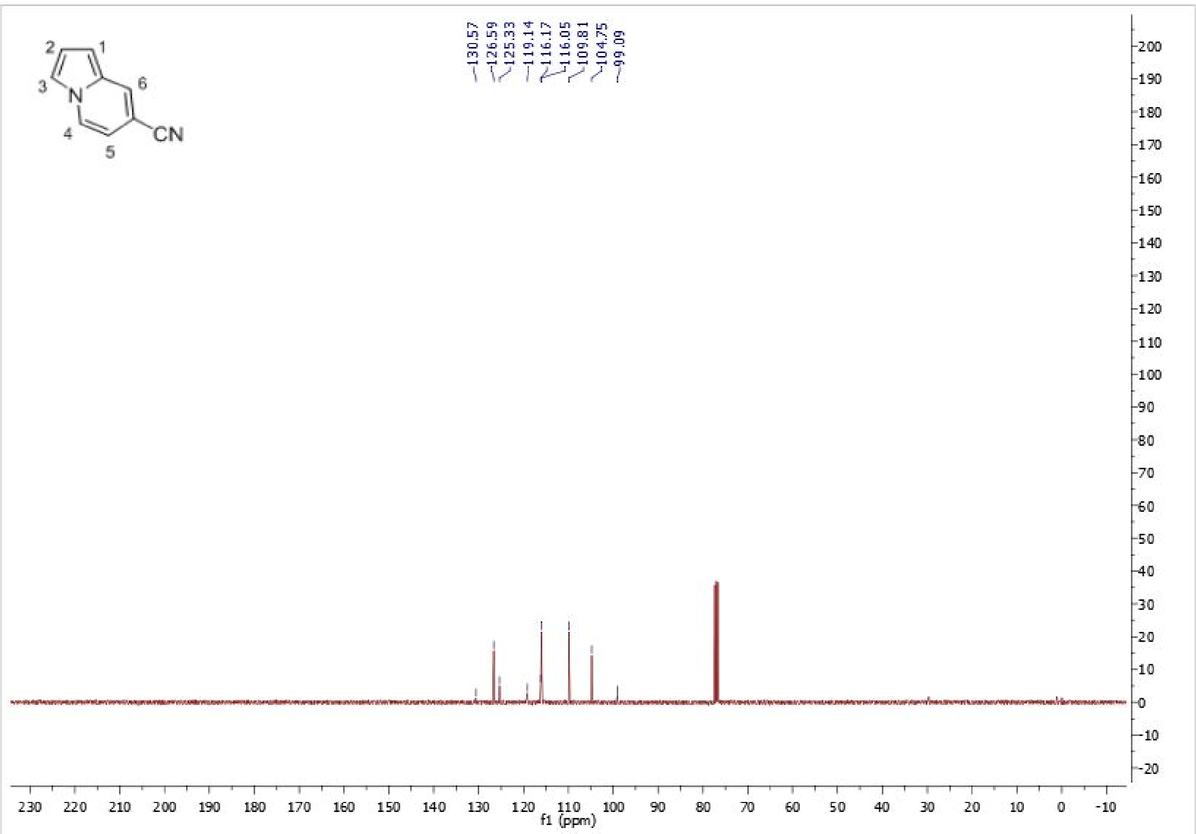

Figure S44.

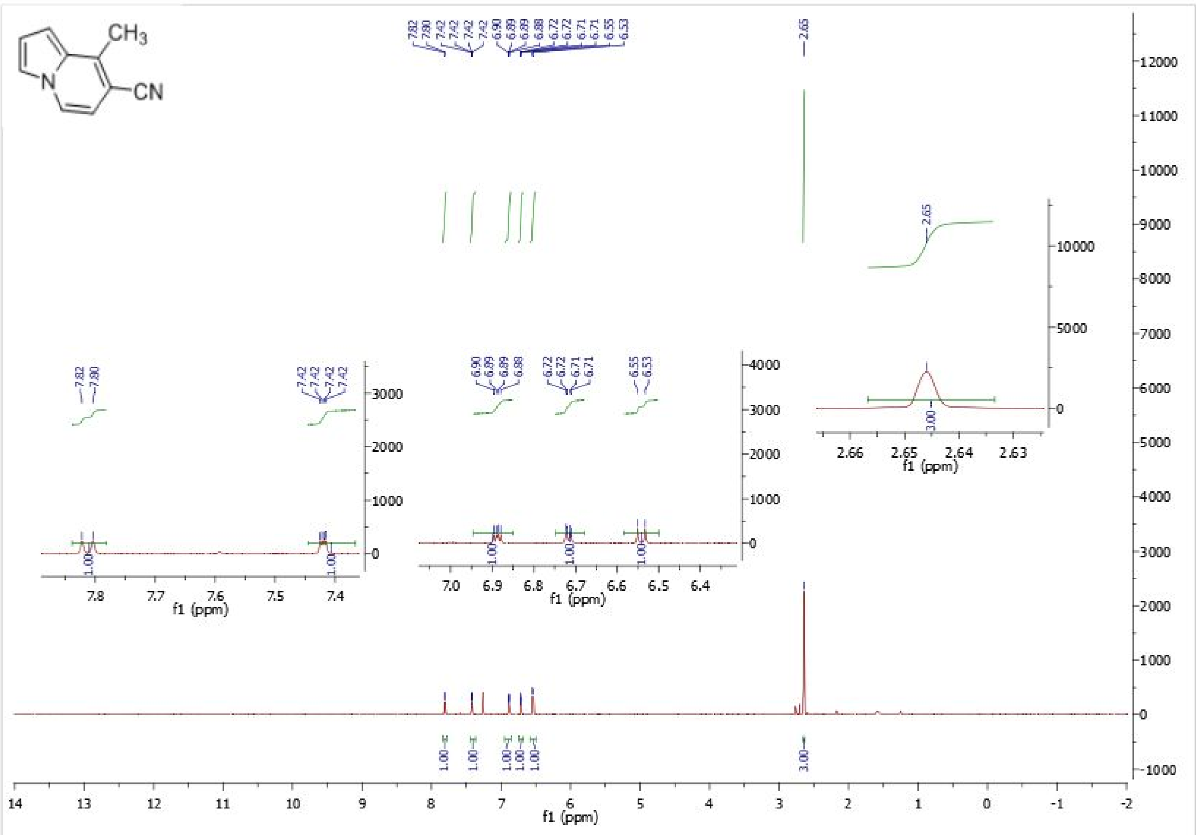

Figure S45.

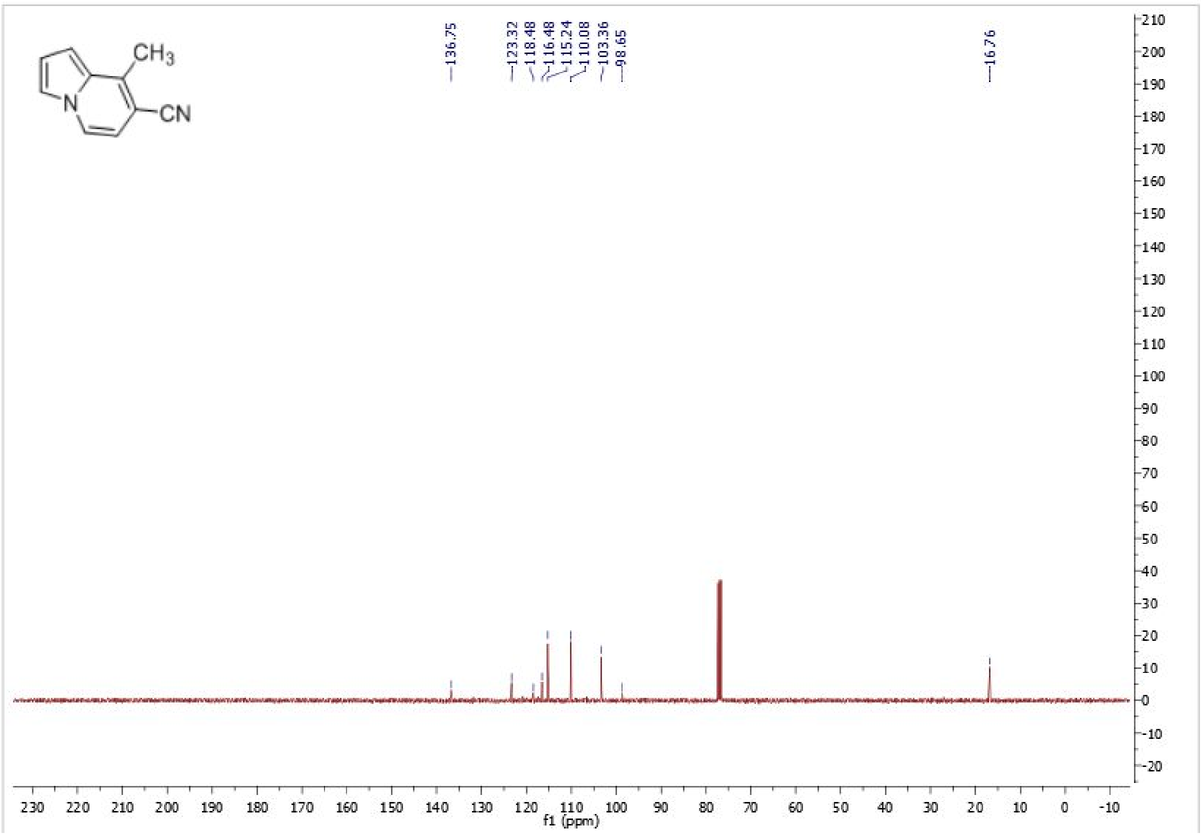

Figure S46

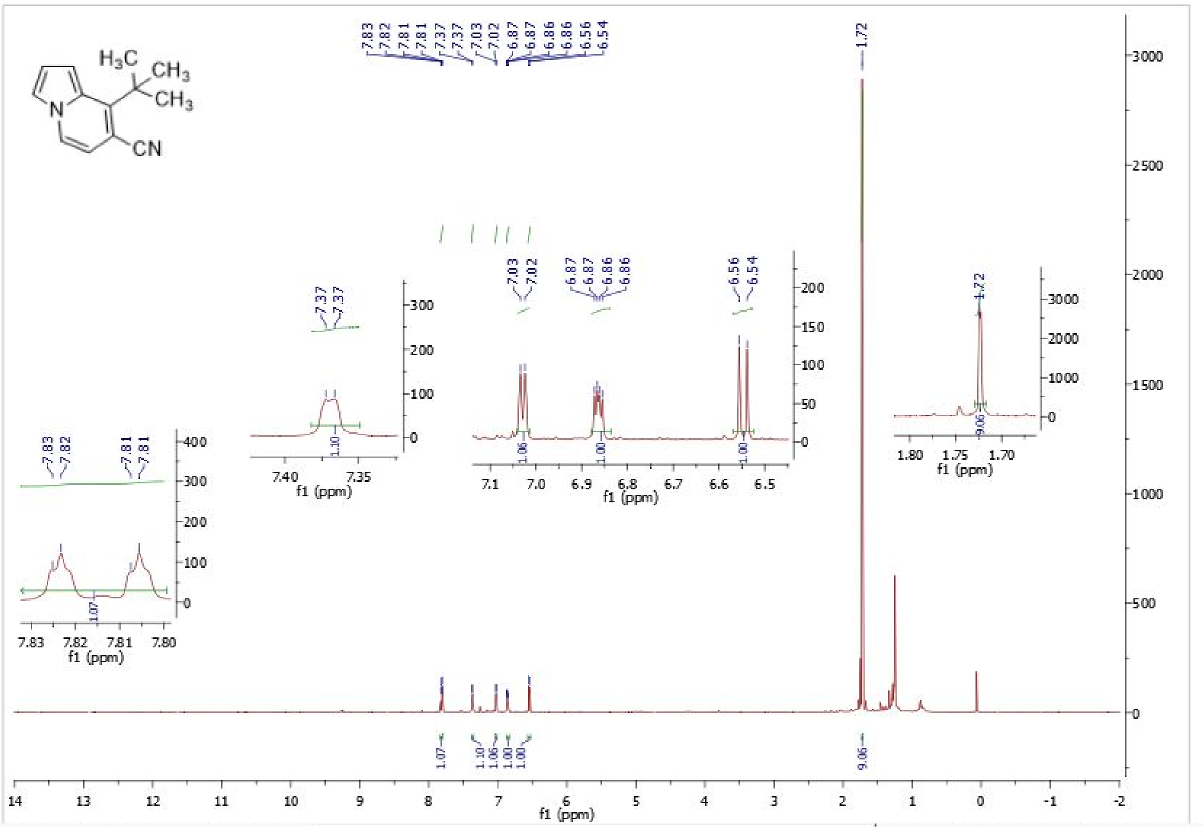

Figure S47.

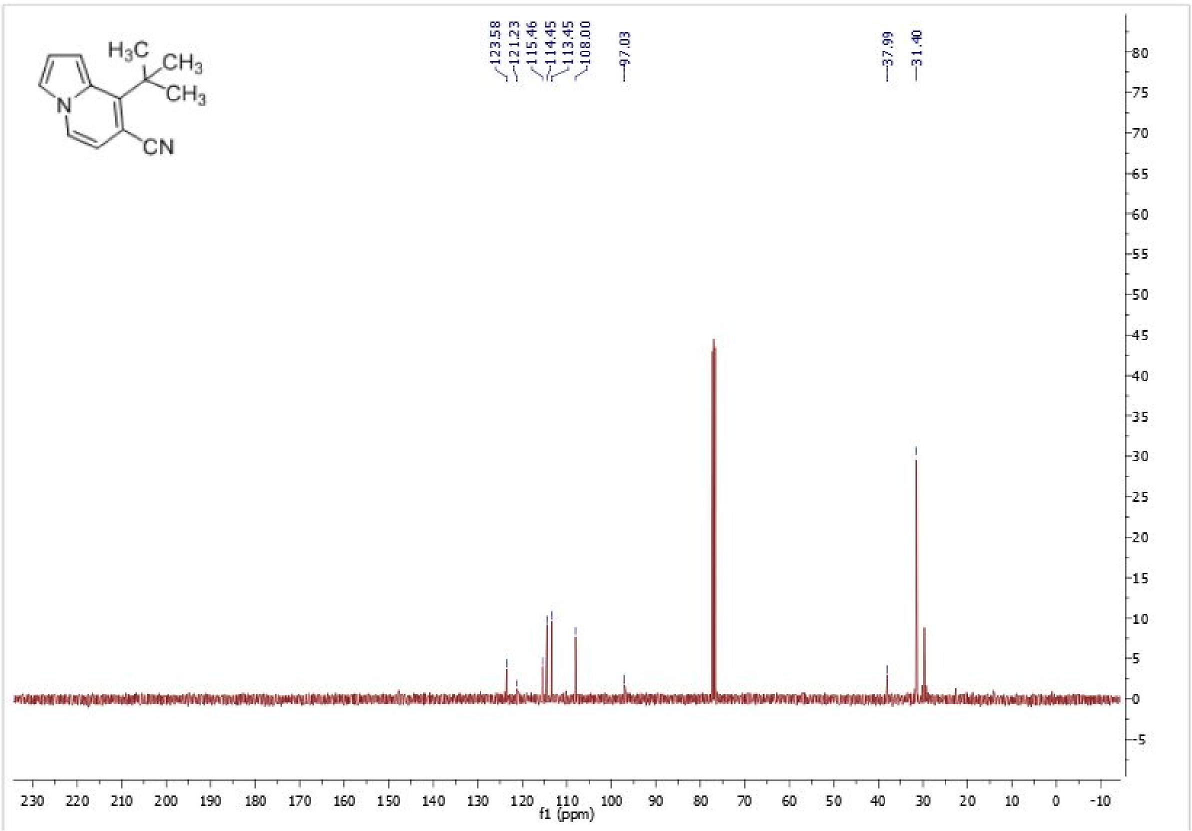

Figure S48.

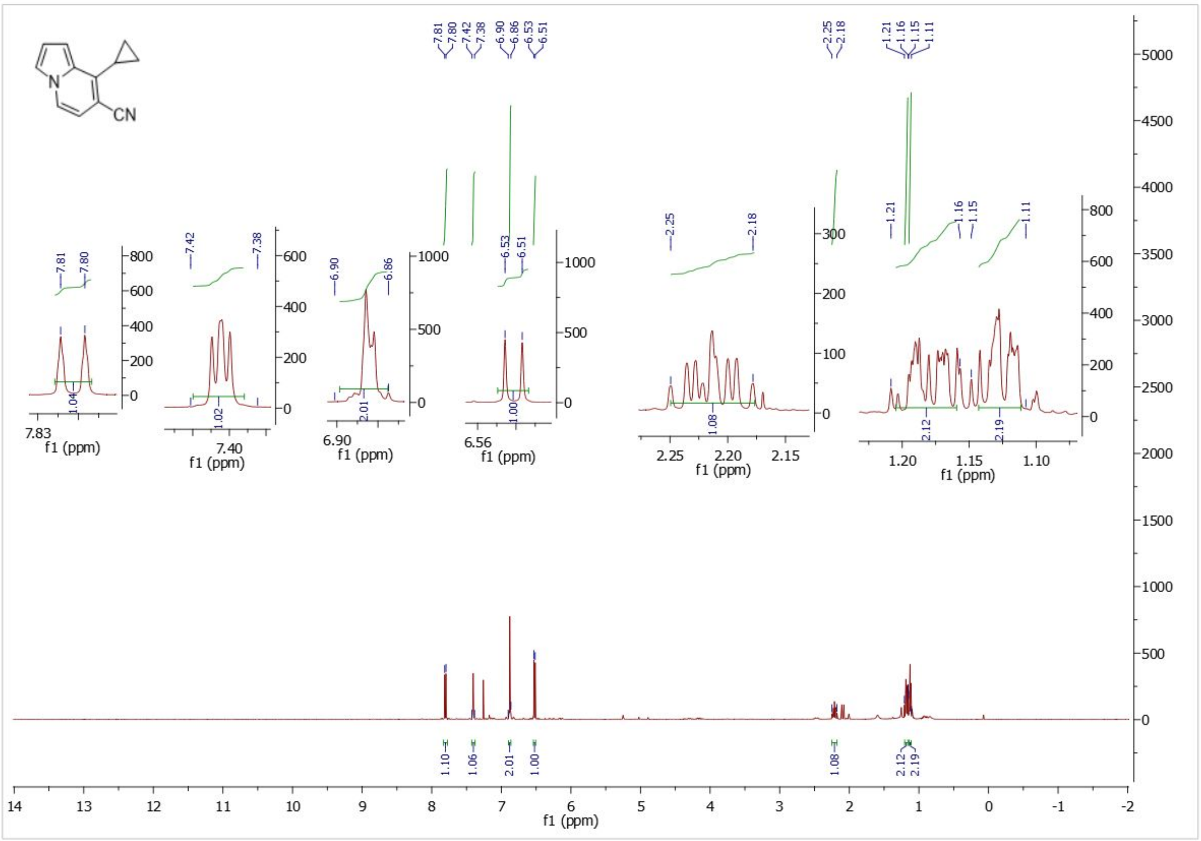

Figure S49.

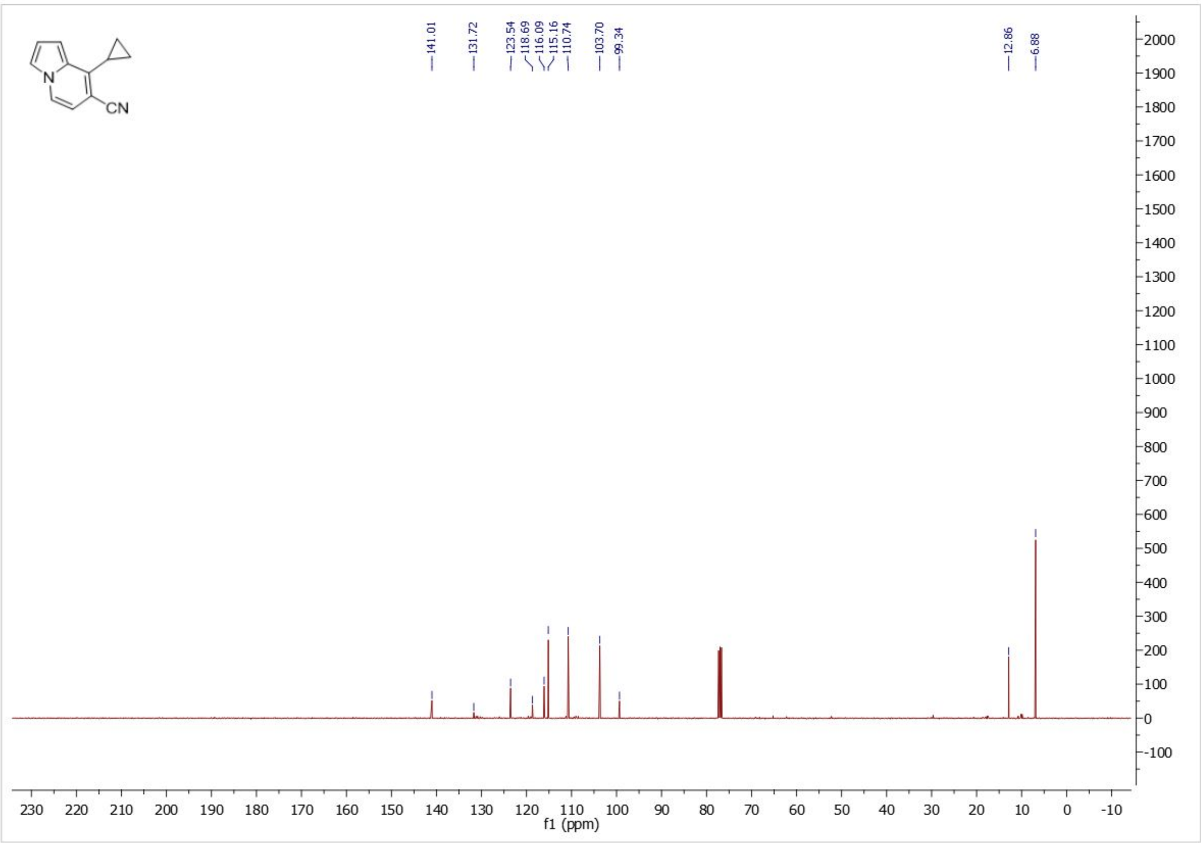

Figure S50.

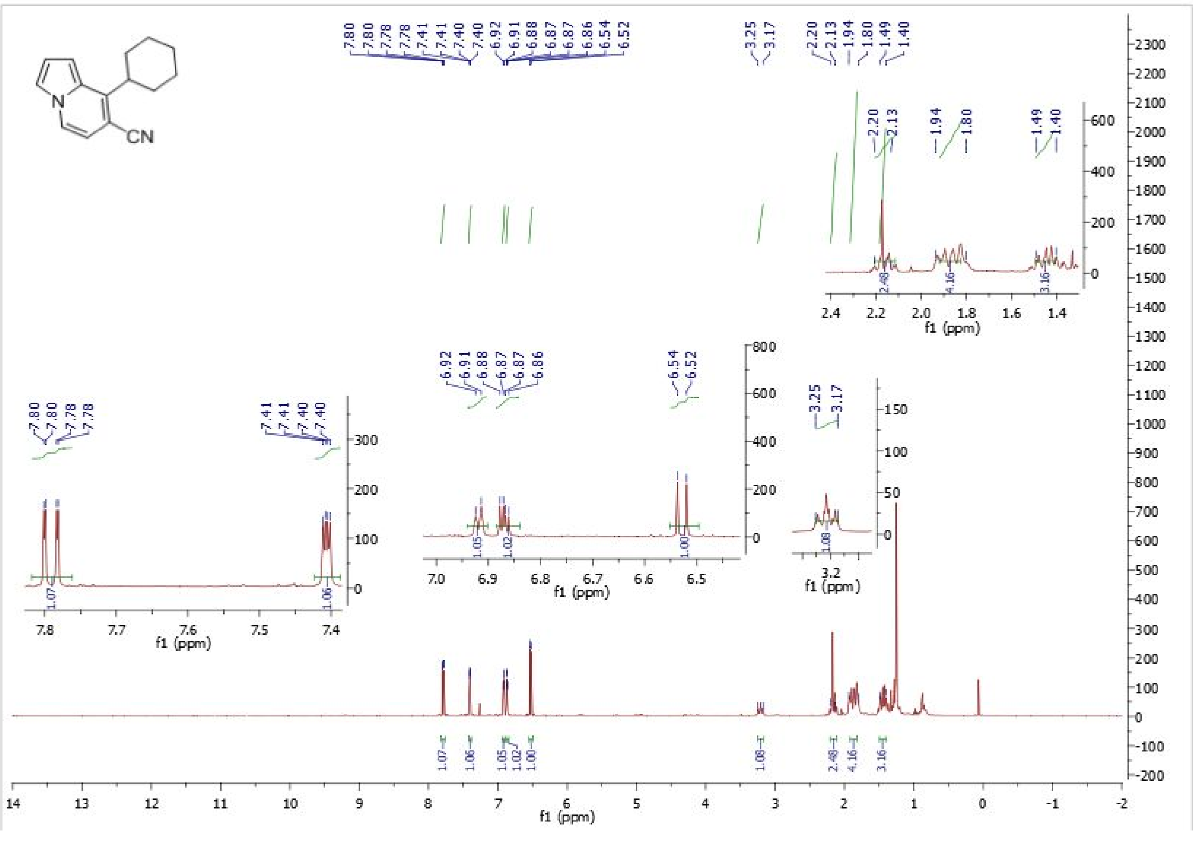

Figure S51.

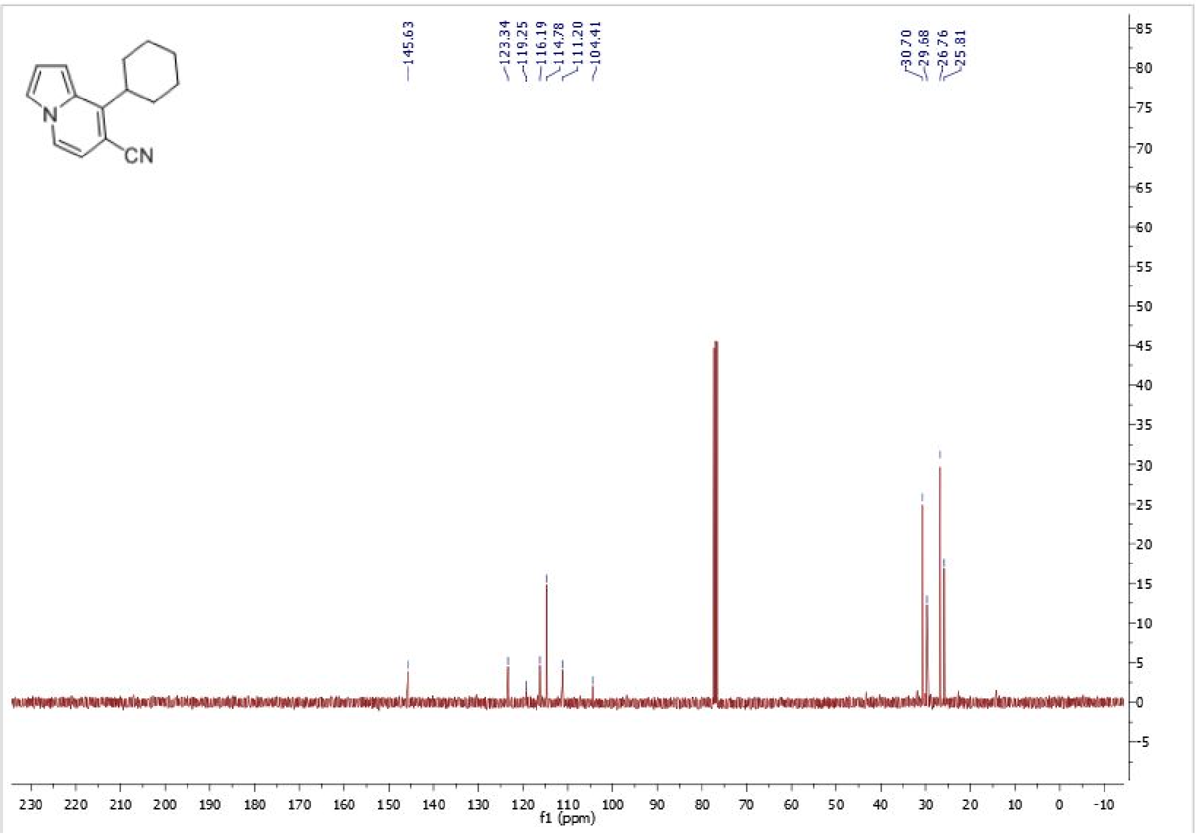

Figure S52.

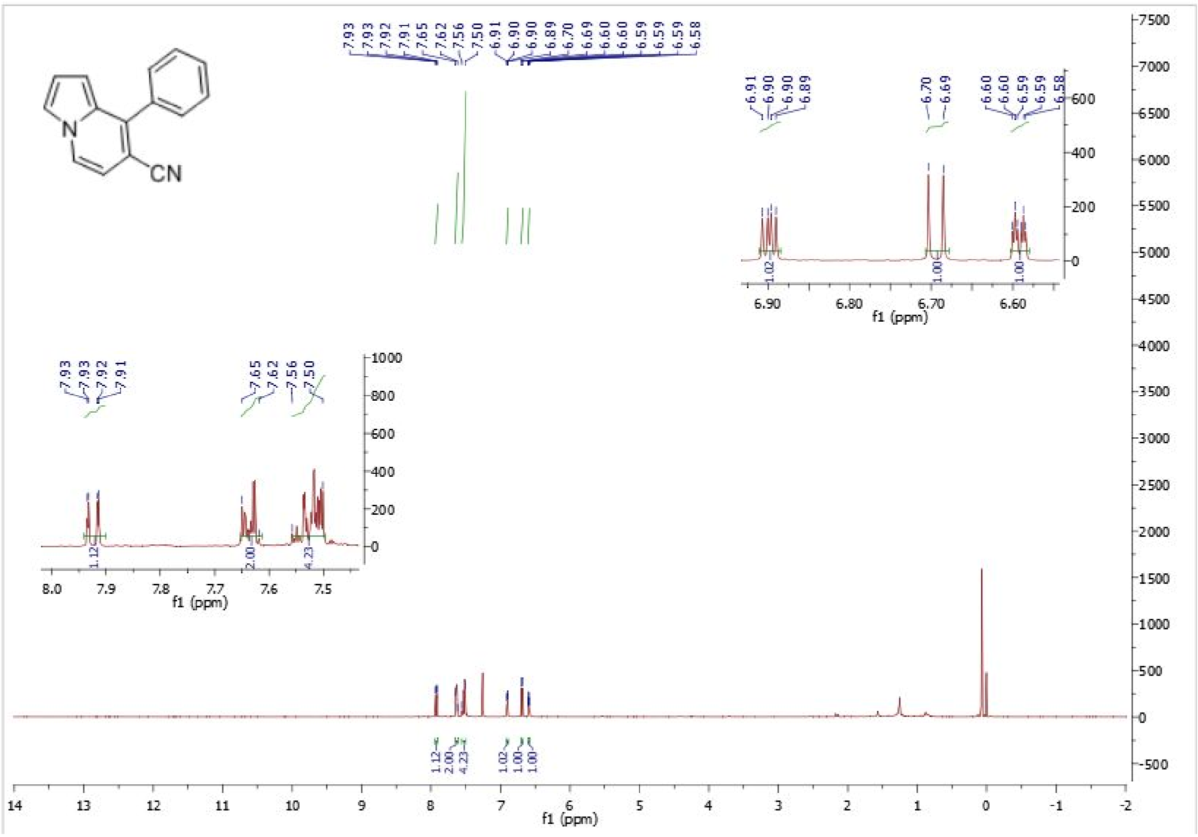

Figure S53.

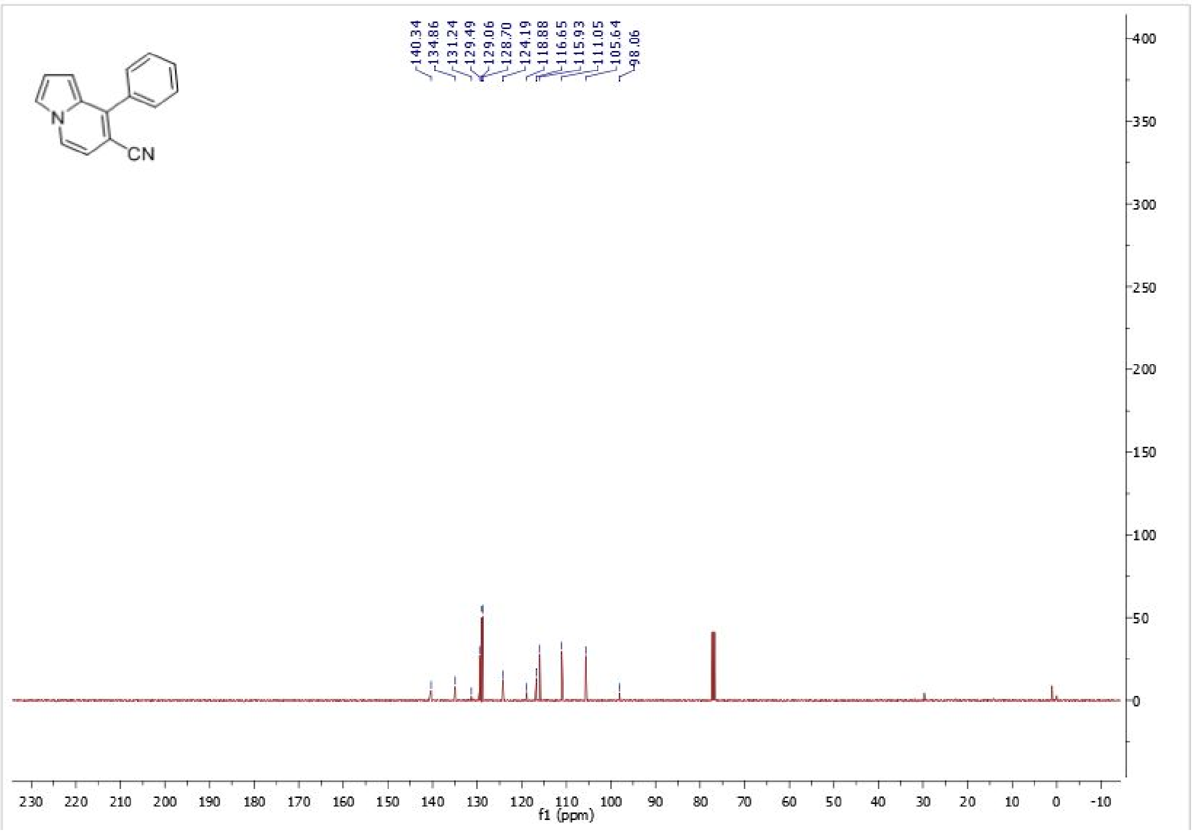

Figure S54.

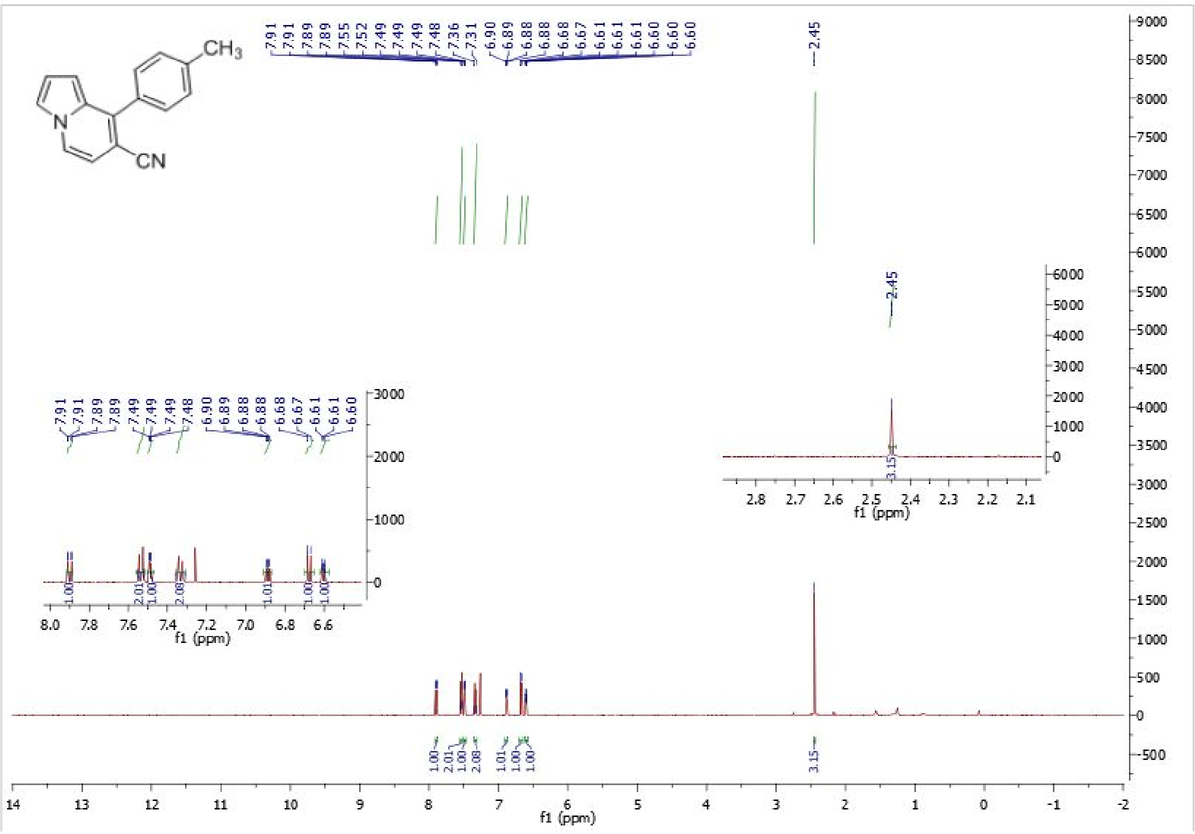

Figure S55.

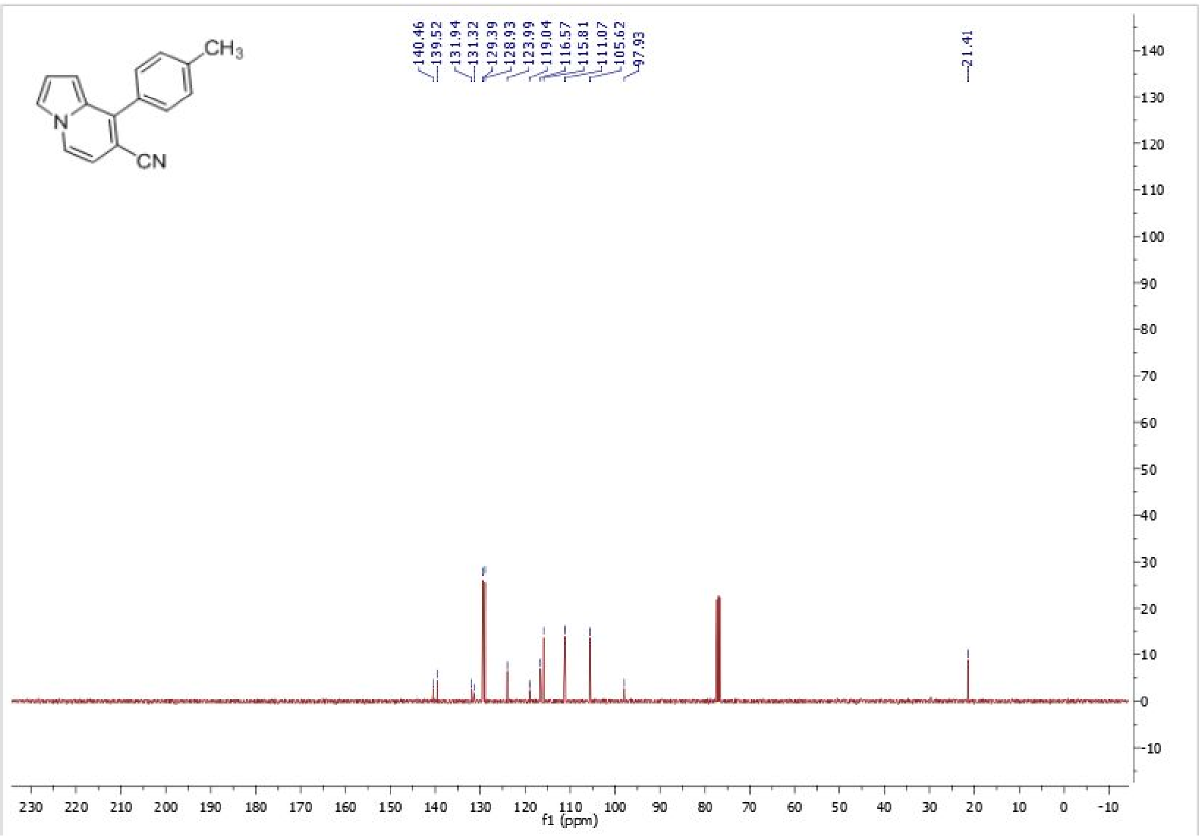

Figure S56.

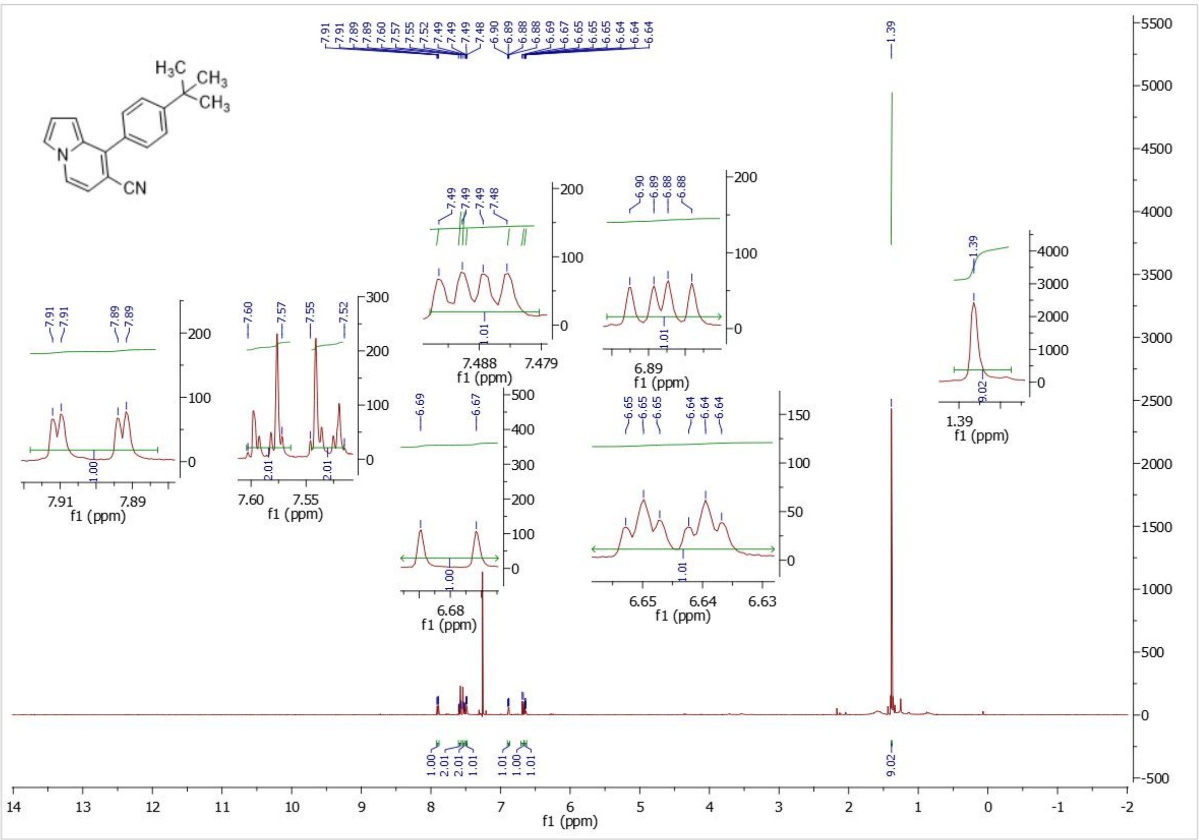

Figure S57.

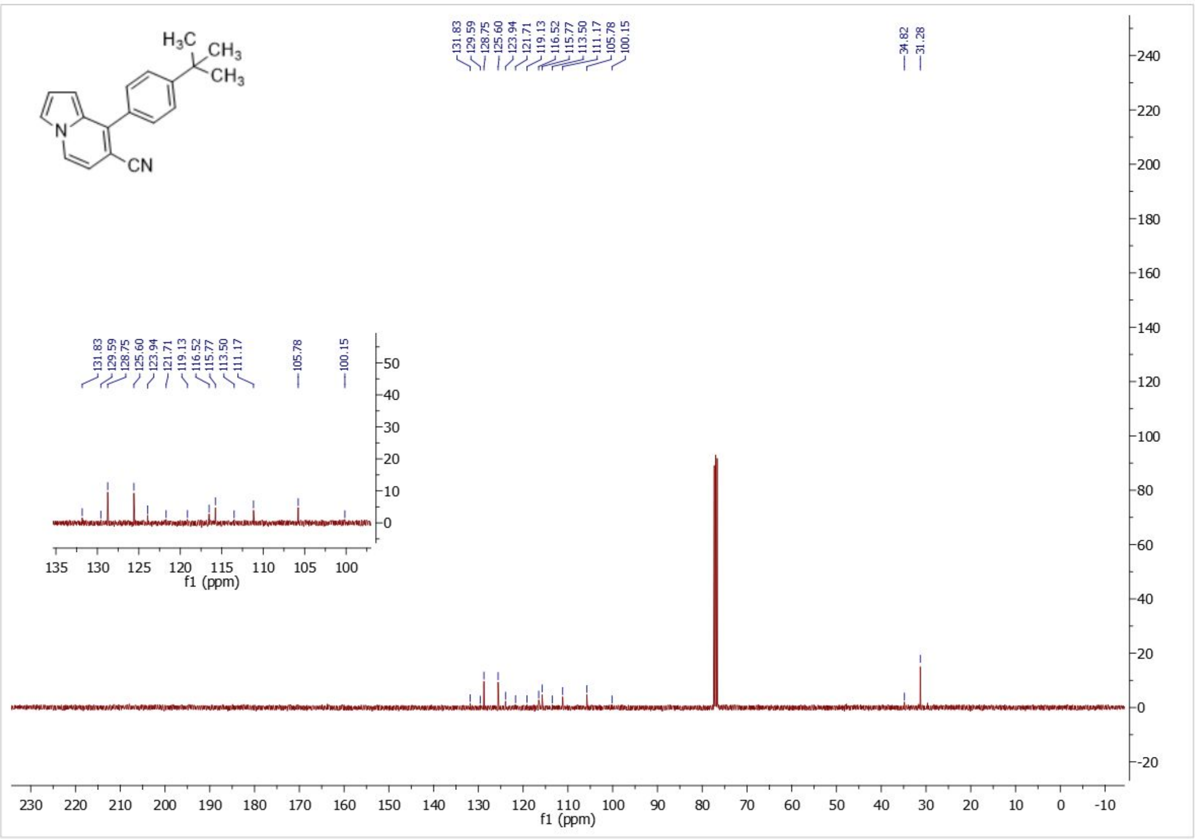

Figure S58.

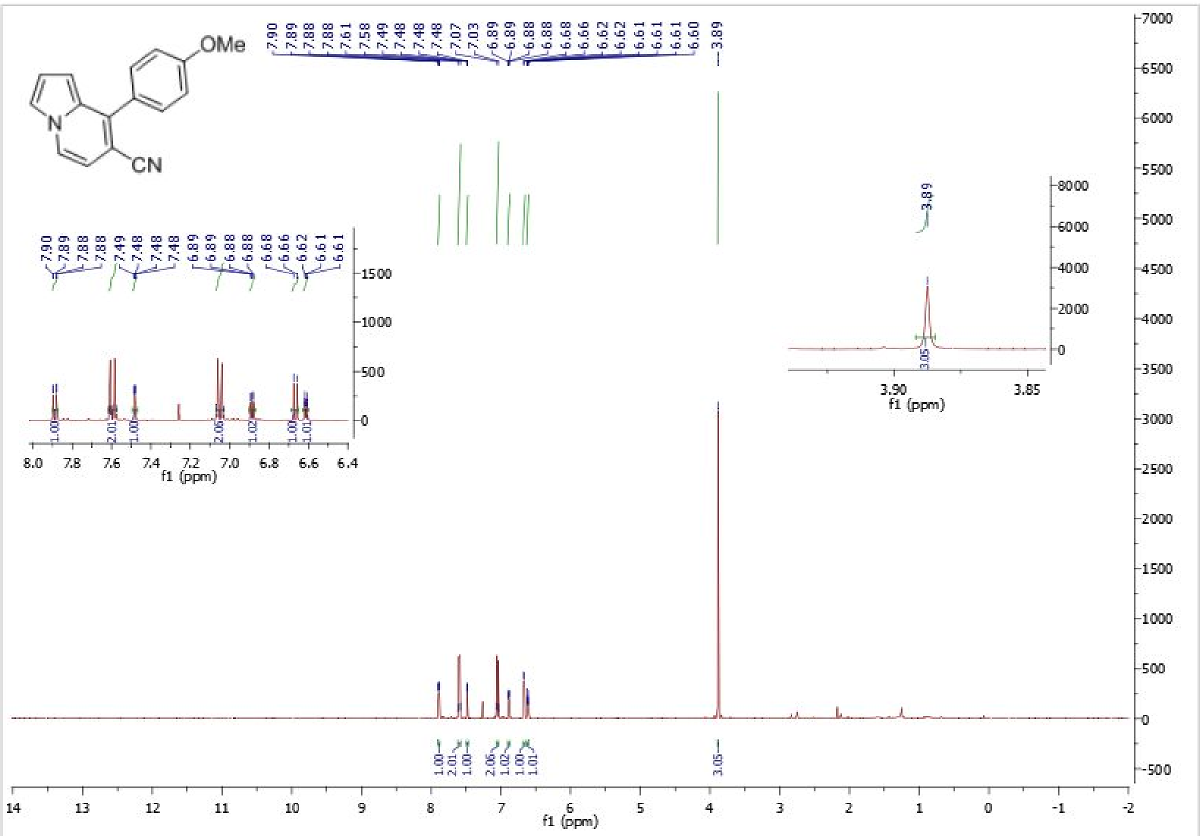

Figure S59.

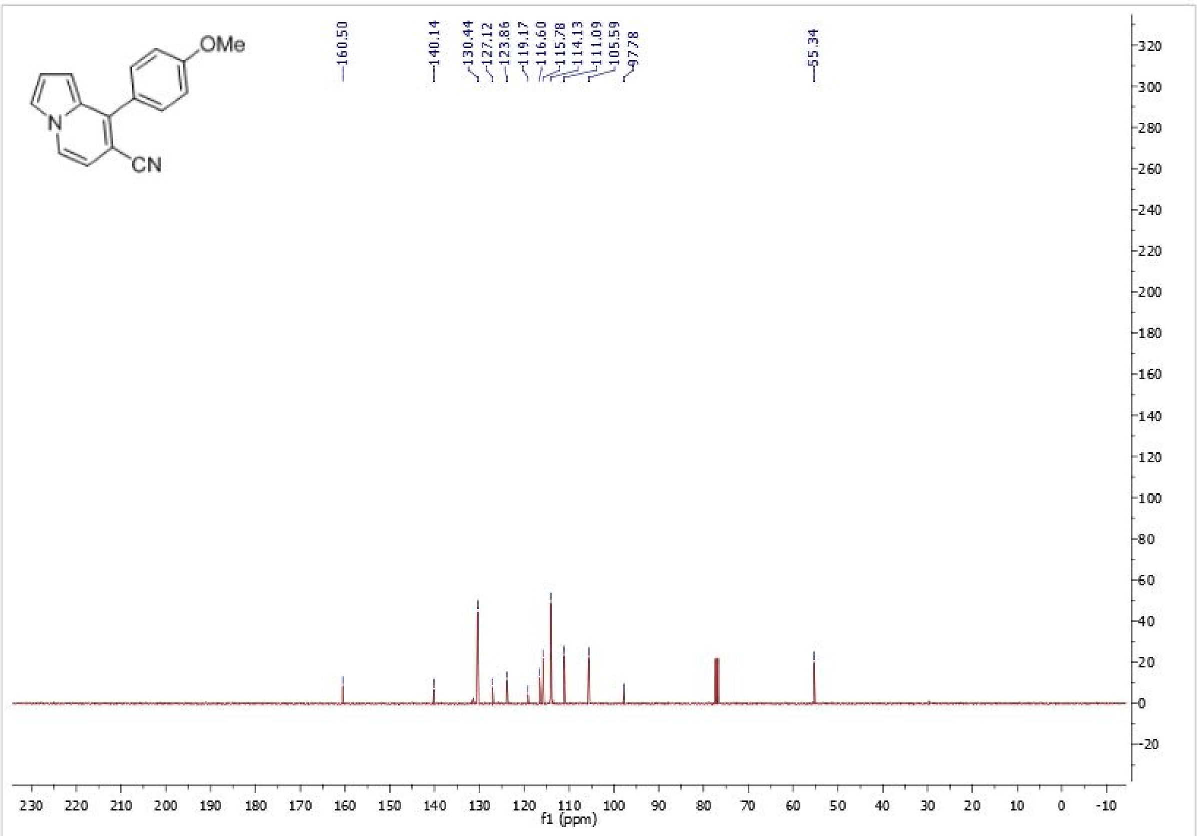

Figure S60.

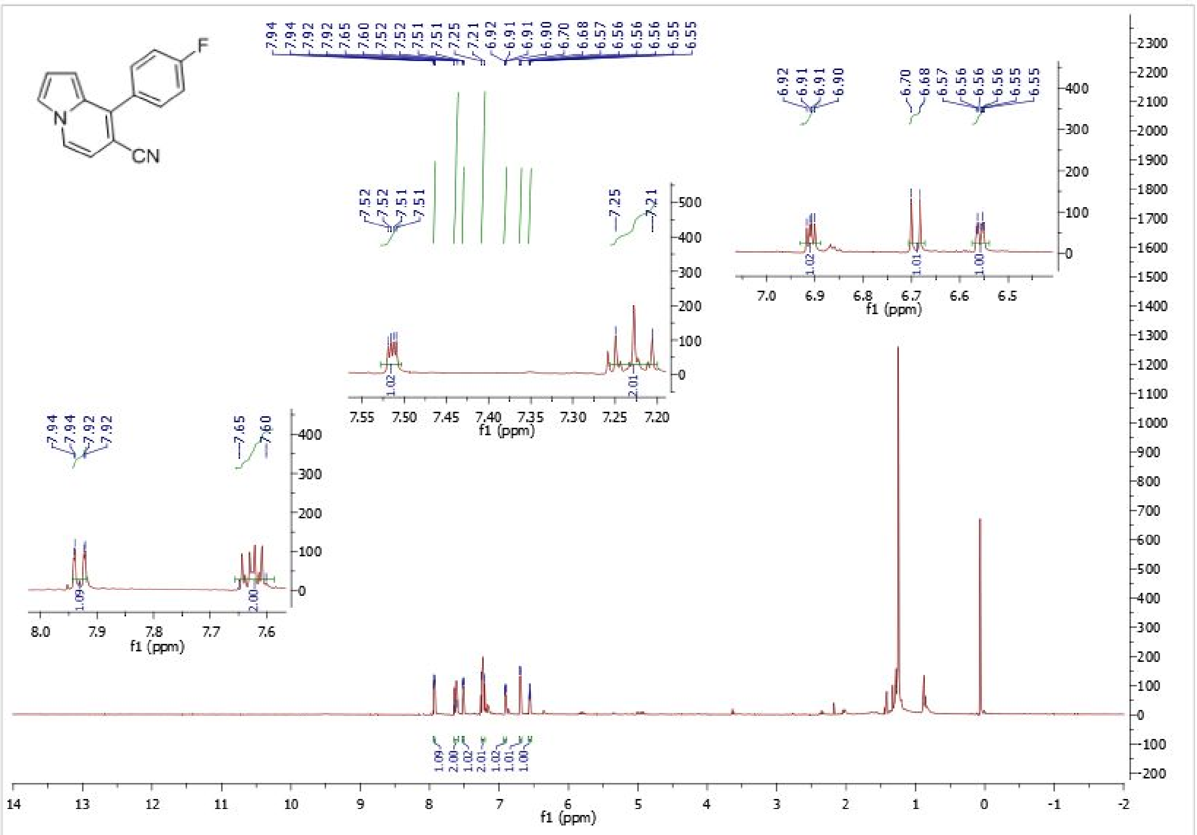

Figure S61.

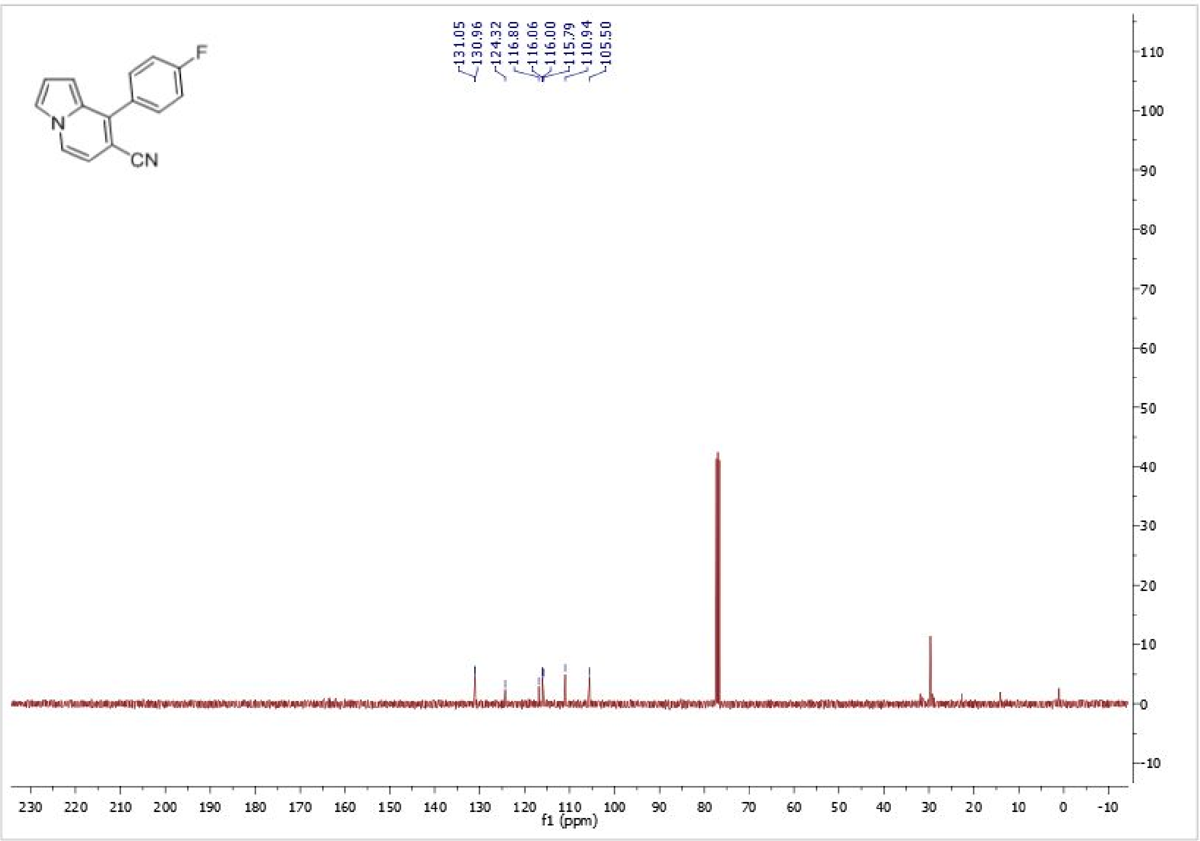

Figure S62.

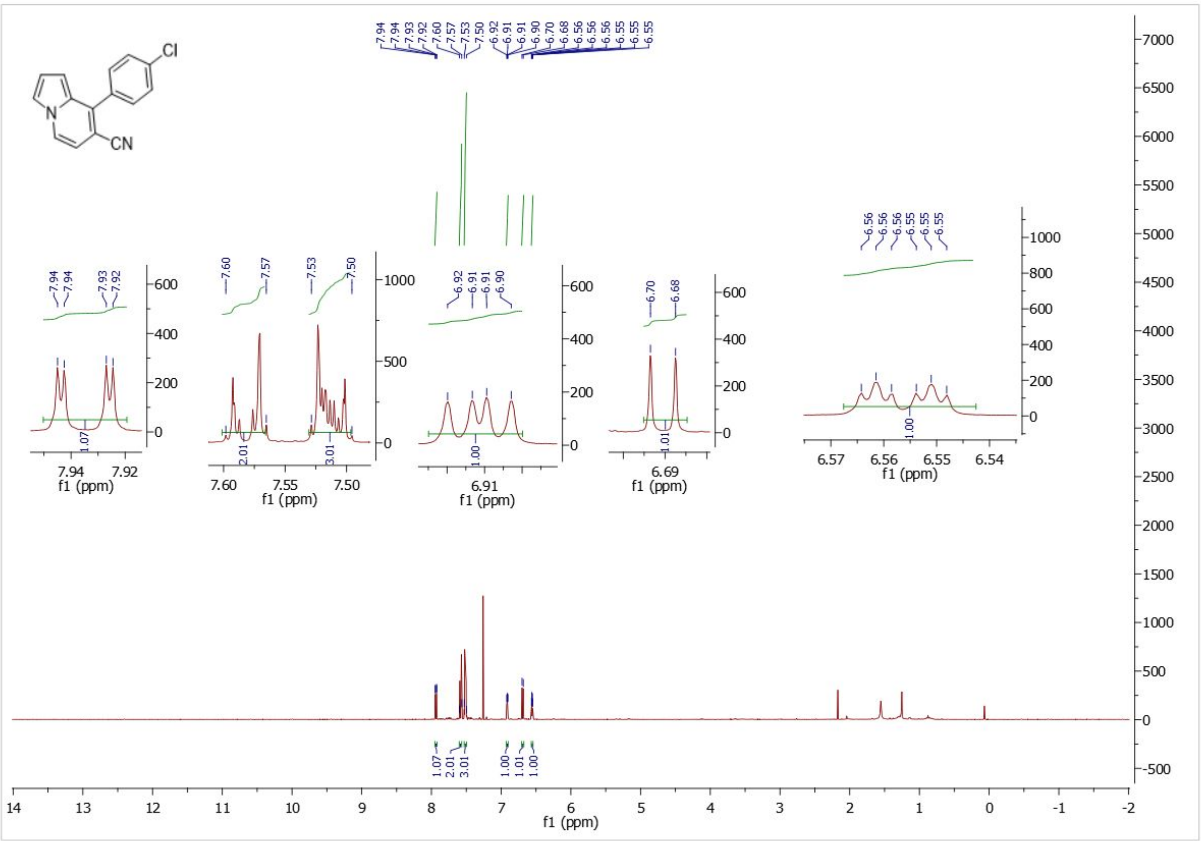

Figure S63.

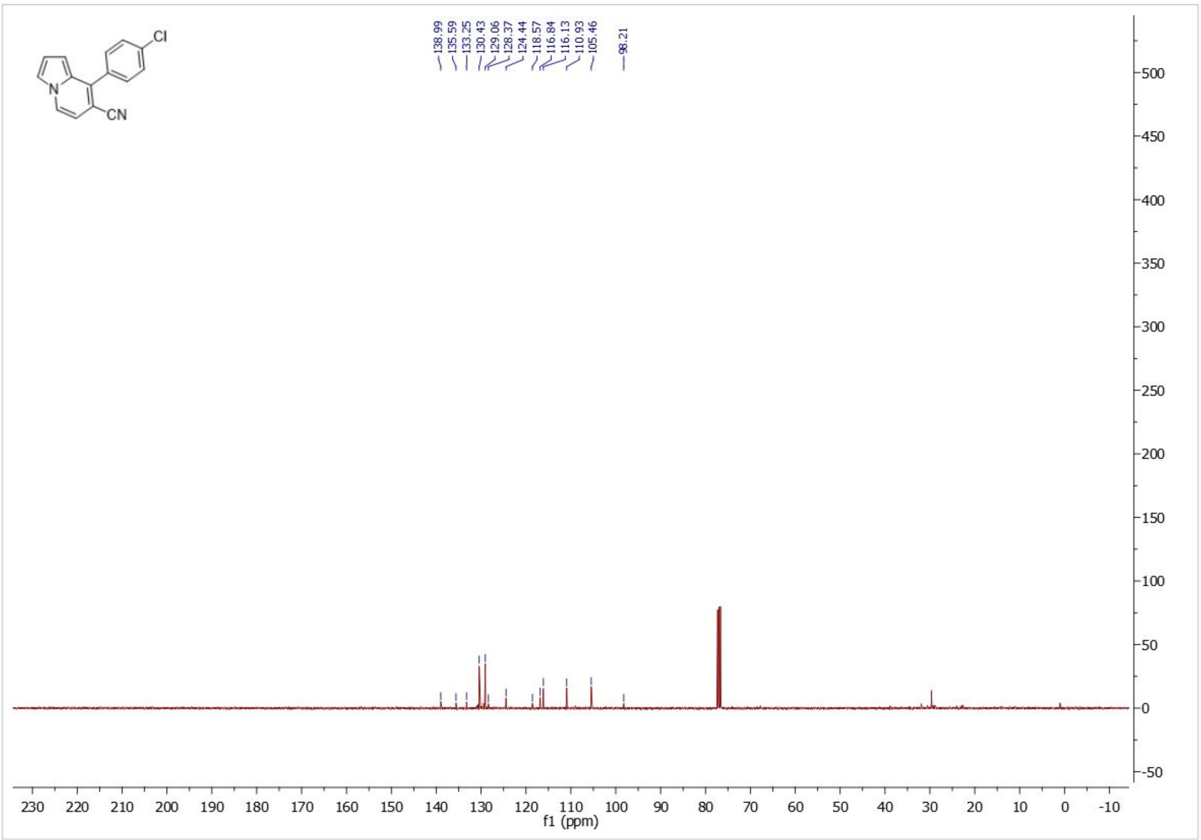

Figure S64.

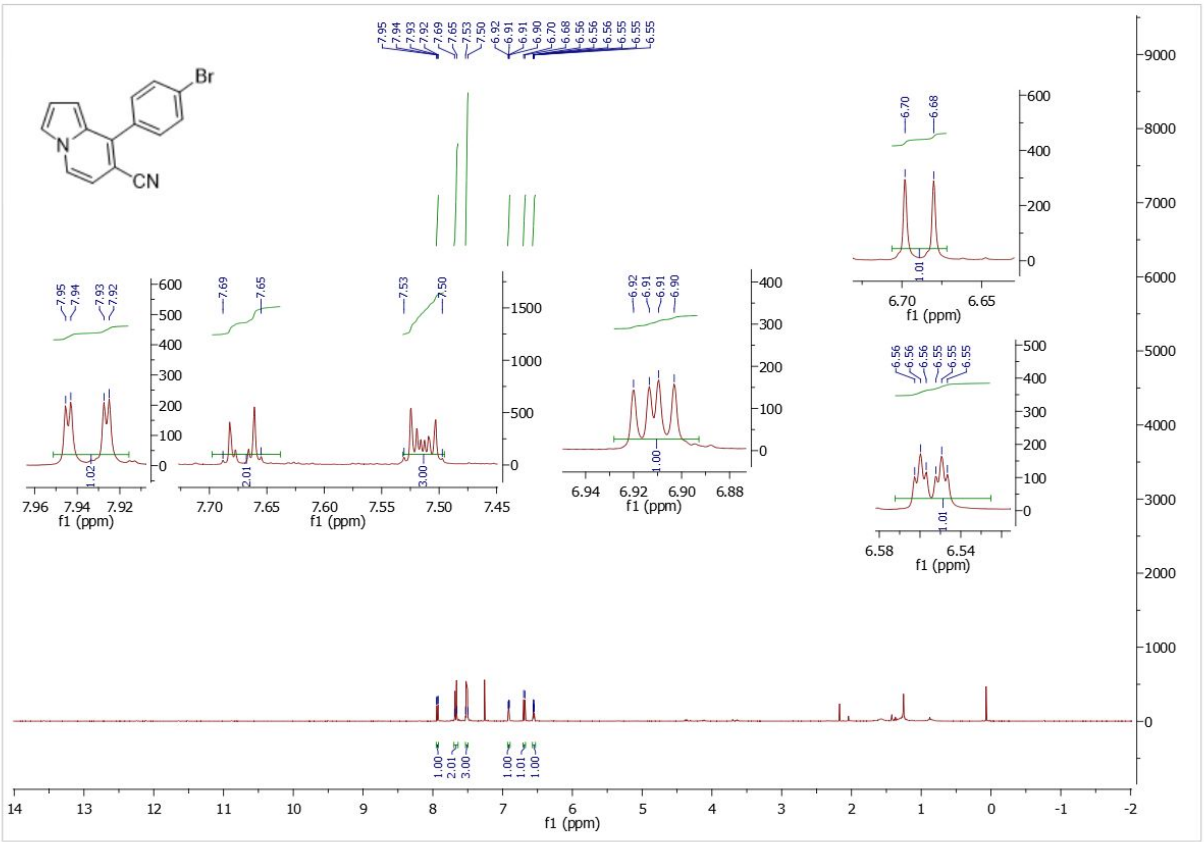

Figure S65.

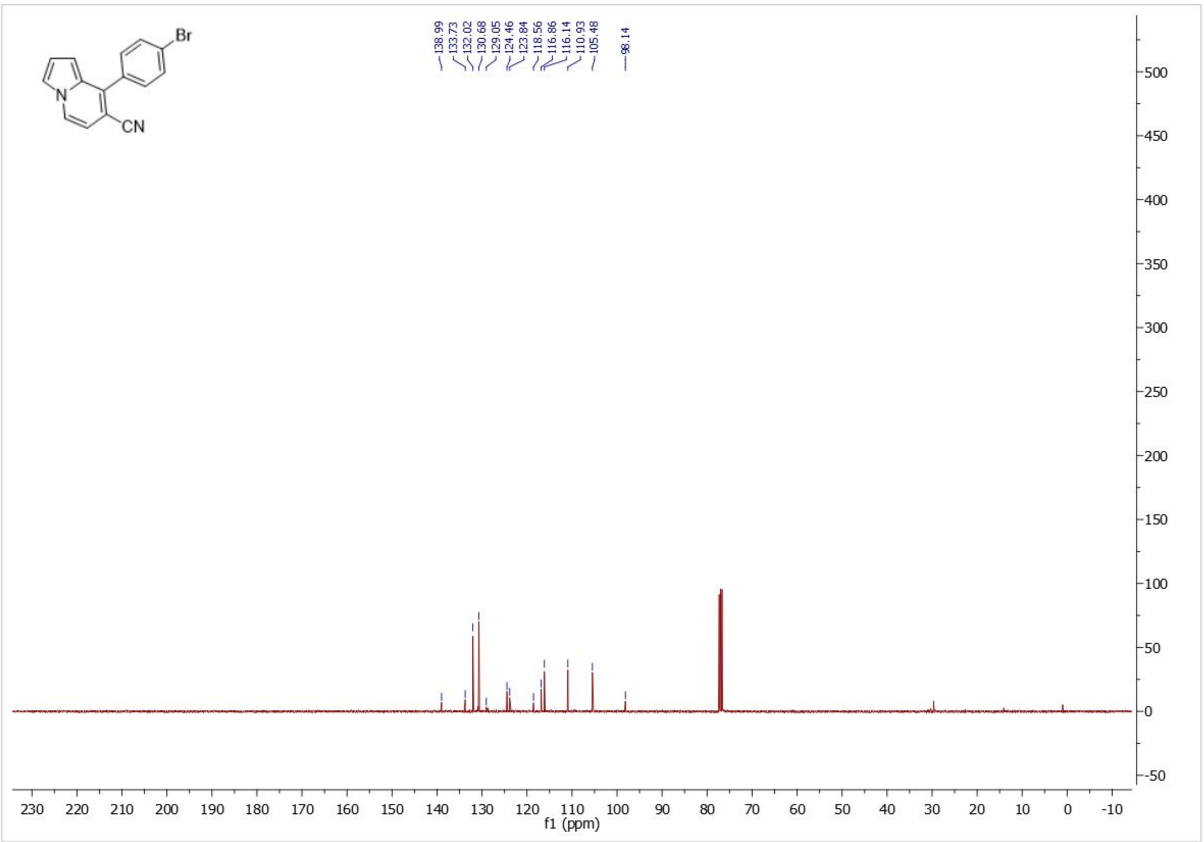

Figure S66

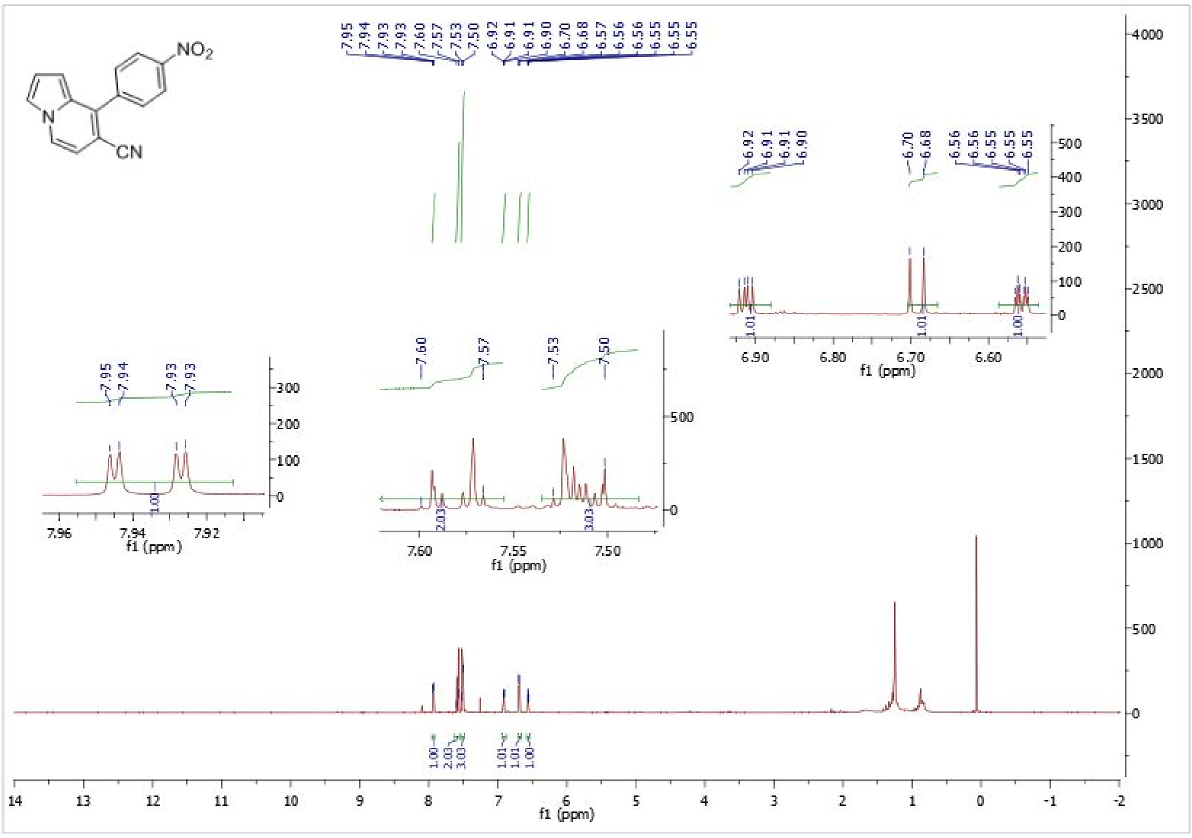

Figure S67.

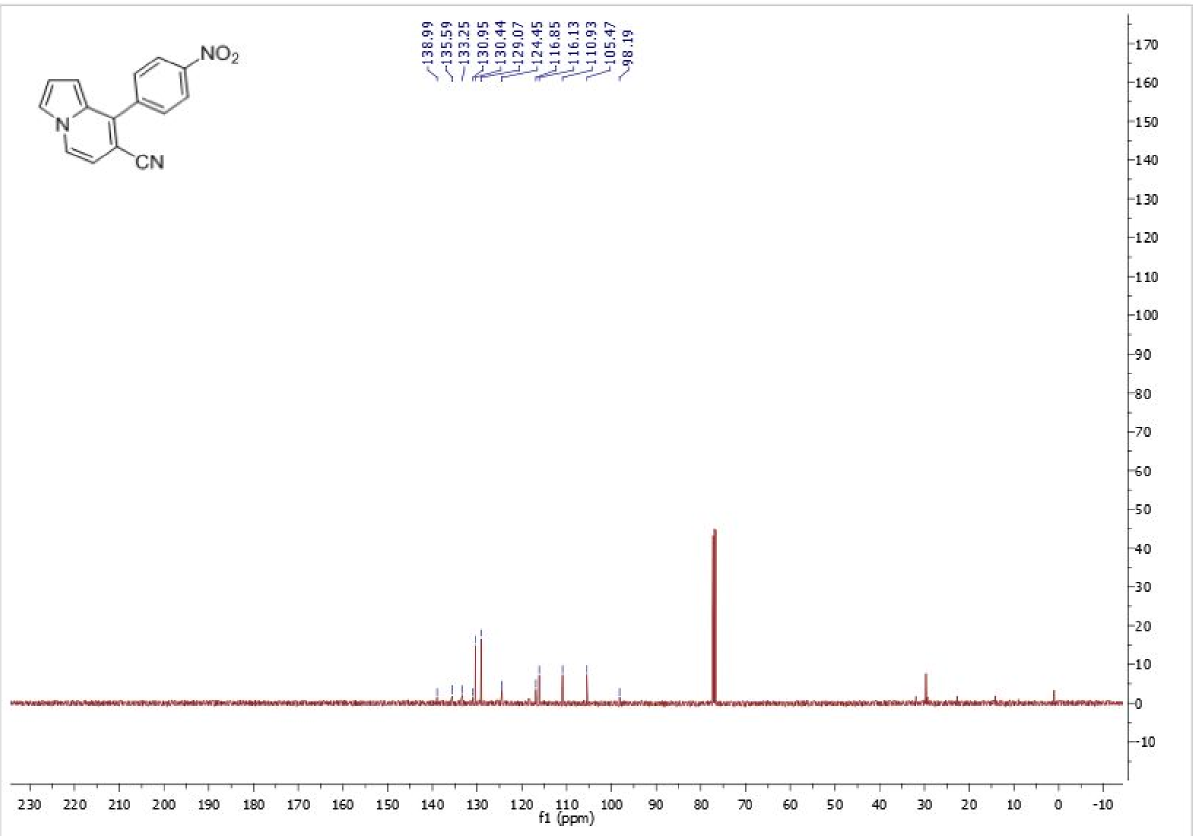

Figure S68.

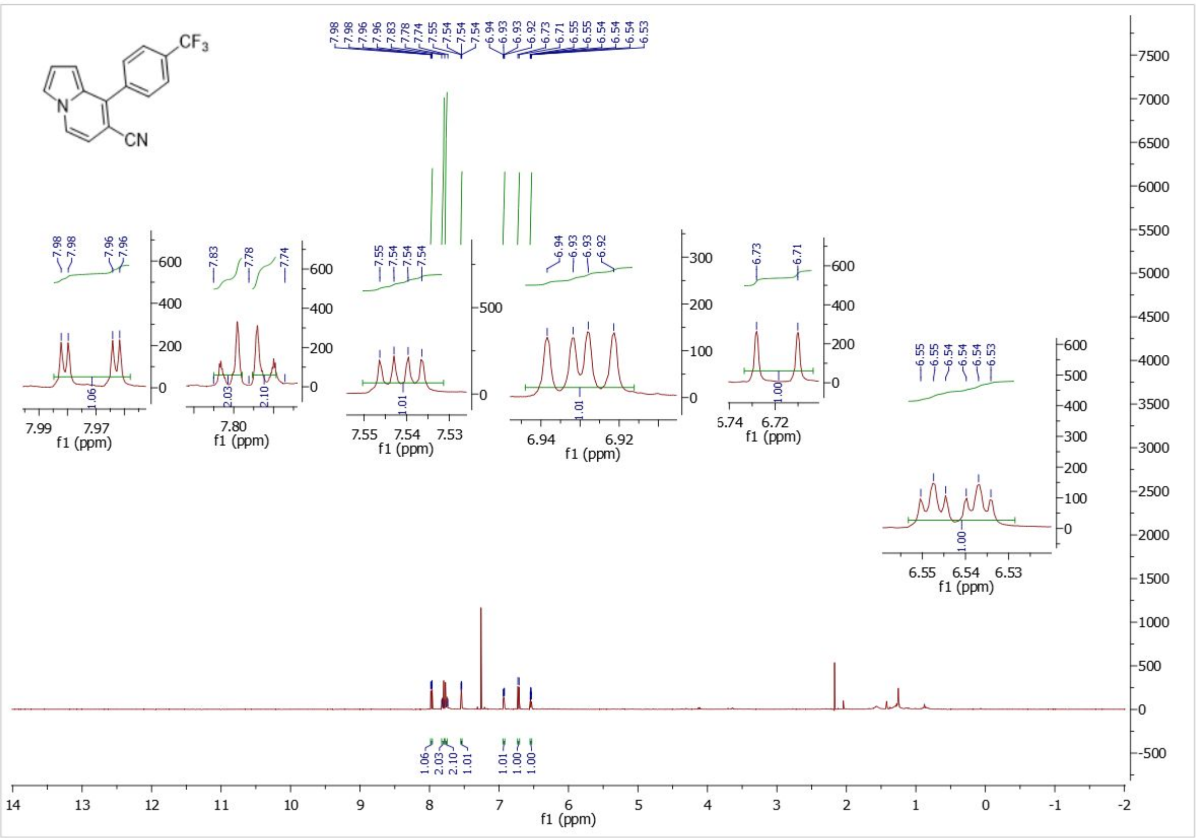

Figure S69.

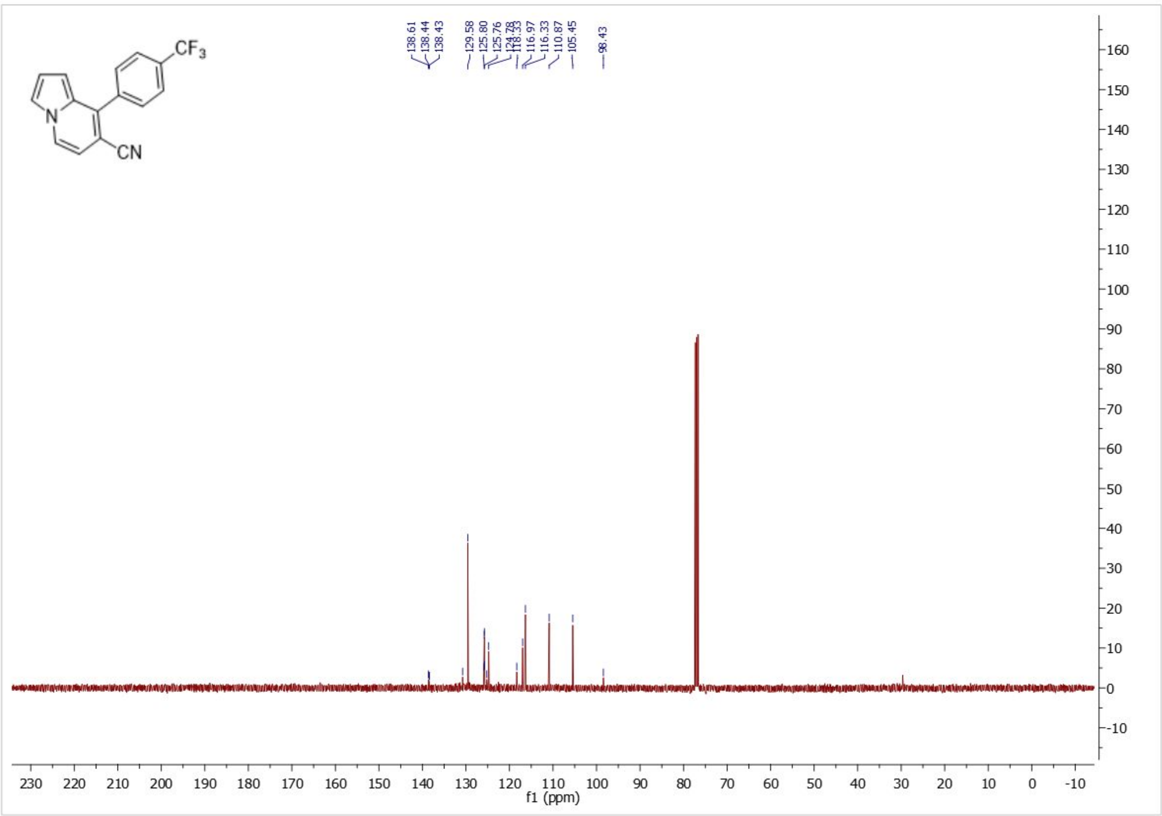

Figure S70.

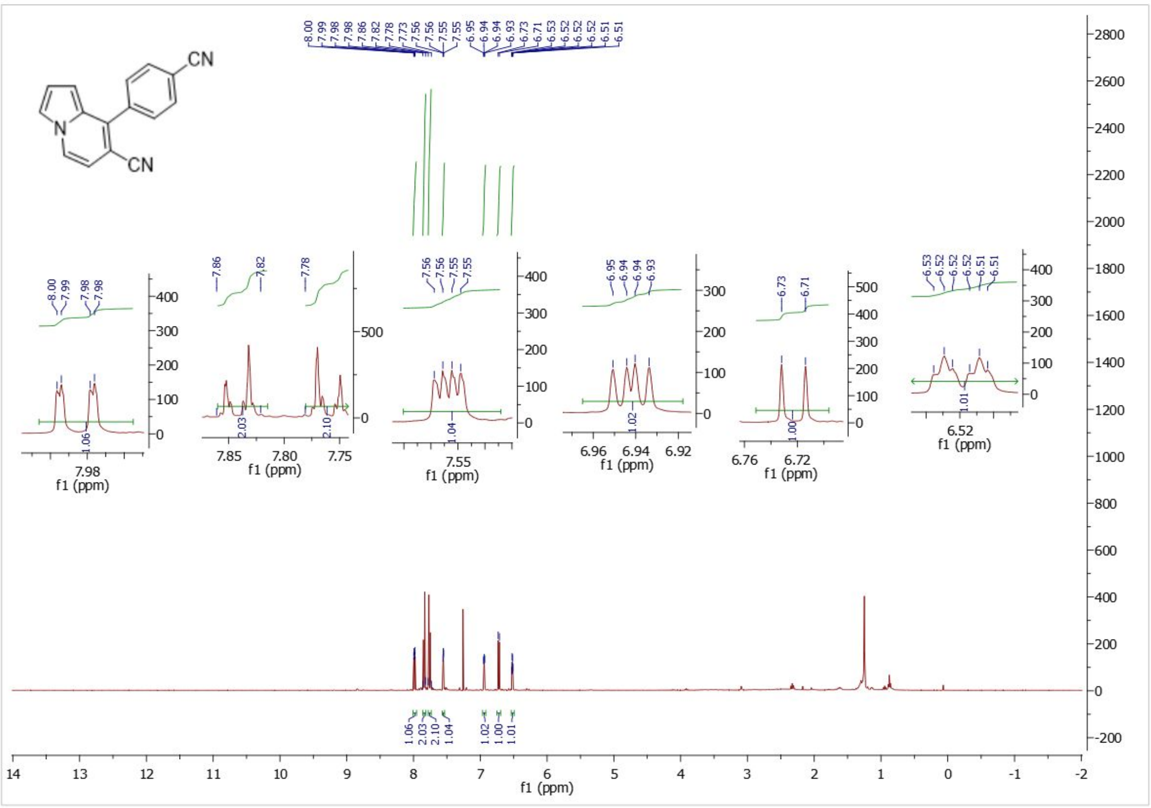

Figure S71.

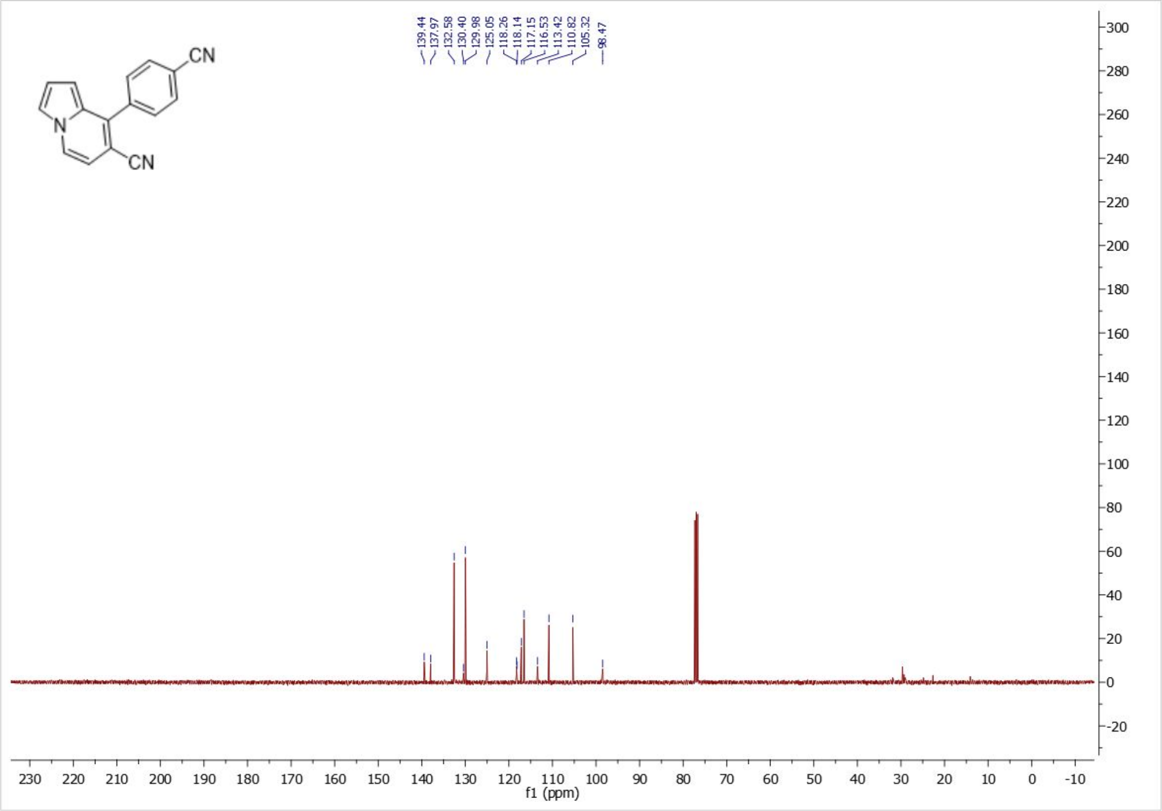

Figure S72.

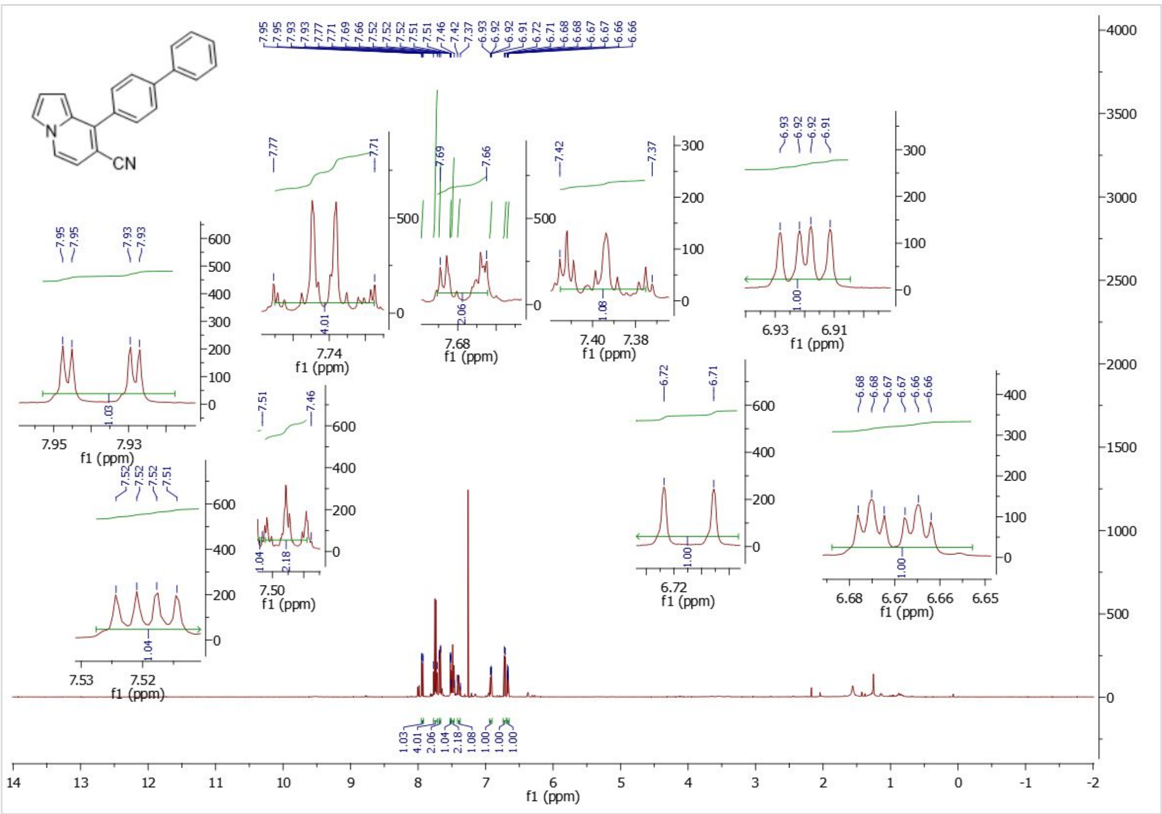

Figure S73.

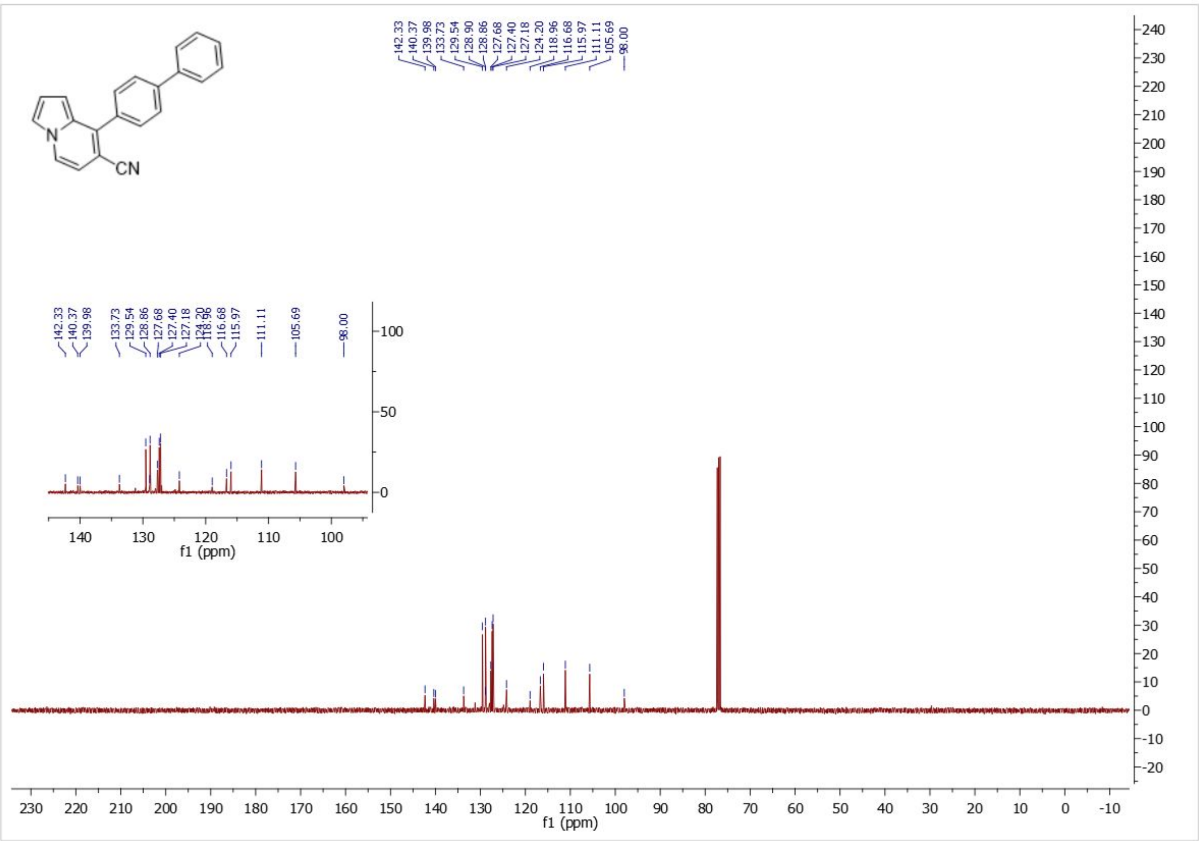

Figure S74.

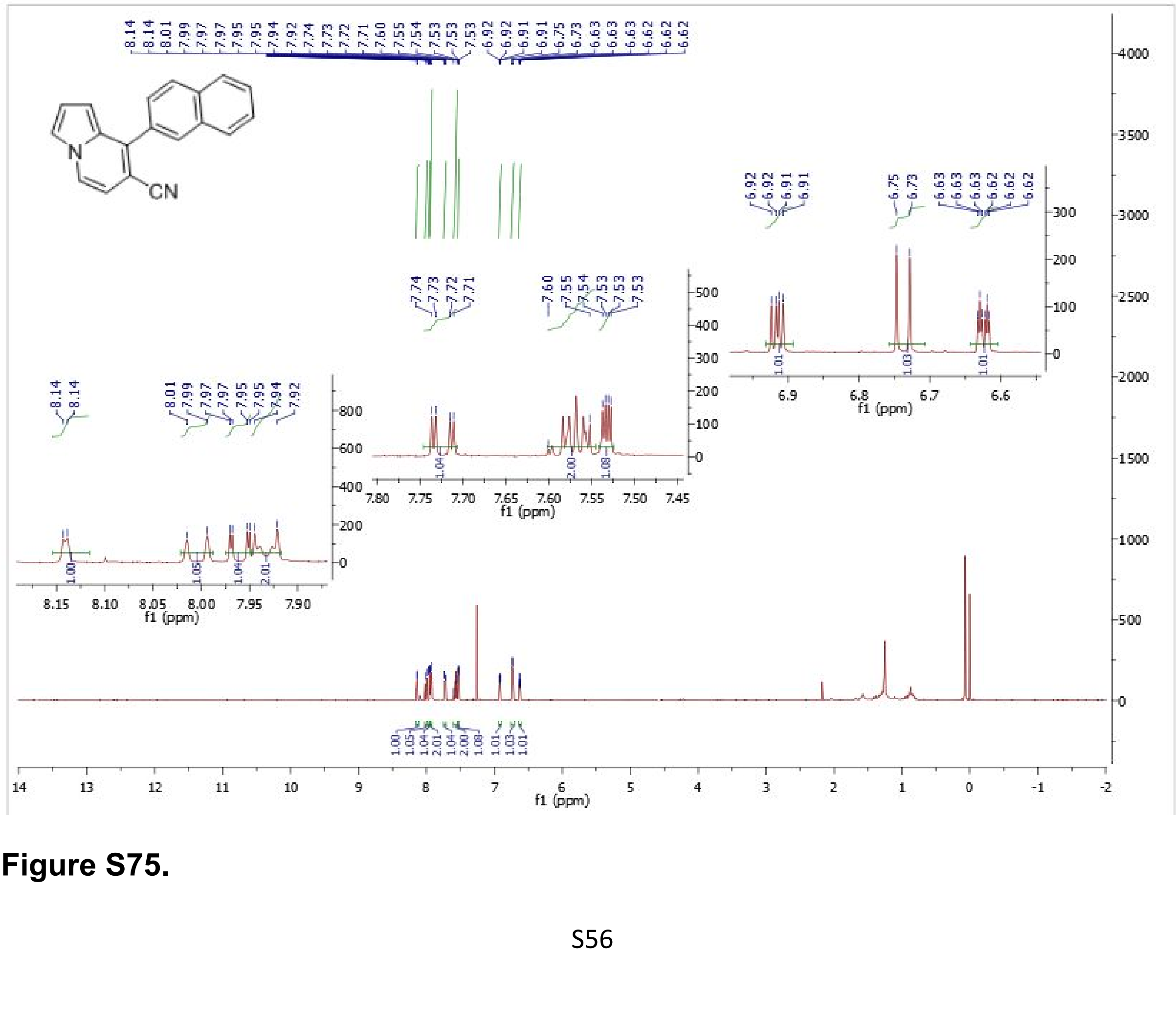

Figure S75.

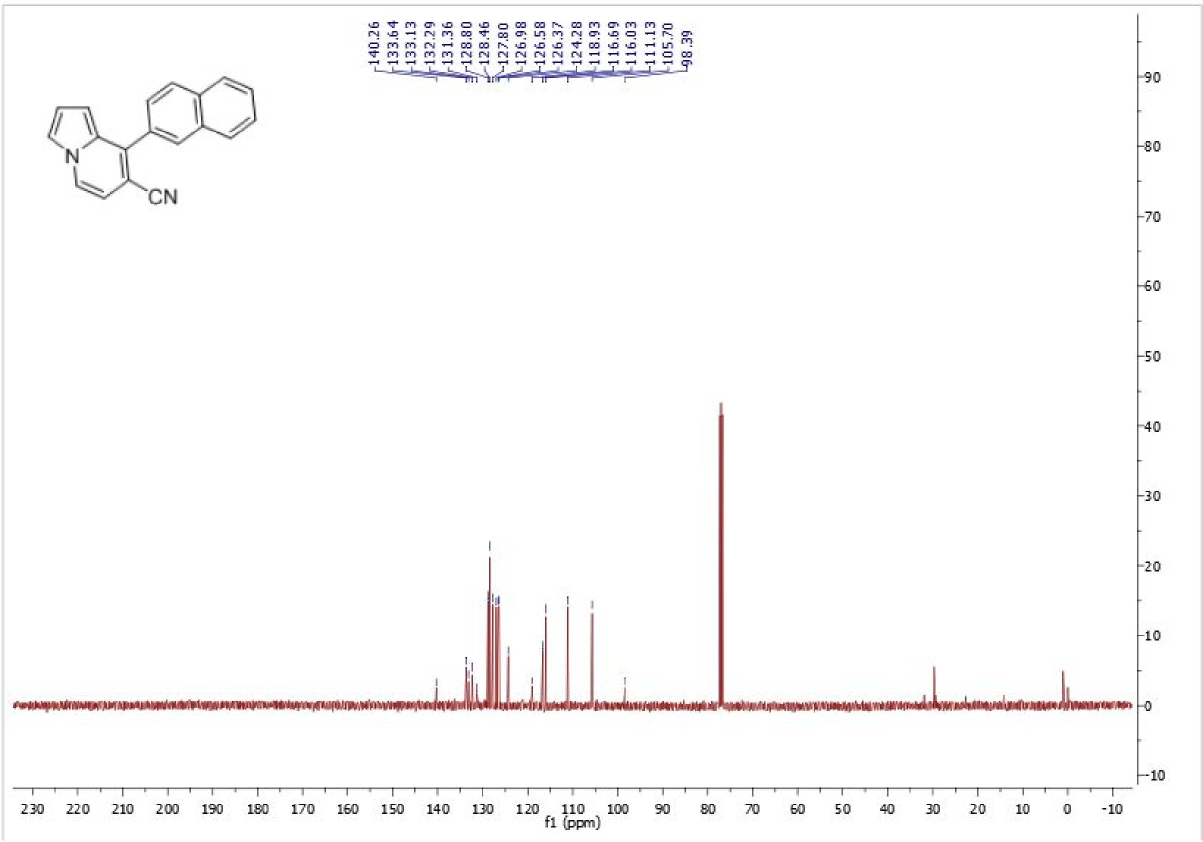

Figure S76

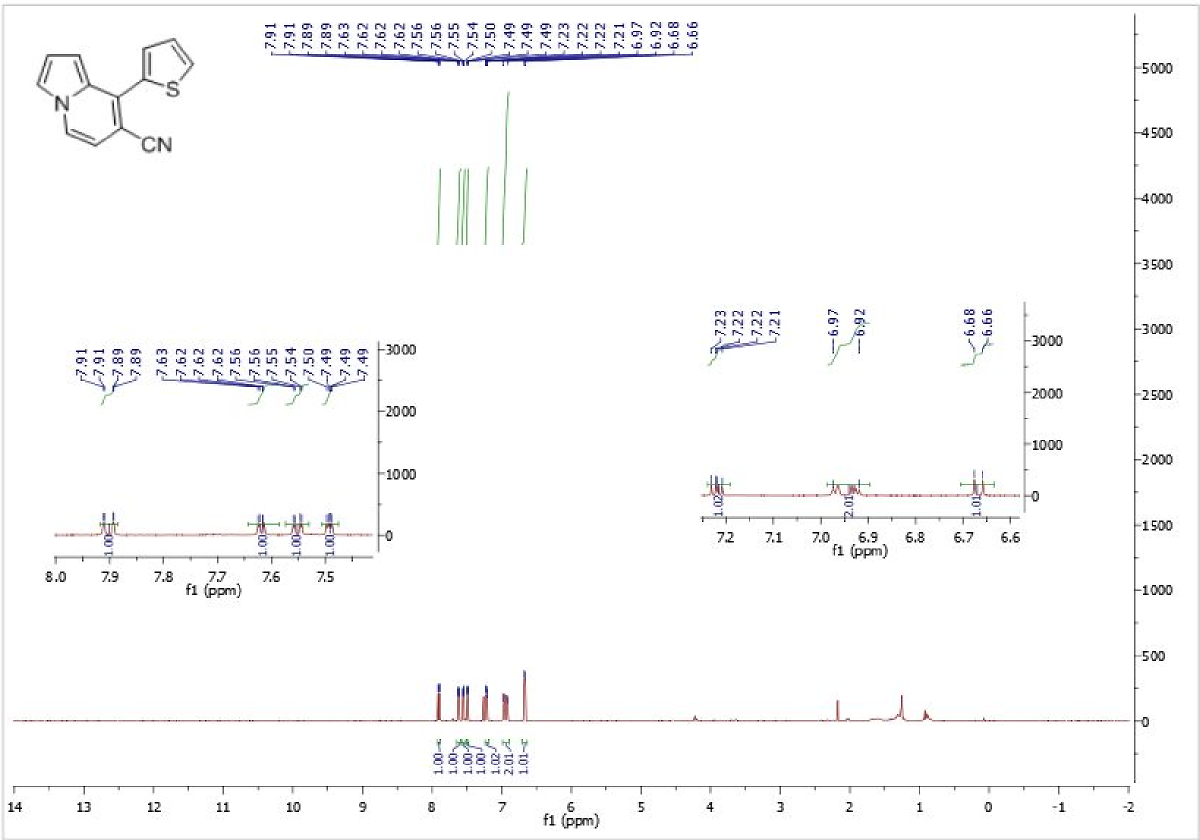

Figure S77.

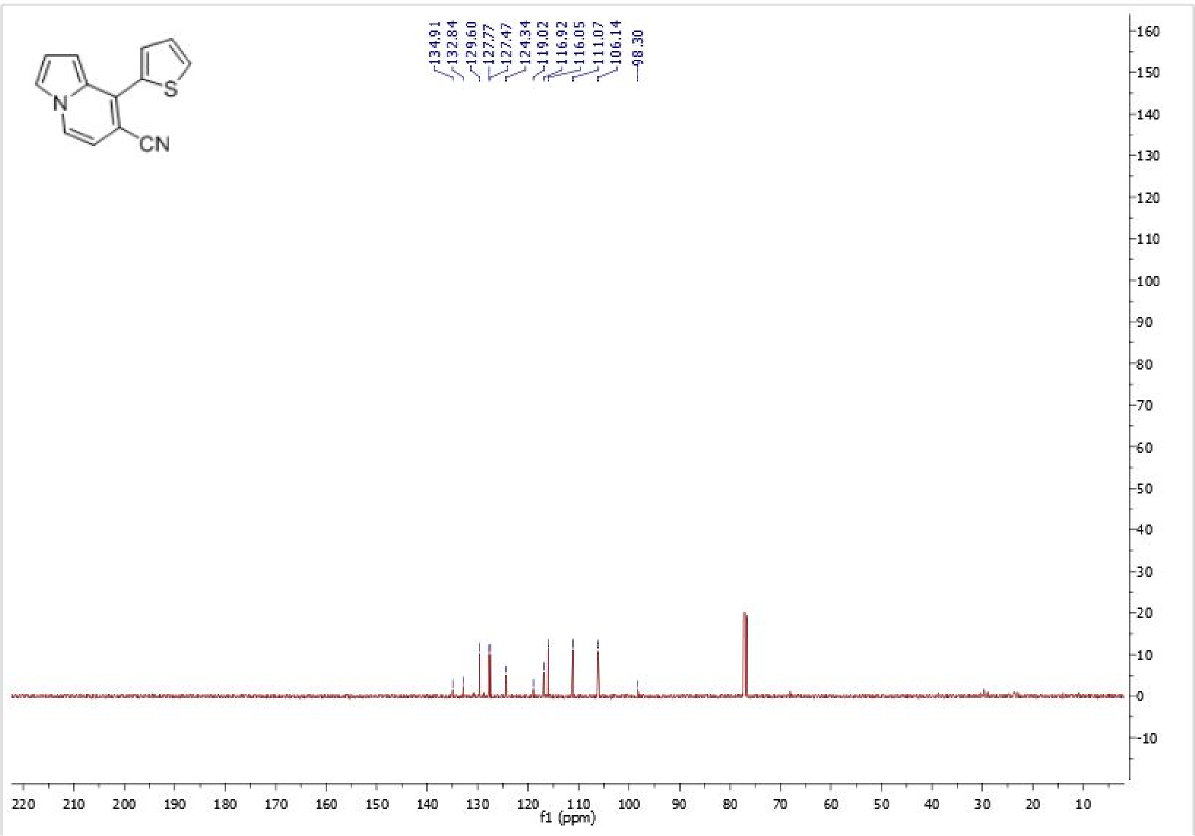

Figure S78

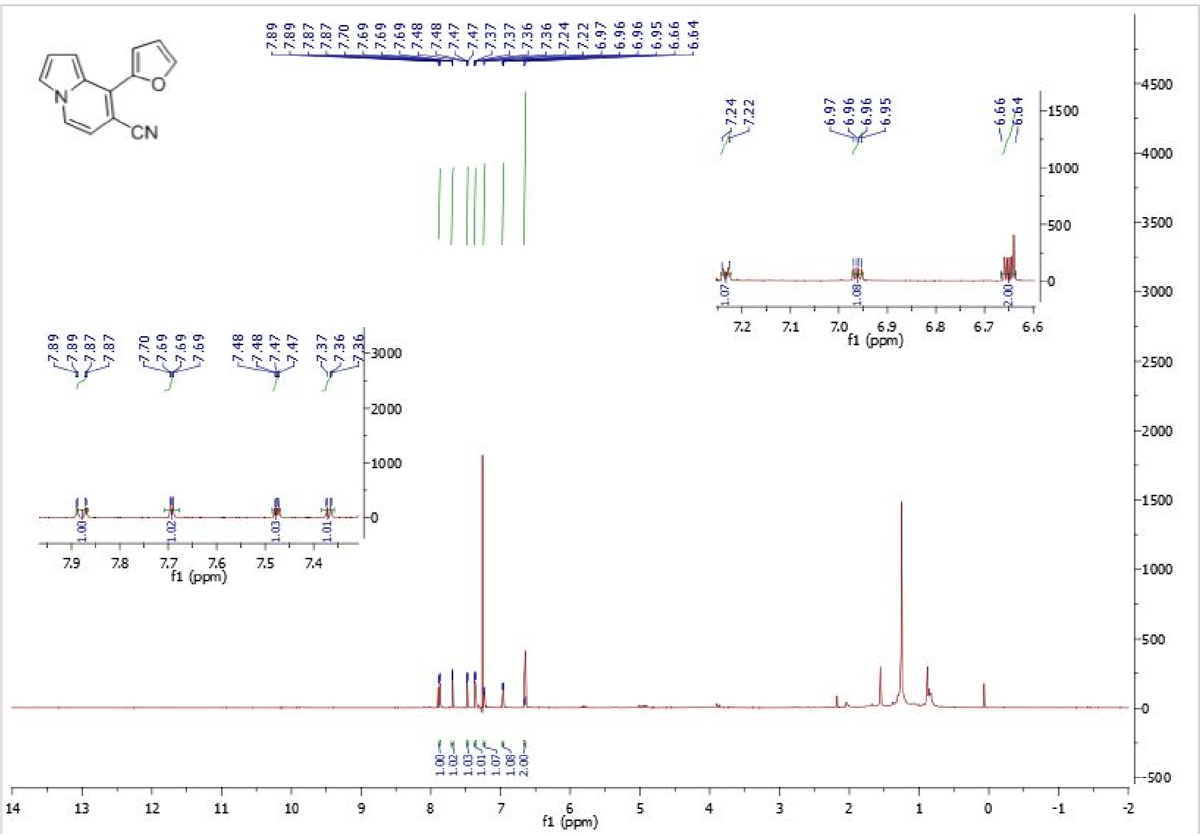

Figure S79.

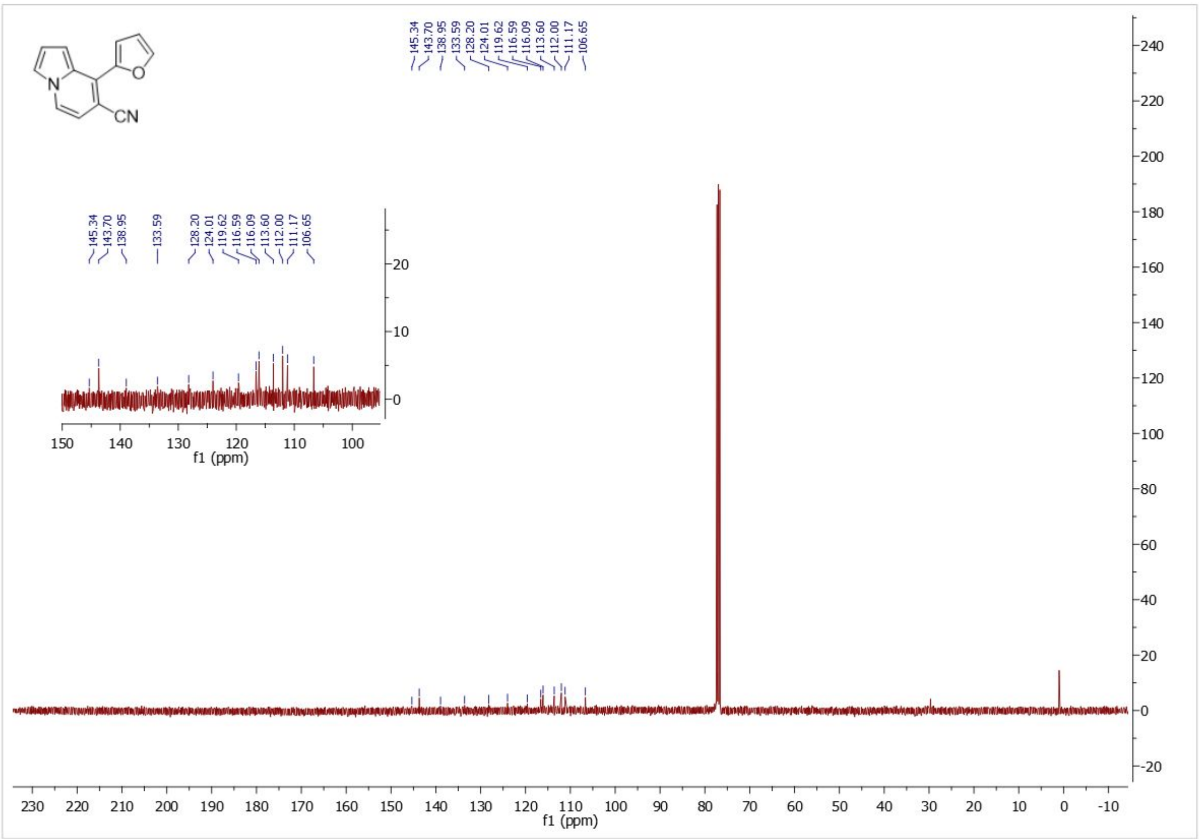

Figure S80.

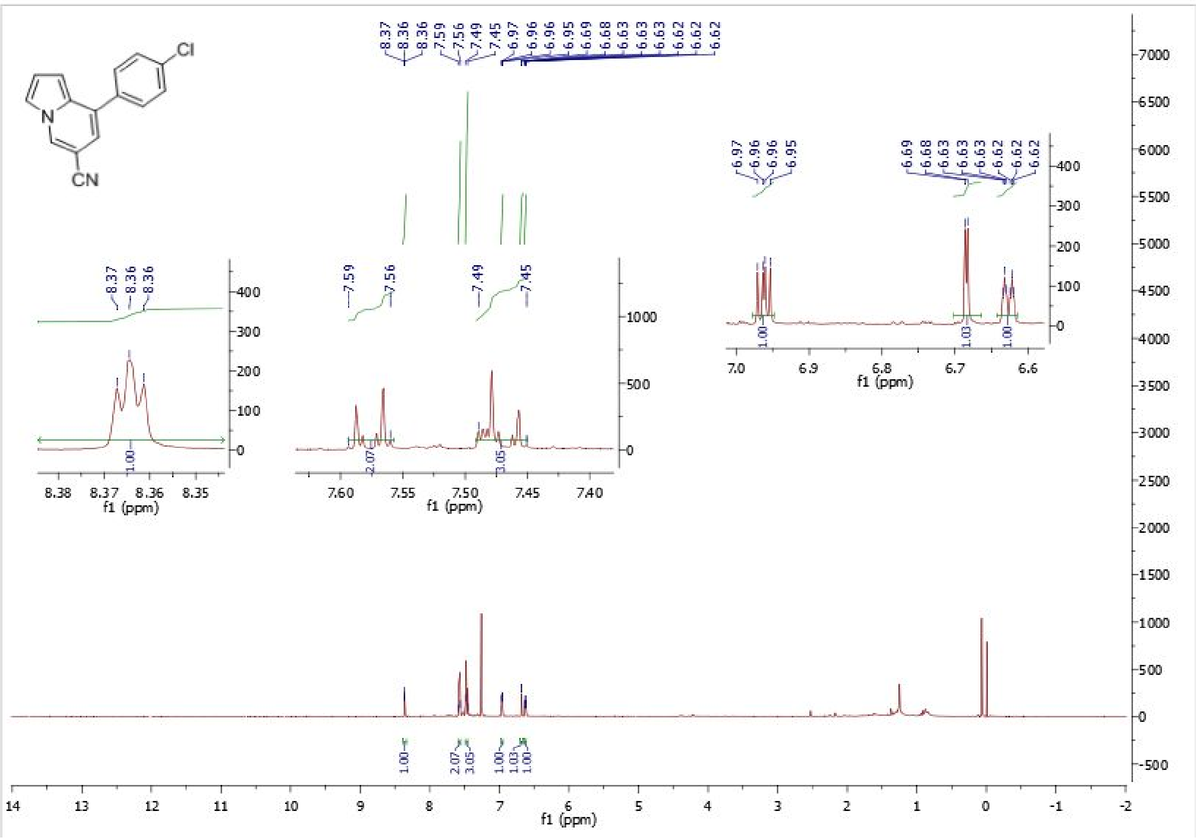

Figure S81.

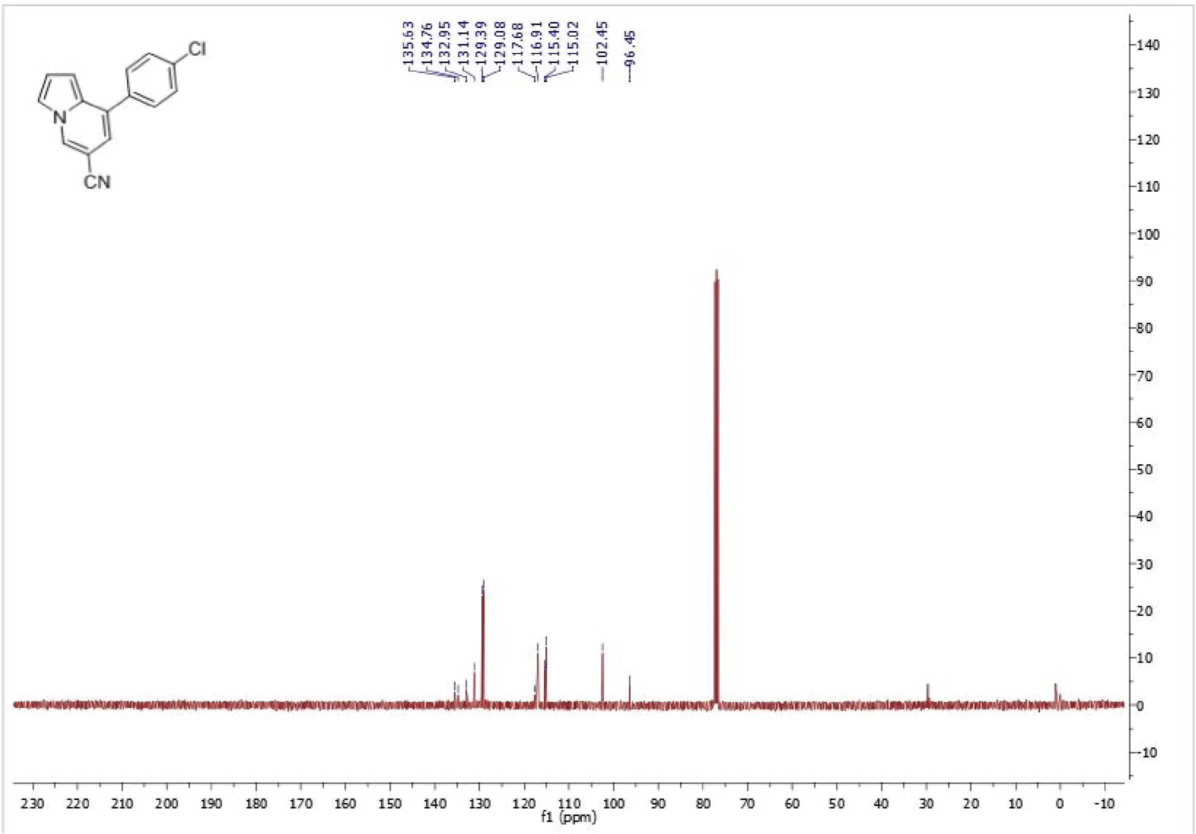

Figure S82.

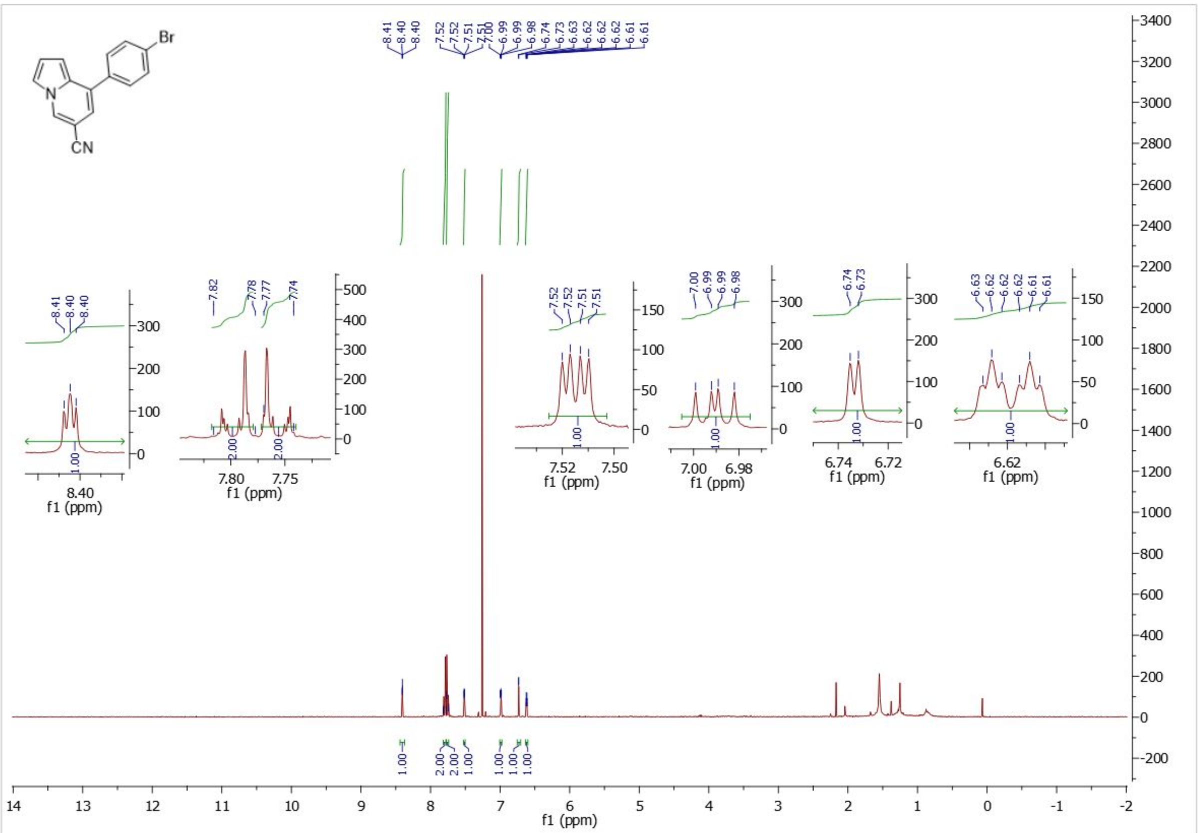

Figure S83

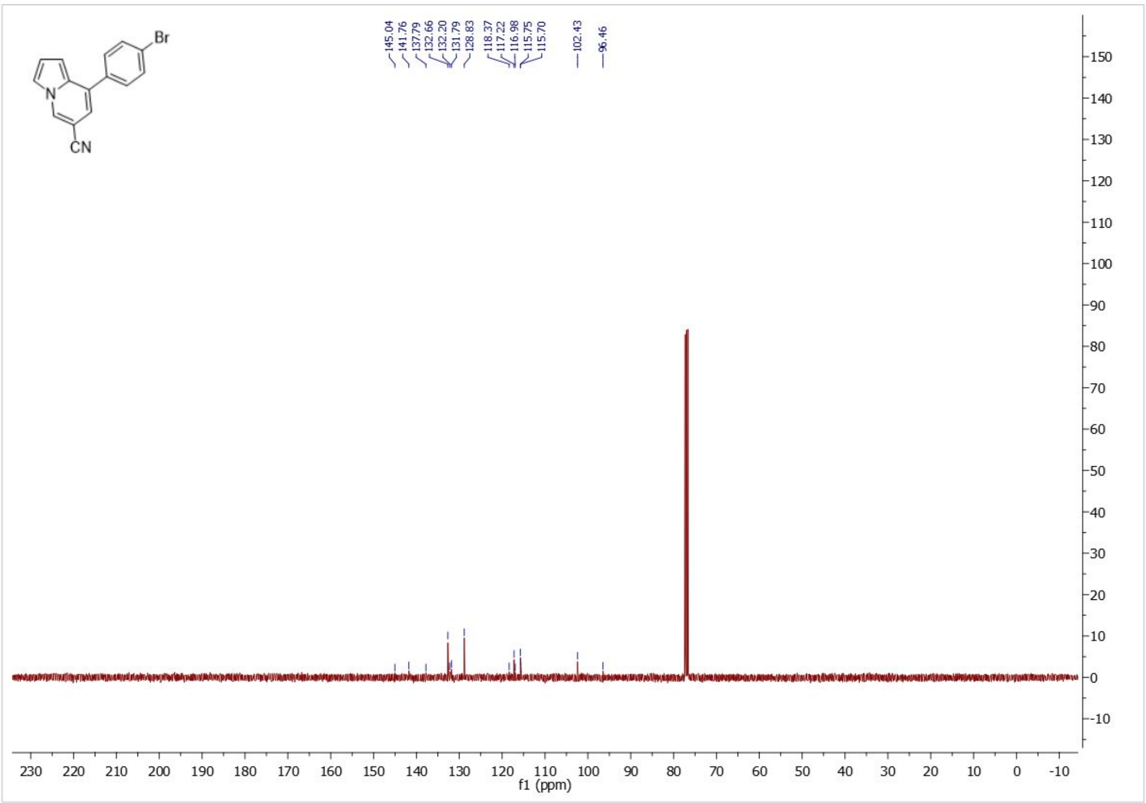

Figure S84.

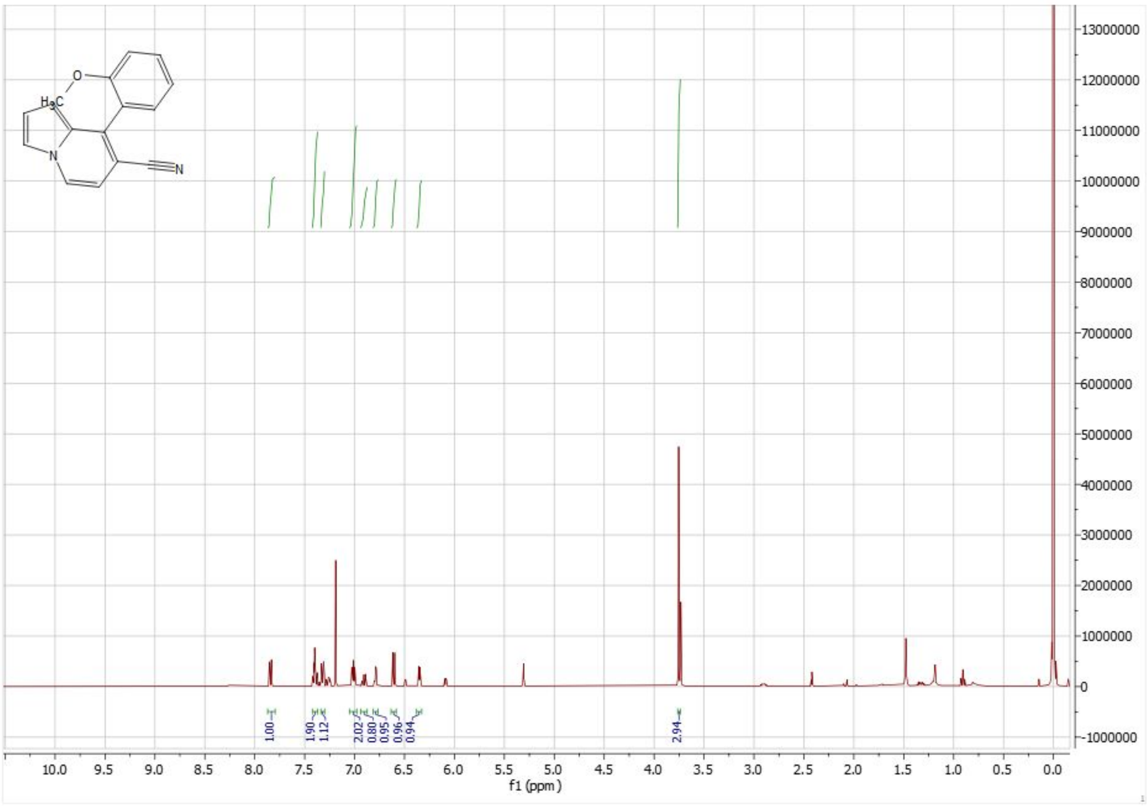

Figure S85.

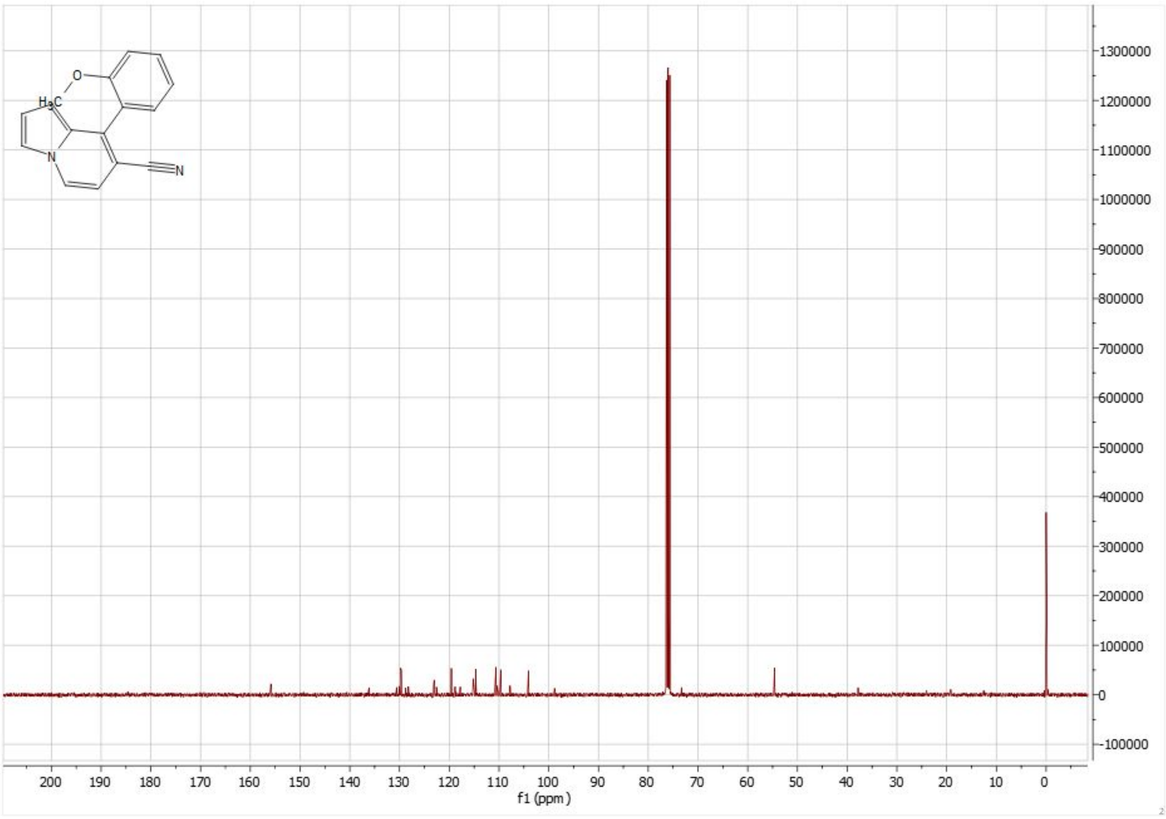

Figure S86.

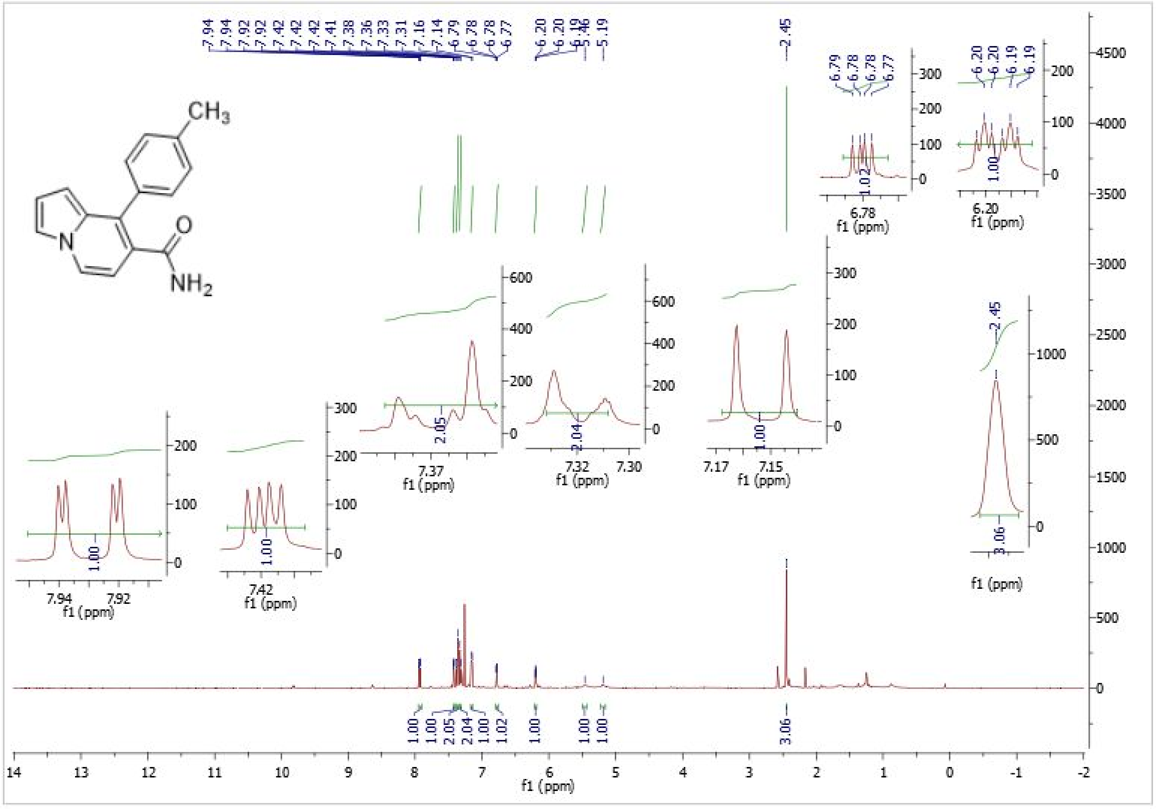

Figure S87.

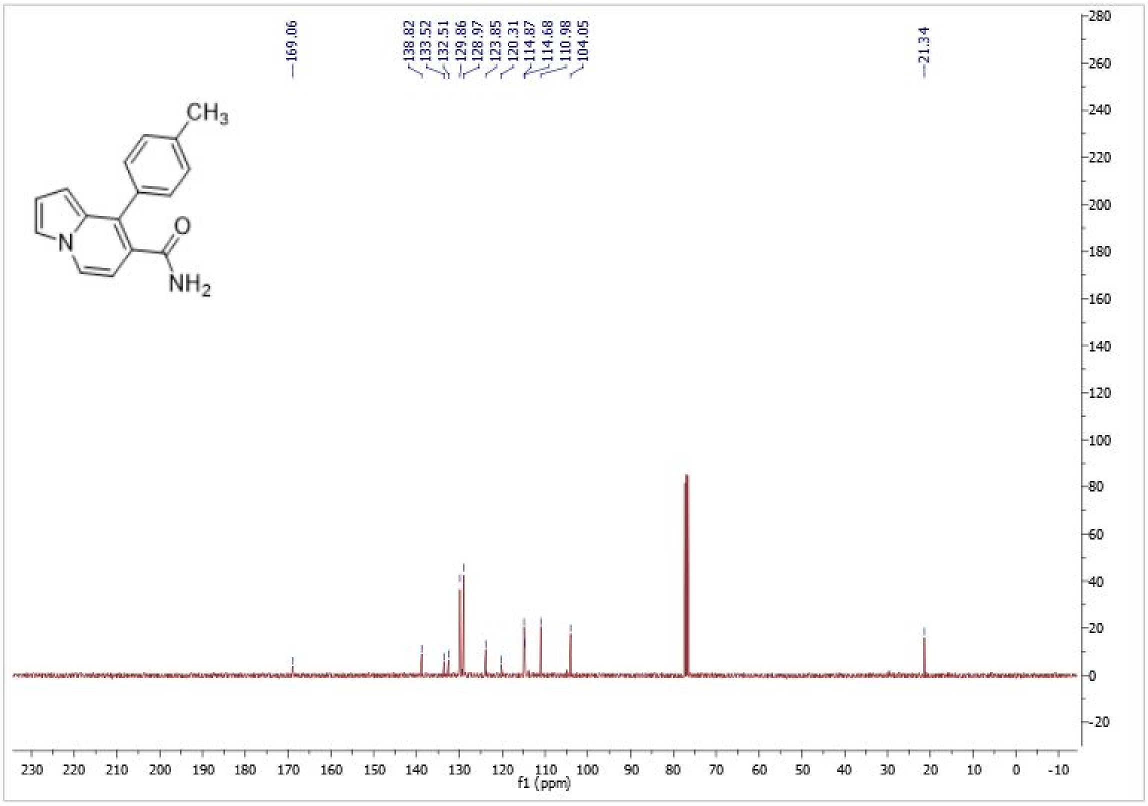

Figure S88.

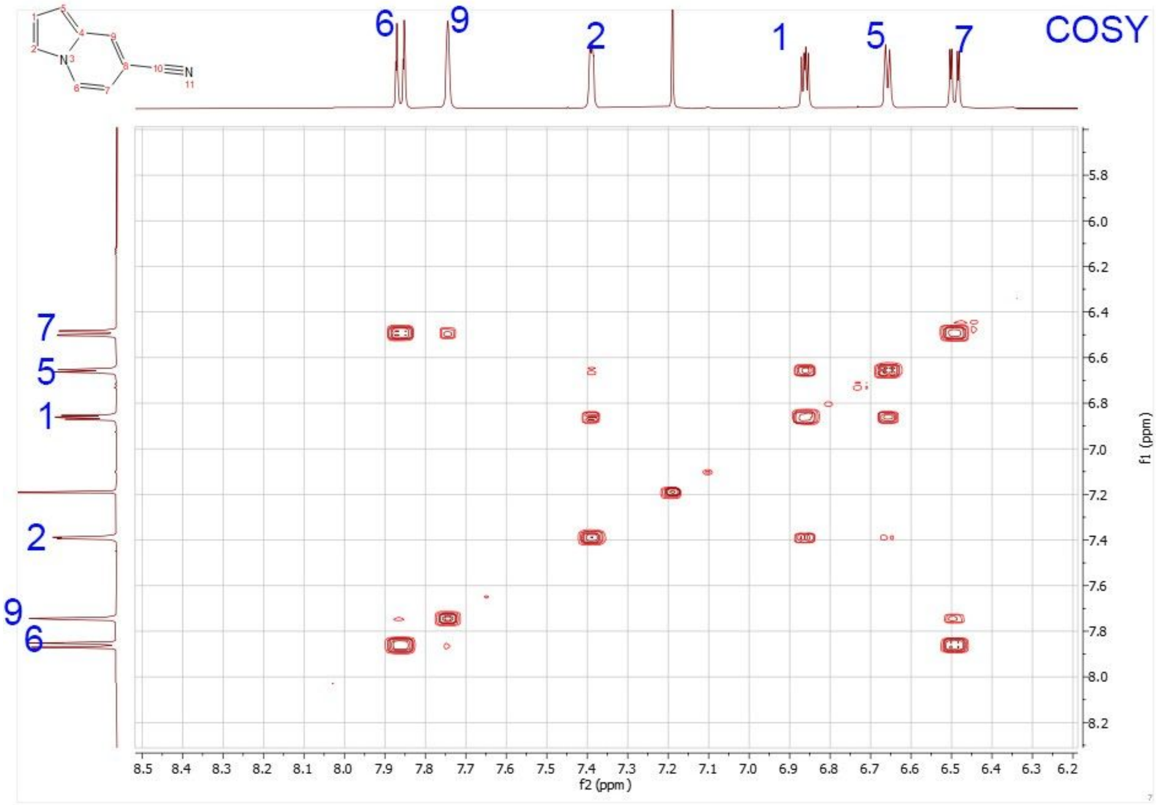

Figure S89. COSY spectrum for compound **3b**

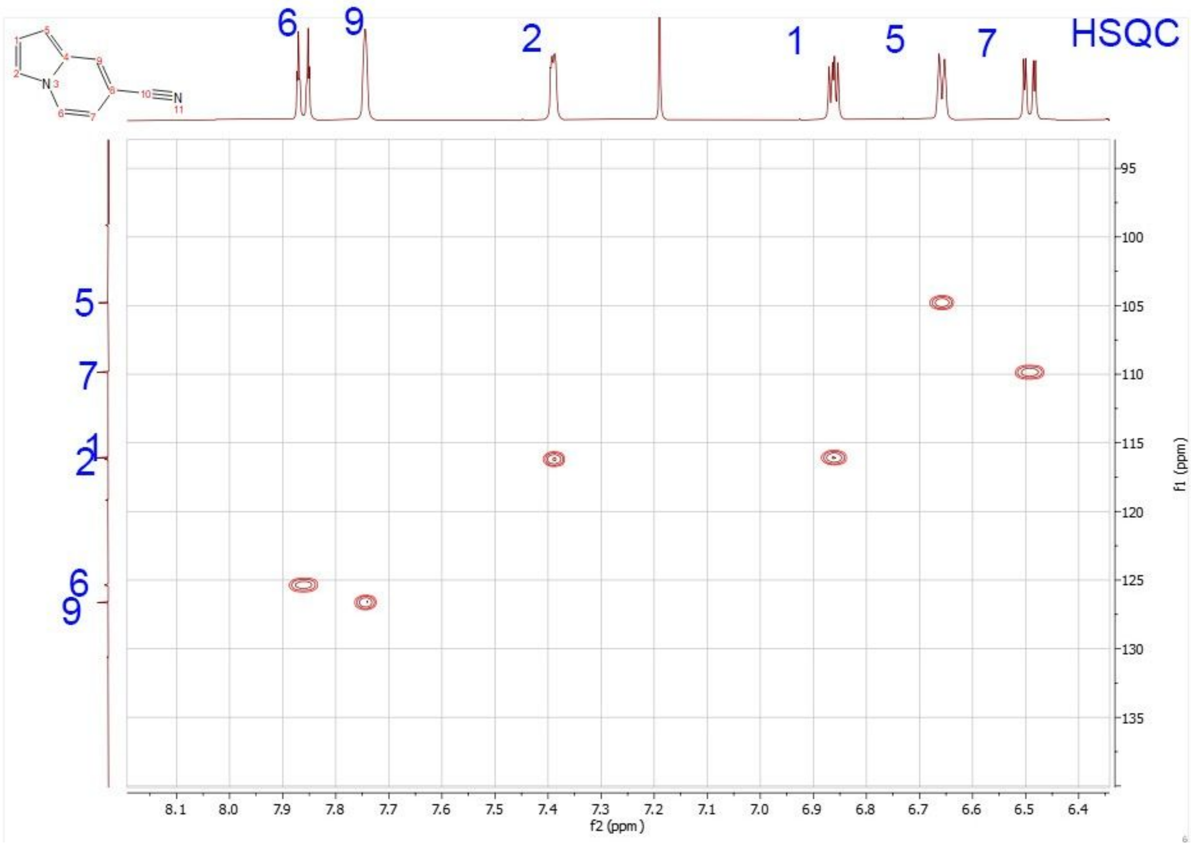

Figure S90. HSQC spectrum for compound **3b**

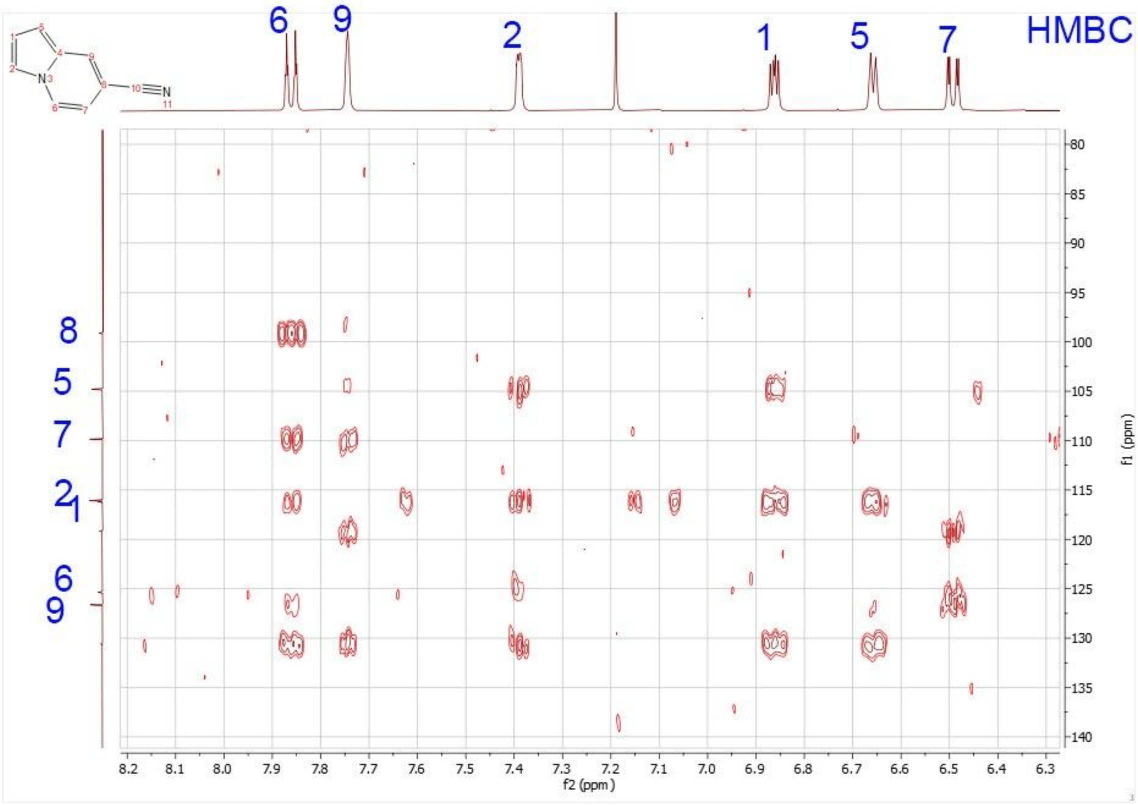

Figure S91. HMBC spectrum for compound **3b**

Reference:

1. Kuzu, B., Gül, S., Tan, M., Menges, N., & Balci, M. (2021). Synthesis of Indolizines by Dimerization of N-Propargylated Pyrroles via Allene Intermediates. *ChemistrySelect*, *6*(9), 2366-2372.
